# Identification and characterization of human *KALRN* mRNA and Kalirin protein isoforms

**DOI:** 10.1093/cercor/bhae470

**Published:** 2024-12-04

**Authors:** Arne W Mould, David J Wright, Klaus D Bornemann, Bastian Hengerer, Rob Pinnock, Edward Drydale, James Bancroft, Nicola A L Hall, Annette von Delft, Paul E Brennan, Paul J Harrison, Wilfried Haerty, Elizabeth M Tunbridge

**Affiliations:** Department of Psychiatry, University of Oxford, Warneford Hospital, Oxford OX3 7JX, United Kingdom; Oxford Health NHS Foundation Trust, Warneford Hospital, Oxford OX3 7JX, United Kingdom; Earlham Institute, Norwich Research Park, Norwich NR4 7UZ, United Kingdom; Boehringer Ingelheim Pharma GmbH & Co. KG, 65 Birkendorfer Straße, 88397, Biberach an der Riß, Germany; Boehringer Ingelheim Pharma GmbH & Co. KG, 65 Birkendorfer Straße, 88397, Biberach an der Riß, Germany; Biogen Idec Ltd, 5 Roxborough Way, Maidenhead SL6 3UD, United Kingdom; Centre for Human Genetics, Nuffield Department of Medicine, University of Oxford, Roosevelt Drive, Oxford OX3 7BN, United Kingdom; Centre for Human Genetics, Nuffield Department of Medicine, University of Oxford, Roosevelt Drive, Oxford OX3 7BN, United Kingdom; Department of Psychiatry, University of Oxford, Warneford Hospital, Oxford OX3 7JX, United Kingdom; Oxford Health NHS Foundation Trust, Warneford Hospital, Oxford OX3 7JX, United Kingdom; Centre for Medicines Discovery, NDM Research Building, University of Oxford, Roosevelt Drive, Oxford OX3 7FZ, United Kingdom; Alzheimer’s Research UK Oxford Drug Discovery Institute, Centre for Medicines Discovery, NDM Research Building, University of Oxford, Roosevelt Drive, Oxford OX3 7FZ, United Kingdom; Department of Psychiatry, University of Oxford, Warneford Hospital, Oxford OX3 7JX, United Kingdom; Oxford Health NHS Foundation Trust, Warneford Hospital, Oxford OX3 7JX, United Kingdom; Earlham Institute, Norwich Research Park, Norwich NR4 7UZ, United Kingdom; School of Biological Sciences, University of East Anglia, Norwich Research Park, Norwich NR4 7TJ, United Kingdom; Department of Psychiatry, University of Oxford, Warneford Hospital, Oxford OX3 7JX, United Kingdom; Oxford Health NHS Foundation Trust, Warneford Hospital, Oxford OX3 7JX, United Kingdom; Boehringer Ingelheim Pharma GmbH & Co. KG, 65 Birkendorfer Straße, 88397, Biberach an der Riß, Germany

**Keywords:** synaptic plasticity, RhoGTPase, GEF, alternative splicing

## Abstract

Kalirin is a multidomain protein with important roles in neurite outgrowth, and synaptic spine formation and remodeling. Genetic and pathophysiological links with various neuropsychiatric disorders associated with synaptic dysfunction and cognitive impairment have sparked interest in its potential as a pharmacological target. Multiple Kalirin proteoforms are detected in the adult human brain, yet we know little about the diversity of the transcripts that encode them or their tissue profiles. Here, we characterized full-length *KALRN* transcripts expressed in the adult human frontal lobe and hippocampus using rapid amplification of complementary DNA (cDNA) ends and nanopore long-read sequencing. For comparison with non-neural tissue, we also analyzed *KALRN* transcripts in the aorta. Multiple novel isoforms were identified and were largely similar between the two brain regions analyzed. Alternative splicing in the brain results in preferential inclusion of exon 37, which encodes 32 amino acids upstream of the second guanine nucleotide exchange factor (GEF) domain. Structural modeling predicts that a subset of these amino acids forms a conserved alpha helix. Although deletion of these amino acids had little effect on GEF activity, it did alter Kalirin-induced neurite outgrowth suggesting that this brain-enriched splicing event may be important for neural function. These data indicate that alternative splicing is potentially important for regulating Kalirin actions in the human brain.

## Introduction

Kalirin is a diffuse B-cell lymphoma (Dbl) family protein encoded by the *KALRN* gene. Dbl family proteins characteristically enhance the activation of RhoGTPases by functioning as guanine nucleotide exchange factors (GEFs). RhoGTPases are molecular switches, existing in either an active guanosine triphosphate (GTP) bound or inactive guanosine diphosphate (GDP) bound state. GEFs bind to RhoGTPases to facilitate GDP release and GTP binding. RhoGTPases are centrally involved in cytoskeletal remodeling associated with cytokinesis, cell polarity, and cell motility (Hodge and Ridley 2016). In neurons, they regulate neurite growth, synaptic spine formation, and synaptic plasticity (Zhang et al. 2021). Kalirin binds and activates the RhoGTPases Rac1, RhoG, and RhoA, all of which have recognized roles in neurite and/or synaptic spine growth (Govek et al. 2005; Kim et al. 2011). Notably, *KALRN* knockout mice display reduced synaptic spine number and impaired long-term potentiation, suggesting that Kalirin is a regulator of synapse number and synaptic plasticity, presumably via effects on RhoGTPase activity and NMDA receptor function (Ma et al. 2008; Cahill et al. 2009; Kiraly et al. 2011). In humans, the *KALRN* gene has been linked with a variety of neurological and psychiatric disorders associated with synaptic dysfunction or cognitive impairment (Remmers et al. 2014; Russell et al. 2018; Leblond et al. 2019; Gulsuner et al. 2020).

**Fig. 1:**
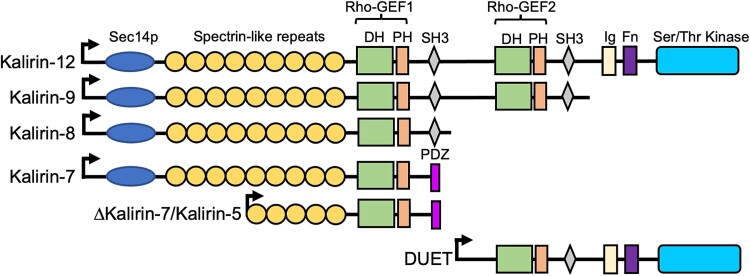
Structure of the major canonical Kalirin proteoforms and their domains. The different domains are color-coded.

Multiple Kalirin proteoforms have been identified that vary in the number and type of domains that they contain, their cellular localization, and their substrate specificities (Mould et al. 2022) (Fig. 1A). Early nomenclature for the different proteoforms was based on the apparent size of the encoding RNA transcript in kilobases in northern blot experiments (Johnson et al. 2000). The longest proteoform, Kalirin-12, consists of an N-terminal lipid-binding CRAL-TRIO/SEC14 domain, followed by 9 spectrin repeats, a Rac1/RhoG guanine nucleotide exchange factor (GEF) domain, an SH3 domain, a second GEF domain that binds to and activates RhoA, a second SH3 domain, an immunoglobulin-like domain, a fibronectin III domain, and a tyrosine kinase domain.

Each of the GEF domains consists of a catalytic Dbl-homology (DH) domain and a regulatory Pleckstrin homology (PH) domain. Alternative transcription end sites (TES) give rise to Kalirin-9, -8, and -7 proteoforms. Kalirin-8 and -9 are essentially truncated versions of Kalirin-12, lacking the domains downstream of the first and second SH3 domains, respectively. In contrast, the terminal exon of *KALRN-7* transcripts encodes a unique PDZ binding domain that localizes the protein to the postsynaptic density (Penzes et al. 2000). Alternative transcription start sites (TSSs) give rise to transcripts encoding proteins with unique N-terminal sequences or, in the case of the so-called delta (Δ) and *DUET* intragenic TSSs, encode proteins that lack the N-terminal CRAL-TRIO/SEC14 domain plus the first 4 spectrin repeats or lack N-terminal domains up to and including the first GEF domain, respectively. Across tissues, *DUET* transcripts are most strongly expressed in skeletal muscle (Kawai et al. 1999).

In rodents, the longer dual GEF Kalirin proteoforms play a prominent role in neurogenesis and early brain development, while the shorter Kalirin-7 protein with a single GEF domain is thought to be responsible for the majority of kalirin signaling in adult brain (McPherson et al. 2002). Notably, Kalirin-7 localizes to dendritic spines and upregulation of Kalirin-7 postnatally coincides with spinogenesis, suggesting a role for this proteoform in synaptic development and function (Penzes et al. 2000; McPherson et al. 2002). In humans, both short and long Kalirin proteoforms are readily detected in the adult brain (Deo et al. 2012). Rodent long and short Kalirin proteoforms have different effects on synaptic spine number and shape (Penzes et al. 2001; Deo et al. 2012). Thus, it is likely that the level of each proteoform also requires careful balancing in the human brain and that altering the levels of individual proteoforms could have deleterious consequences (Deo et al. 2012; Remmers et al. 2014; Mould et al. 2022).

While studies demonstrate that multiple Kalirin proteins of different sizes are expressed in the adult human brain, we know very little about the sequence structure of these proteins and whether they are the same as those expressed in other parts of the body. To answer this question, we investigated the encoding transcripts using a combination of rapid amplification of cDNA ends (RACE) and long-read sequencing to analyze the repertoire of *KALRN* transcripts expressed in two brain regions and for comparison, aorta, a peripheral tissue with high *KALRN* expression (Fig. S1). For the brain regions, we selected the frontal lobe and hippocampus, based on their high *KALRN* expression (Fig. S1) and dependence on Kalirin signaling (Ma et al. 2008; Cahill et al. 2009). We then functionally characterized the proteins encoded by the most abundant *KALRN* transcript isoforms and compared their catalytic activity and cellular effects.

## Materials and methods

### Rapid amplification of cDNA ends

5′ and 3’ Rapid Amplification of cDNA Ends (RACE) for *KALRN* was performed using polyA+ RNA purified from pools of the adult human frontal lobe (Takara Bio, catalog number 636165, lot # 1506688A), hippocampus (Takara Bio, catalog number 636134, lot # 9100127A), and aorta (Takara Bio, catalog number 636153, lot # 812114A) using a 5′/3′ RACE Kit, 2nd Generation (Roche Diagnostics). Polymerase chain reactions (PCRs) were performed using Illustra PuReTaq Ready-To-Go PCR beads (Cytiva) or PrimeSTAR GXL DNA Polymerase (Takara Bio) for 5′ and 3′ RACE, respectively. Primers were designed using Primer3 plus software (http://primer3plus.com), and the sequences are available in Table S1. RACE products were separated on 2% agarose/Tris-acetate-Ethylenediaminetetraacetic acid (TAE) gels containing 1× GelGreen (Biotium), the individual amplicon bands excised, and gel purified using a Monarch DNA Gel Extraction Kit (New England Biolabs) and cloned using TOPO TA or Zero Blunt TOPO PCR cloning kits for Sequencing, with One Shot TOP10 Chemically Competent *Escherichia coli* (Invitrogen). Multiple clones per amplicon were Sanger-sequenced (Cambridge Bioscience) and aligned to the human genome (hg38) using BLAT (Kent 2002). Novel sites were called if supported by a minimum of two overlapping clones from any of the 3 tissues (aorta, frontal lobe, or hippocampus).

### Full-length transcript PCR

First-strand cDNA was reverse-transcribed from 100 ng of polyA+ RNA using a Maxima H Minus Reverse Transcriptase kit (Thermo Scientific) with oligo (dT)_18_ priming, according to the manufacturer’s recommendations. cDNA was diluted one-fourth in nuclease-free water, and 1 μl was used as a template per 25 μl PCR reaction. Full-length *KALRN* transcripts were amplified by touchdown PCR using PrimeSTAR GXL DNA Polymerase (Takara Bio). After an initial hold for 2 min at 98°C, samples were run for two thermocycles of denaturing at 98°C for 10 s, annealing at 65°C for 15 s, and extension at 72°C for 1 min/kb of predicted transcript length. This was followed by a further 8 cycles, where the annealing temperature was dropped by 1°C every 2 cycles and finished with 25 cycles at an annealing temperature of 60°C followed by a final hold for 5 min at 72°C. PCR products were separated on 1% agarose/TAE/ethidium bromide (EtBr) gels with 10 μl of 1 kb DNA ladder (New England Biolabs). Images were captured using an AlphaImager 3400 (Alpha Innotech), and amplicon band intensities were determined by densitometry using Image J, relative to the intensity of the 3 kb band of the DNA ladder. Each PCR amplicon potentially contains multiple splice variants not easily resolved by gel electrophoresis and are referred to as amplicon pools throughout this manuscript.

### Sequencing library preparation and nanopore long-read sequencing

For targeting sequencing, full-length transcripts were amplified by touchdown-PCR as described above using primers with added barcoding adaptor sequences (see Table S2 for details). Difficult-to-amplify targets were pre-amplified for 25 cycles with primers without barcode adaptors, column-purified using a Monarch PCR clean-up kit (NEB), and then amplified for 10 cycles with primers containing adaptor sequences.

PCR products were electrophoresed through a 1% agarose/TAE gel, and the amplicons were purified using a Monarch DNA Gel Extraction Kit (NEB). The barcoding PCR was performed on the purified products with PrimeSTAR GXL (Takara) and PCR Barcoding Expansion 1-96 (EXP-PBC096, ONT), for 15 cycles. The barcoded products were purified using a Monarch PCR clean-up kit (NEB) and the DNA size distribution was assessed using genomic DNA ScreenTape (Agilent). After quantifying using a Broad Range DNA assay (Qubit), barcoded samples (individual amplicons consisting of a pool of splice variants) were combined in equal amounts into one of two sequencing pools. Sequencing pool 1 contained samples <4 kb and sequencing pool 2 contained samples >4 kb. Each sequencing pool was bead purified to remove low-molecular-weight DNA using 0.5× and 0.45× Bio-Tek Mag-Bind Total Pure NGS beads (Omega) for groups 1 and 2, respectively, and the product was assayed for size and concentration using genomic DNA ScreenTape (Agilent) and a Broad-Range DNA assay (Qubit). For each sequencing pool, DNA product (1 μg) was prepared using a Ligation Sequencing kit SQK-LSK109 and sequenced on a FLO-MIN106D flow cell (Oxford Nanopore Technologies).

### Sequencing analysis and novel exon identification

Sequencing bases were called using guppy (v4.0.11). For each primer set independently, we identified, annotated, and quantified the relative expression of the *KALRN* transcripts using the TAQLoRe pipeline (Clark et al. 2020) (https://github.com/twrzes/TAQLoRe, https://taqlore.readthedocs.io/en/latest/). Briefly, reads passing quality control (QC) and with an identified barcode were mapped to the transcriptome (GENCODE v36) using LAST (v926) (Frith et al. 2010). Novel exons/splicing events were identified relative to the reference transcriptome, with a minimum 50% of a read mapping and supporting at least 80% of the transcript and a minimum insert length of 9 nucleotides. The genomic coordinates of these novel events were retrieved by mapping to the genome (hg38) using LAST. Supported novel exons were then incorporated into the *KALRN* metagene model prior to remapping the reads using GMAP (v2019-03-15) to enable their characterization, annotation, and quantification of relative expression. Novel exons were filtered by parsing the mapping Compact Idiosyncratic Gapped Alignment Report (CIGAR) strings and retaining only exons with a minimum length of 6 nucleotides and at least 70% support from the sequencing reads. All transcript isoforms were checked and retained if they possessed at least 1 read per sample and found to be expressed in at least 5 libraries. Finally, the down-weighted reads counts were filtered at two stringencies: minimum 100 reads and minimum 24 reads across all libraries to assess experimental sensitivity in recovering transcripts, following Clark *et al*. (Clark et al. 2020). Transcript quantification was tmm-normalized across all samples.

To validate the novel splicing events detected, we used publicly available human GTEx data (https://www.gtexportal.org). Comparison between tissues (e.g. cerebral cortex vs aorta, *n* = 42) was achieved by selecting paired libraries, where each tissue was sequenced from the same individual. Exon 37 percent-spliced-in (PSI) was calculated using read counts supporting the *KALRN* exon junctions after mapping the different libraries to the reference hg38 genome using STAR (v2.6) (Dobin et al. 2013). We focused on the junctions supporting the exon 37 skipping (chr3:124633954-124650807) and those supporting its inclusion (chr3:124633954-124637207, chr3:124637304-124650807). PSI was computed as: [Inclusion reads/(Exclusion reads + Inclusion reads)] × 100. Comparison between tissues was performed using a Fisher exact test.

### Expression vectors

The top-ranked splice isoforms of the most abundant *KALRN*-*7, -9,* and -*12* full-length transcripts in frontal lobe and aorta sample pools were synthesized with an N-terminal DYKDDDDK peptide (FLAG)-tag separated by a 5-glycine spacer by GeneArt (Thermo Fisher Scientific) and cloned into pcDNA3.1 expression vector (Invitrogen) (Fig. 4). Two additional isoforms of brain *KALRN-9* and -*12* that lacked exon 37 were cloned by swapping restriction enzyme digest fragments between vectors. Briefly, the *Pml1/EcoRV* restriction digest fragment (containing exon 37) from the brain *KALRN-9* and -*12* vectors was excised and replaced by ligation with the *Pml1/EcoRV* fragment from the aorta *KALRN-12* vector (skipping exon 37 and identical to the same region in aorta *KALRN-9*). Isoform coding sequences for the expression constructs are available in supplementary information (Fig. S2). Expression vectors for brain and aorta dual GEF region proteins were prepared by PCR amplification of the region spanning both GEF domains from aorta and brain Kalirin-9 expression vectors using primers designed to add an N-terminal FLAG-tag and restriction enzyme sites to facilitate cloning. PCR products were gel-purified, restriction enzyme–digested, and cloned into pcDNA3.1. Dual GEF region vectors minus the PH2 encoding region (GEF1-GEF2ΔPH2) were generated in a similar fashion.

All vectors were assessed for FLAG-tagged protein expression by transfecting them into HEK293T cells (ATCC) using lipofectamine 3000 (Thermo Fisher Scientific). RIPA lysates were prepared 24 to 48 h post-transfection and analyzed by Western blot using anti-FLAG M2 antibody (Sigma-Aldrich). All vectors expressed FLAG-tagged proteins of the expected size.

### Cell culture

HEK293T (ATCC) and SH-SY5Y cells were grown on Cell+ tissue culture dishes (Sarstedt) of various sizes in growth media (Dulbecco’s Modified Eagle Medium, high glucose [DMEM, Gibco] supplemented with 10% fetal bovine serum [Lonza, DE14-801FH], 1× Glutamax [Gibco], and 1× penicillin/streptomycin [Gibco]). Cultures were passaged every 3 to 4 days using 0.25% Trypsin-Ethylenediaminetetraacetic acid (EDTA) (Gibco) to detach the cells.

### Transfections, protein expression, and purification

For protein expression analysis, HEK293T cells were seeded into 35 mm diameter Cell+ culture dishes (Sarstedt) at a density of 5.5 × 10^4^ cells/cm^2^ in growth media without antibiotics and cultured overnight in a humid incubator at 37°C with 5% CO_2_. The following day, cultures were transfected with 2.5 μg of plasmid using Lipofectamine 3000, according to the manufacturer’s protocols, and then placed back in the incubator. Cells were harvested at 24, 48, and 72 h post-transfection. Briefly, the medium was aspirated, the cultures were rinsed once with Dulbecco’s phosphate-buffered saline minus calcium and magnesium (DPBS, Gibco), and the cells were lysed in ice-cold RIPA buffer (Merck) containing 1× EDTA-free complete protease inhibitor cocktail (Roche) on ice. Lysates were cleared by centrifugation at 10,000 relative centrifugal force (RCF) for 15 min at 4°C and the supernatants were aliquoted, snap-frozen on dry ice, and stored at −80°C.

To analyze the effects of exogenous expressed Kalirin proteoforms on endogenous RAC1 and RHOA activation, HEK293T cells were seeded into 60 mm diameter Cell+ culture dishes at a density of 9 × 10^4^ cells/cm^2^ in growth media without antibiotics and cultured overnight. The following morning, culture media was removed, and the cells were gently washed with prewarmed DMEM supplemented with 1× Glutamax. The media was then replaced with fresh prewarmed DMEM supplemented with 1× Glutamax, and the cells were transfected with 6 μg of plasmid using Lipofectamine 3000, according to the manufacturer’s protocols. After 12 or 24 h of incubation at 37°C in 5% CO_2_, cells were harvested, and the levels of activated RAC1 and RHOA in lysates were measured using the appropriate GLISA (Cytoskeleton, Inc.) as per manufacturer’s protocols. As a positive control for RHOA activation, RHOA activator II (Cytoskeleton) was added to control vector-transfected cells (1 μg/ml final concentration) 3 h prior to harvest.

For the production and purification of aorta and brain dual GEF region proteins, HEK293T cells were seeded into 100 mm diameter Cell+ culture dishes (Sarstedt) at a density of 5.5 × 10^4^ cells/cm^2^ in growth media without antibiotics and cultured overnight at 37°C in 5% CO_2_. Duplicate dishes were then transfected with 15 μg of plasmid using Lipofectamine 3000, according to the manufacturer’s protocols. After 48 h of culture, cells were rinsed with DPBS and lysed in 0.5 ml of ice-cold immunoprecipitation (IP) lysis buffer (25 mM Tris-HCl pH 7.4, 150 mM NaCl, 1% TritonX-100, 1 mM EDTA, 5% glycerol) containing 1× EDTA-free Complete protease inhibitor cocktail (Roche) on ice. Lysates were cleared by centrifugation (10,000 RCF for 10 min at 4°C), and the supernatants were transferred to a fresh microfuge tube on ice. After dilution with an equal volume of ice-cold DPBS containing 1× protease inhibitors, samples were mixed by rotation with 125 μl of washed anti-DYKDDDDK Magnetic Agarose beads (Pierce) for 2 h at 4°C. Beads were then washed 3 times with DPBS, and the FLAG-tagged proteins were eluted twice with 125 μl of 1.5 mg/ml triple FLAG-peptide (Pierce) in DPBS. Each elution was performed for 20 min at 4°C with rotary mixing. Eluates were pooled, and NaCl was added to 0.5 M. The proteins were concentrated using Microcon 30K Centrifugal Filter Devices (Merck Millipore), and buffer was exchanged into GEF exchange buffer (20 mM HEPES pH 7.4, 150 mM NaCl, 10 mM MgCl_2_, 5% glycerol, and 1 mM DTT) using Zeba Spin Desalting Columns, 7K MWCO (Pierce). Purified proteins were aliquoted, snap-frozen on dry ice, and stored at −80°C. Protein purity was assessed by SDS-PAGE using 4% to 15% Tris-Glycine eXtended (TGX) gels (BIORAD), poststained with SimplyBlue SafeStain (Invitrogen). FLAG-tagged proteins were quantified by densitometry analysis of protein bands using Fiji (Schindelin et al. 2012) with reference to a bovine serum albumin (BSA) standard curve derived from multiple concentrations of BSA run on the same gel.

Kalirin protein trafficking and effects on cell morphology, including neurite outgrowth were assessed in the SH-SY5Y neuroblastoma cells. For analysis of the early effects of exogenous Kalirin proteoform expression on cell morphology, cells were seeded into 35 mm Cell+ dishes (Sarstedt) at a density of 5.5 × 10^4^ cells/cm^2^ in growth media without antibiotics and cultured overnight at 37°C in 5% CO_2_. Cultures were cotransfected the following morning with 1 μg of pEGFP-N2 (Clontech) plasmid plus 4 μg of plasmid encoding individual Kalirin proteoforms or empty pcDNA3.1 vector, using Xfect transfection reagent (Takara Bio) according to the manufacturer’s protocol. The pEGFP-N2 plasmid encodes a cytoplasmic enhanced green fluorescent protein (EGFP) that facilitates imaging of cell profiles by immunofluorescence. As per the manufacturer’s recommendations, the media was replaced with fresh prewarmed growth media minus antibiotics at 4 h post-transfection. For analysis of neurite outgrowth, cells were seeded into 8-well Lab-Tek II Chamber Slides (Nunc) at a density of 5.5 × 10^4^ cells/cm^2^ in growth media without antibiotics and cultured overnight at 37°C in 5% CO_2_. Cells were then cotransfected with 0.5 μg of pEGFP-N2 (Clontech) plasmid plus 2 μg of plasmid encoding individual Kalirin proteoforms or empty pcDNA3.1 vector, using Lipofectamine 3000 reagent (Invitrogen) according to the manufacturer’s protocol.

At 12 or 48 h post-transfection, for analysis of early morphological changes and neurite outgrowth, respectively, cultures were gently rinsed with prewarmed Hanks’ buffered salt solution containing calcium and magnesium (GIBCO) and fixed, in situ, with 4% paraformaldehyde (PFA) in DPBS for 10 min at room temperature (RT). After gently rinsing with DPBS, cells were processed for immunofluorescence analysis.

### Western blot analysis

Cell lysates and purified proteins were diluted with an equal volume of 2× Laemmli buffer and heat-denatured for 10 min at 90°C. The proteins were separated on 4% to 15% TGX gels (Bio-Rad) by electrophoresis at 90 V for 90 min and then electro-transferred to PVDF membrane for 90 min at 100 V using standard buffers. Protein transfer was confirmed using Ponceau S staining (Merck). Membranes were blocked with 4% nonfat milk powder (Marvel) in DPBS containing 0.1% Tween-20 (PBST), and the target proteins were immunodetected by sequential incubation with primary and appropriate horseradish peroxidase (HRP)-linked secondary antibodies diluted in block solution (see Table S3 for antibody details and working concentrations). Blocking and antibody incubation steps were performed at RT for 1 h each. Triplicate washes in PBST were performed after antibody incubations. Bound secondary antibodies were detected by incubation with ECL prime (Cytiva) for 5 min at RT and exposure to Hyperfilm ECL (Cytiva).

### Immunolabeling, fluorescence microscopy, and confocal imaging

PFA-fixed cells in 35 mm culture dishes (12 h post-transfection) or 8-well culture slides (48 h post-transfection) were permeabilized using 0.5% Triton X-100 in PBS for 15 min at RT, and nonspecific protein binding sites were blocked using 3% BSA in PBS containing 0.1% Triton X-100 (PBSTr) for 1 h at RT. Cells were incubated with primary antibodies diluted in BSA block (mouse anti-FLAG M2 mAb and rabbit anti-GFP Ab) overnight at 4°C. Bound antibody was detected by incubation for 1 h at RT with antimouse IgG Alexa594 and antirabbit IgG Alexa488 conjugates diluted in BSA block (see Table S2 for antibody details and working concentrations). Triplicate washes with PBSTr were performed after each antibody incubation step. Samples were mounted using a drop of ProLong Glass Antifade Mountant with NucBlue stain (Invitrogen) and a number 1 glass coverslip.

For analysis of early changes in cellular morphology, FLAG (red), and GFP (green) immunofluorescence and H33342 fluorescence (blue) images were captured on an Infinity3 camera (Lumenera) mounted on an Eclipse E600 Fluorescence microscope (Nikon) using Infinity Capture (v6.3.2) software. Images were imported into CellProfiler v4.0.7 (Stirling et al. 2021), and the cell outlines were traced using cytoplasmic GFP staining as a guide. For Kalirin expression vector–transfected samples, double FLAG and GFP-positive cells were analyzed. Cell areas (80 to 104 cells per group) were analyzed using in-built functions. Data were imported into SPSS v29.0.1.0 (IBM) for statistical analysis and graphing. Average cell profiles for each group were compiled using Fiji based on cell images exported from the CellProfiler analysis. Cell images were preprocessed using find edge and threshold functions, and individual cell profile images were saved. After centering each cell soma and aligning the trajectories of the largest neurite, average cell profiles were generated by overlaying 80 to 104 cell profiles per group using the Z-project function.

FLAG-tagged and EGFP protein localization and neurite outgrowth were analyzed using confocal microscopy. Images were captured using an Olympus IXplore Spin-SR microscope using a 50 μm pinhole confocal spinning disc and a Hamamatsu ORCA Fusion BT camera. A 60× 1.5NA oil immersion and a 20× UPLXAPO objective lens were used for the acquisition of images for protein trafficking and neurite outgrowth analysis, respectively. Four hundred five, 488, 561, and 640 nm lasers were used with a Quad pass 405/488/561/640 dichroic mirror and barrier filters at 447/60, 525/50, 617/73, 685/40. Z-stack images captured using the 60× objective were imported into Fiji for analysis of protein localization. Single image scans, captured using the 20× objective, were imported into Fiji and separated into green (EGFP) and red (Flag) channels. The images were then imported into CellProfiler for neurite analysis of GFP (empty vector control, *n* = 189 cells) or double GFP/FLAG–positive cells (*n* = 116 to 183 cells per group) using Morph and MeasureObjectSkeleton functions.

### GEF exchange assays

GEF exchange assays were performed in 10 μl assay volume in triplicate in MicroAmp Optical 384-well reaction plates (Applied Biosystems) sealed with MicroAmp Optical adhesive Film (Applied Biosystems). Reactions were initiated by adding 5 μl of 3 μM BODPYFL GTP (Invitrogen) in buffer A (20 mM HEPES pH 7.4, 150 mM KCl, 10 mM MgCl_2_, 5% glycerol, 0.1% Triton X-100, and 1 mM DTT) to 5 μl of buffer B (20 mM HEPES pH 7.4, 150 mM NaCl, 10 mM MgCl_2_, 5% glycerol, and 1 mM DTT) containing 4 μM recombinant human RAC1 or RHOA (Cytoskeleton) plus purified brain or aorta dual GEF region proteins ranging in concentration from 2 nM to 2 μM. Reactions minus GEF were included to determine intrinsic GTP loading rates for each GTPase. GEF exchange activity was measured by the change in BODPY fluorescence (FAM channel). Fluorescence signals were collected at 30-s intervals for a total of 30 min using QuantStudio Real-Time PCR Software v1.2 on a QuantStudio 6 Flex instrument (Applied Biosystems). Raw fluorescence measurements were imported into Excel (Microsoft), and initial reaction rates were calculated from polynomial curves fitted to the data in XY scatter plots. The GEF activities of each protein were calculated using initial reaction rates.

### 3D protein modeling

Transcript ORFs were translated in APE. 3D protein structures were modeled using AlphaFold 3 (Abramson et al. 2024) on the AlphaFold Server (https://alphafoldserver.com). Generated models in Crystallographic Information File format were visualized and annotated using the web-based 3D structure viewer iCn3D (https://www.ncbi.nlm.nih.gov/Structure/icn3d).

### Statistical analyses

Data were imported into SPSS v29.0.1.0 (IBM) or GraphPad Prism (v10.3.1) for statistical analysis and graphing. Kolomogorov–Smirnov and Shapiro–Wilk tests of normality were performed using descriptive statistics functions. GLISA and neurite outgrowth data were analyzed using one-way ANOVA with Tukey’s honestly significant difference (HSD) post hoc test. SH-SY5Y morphology data were analyzed by independent-samples Kruskal–Wallis one-way ANOVA with a pairwise comparison of vectors. Significance values were adjusted by the Bonferroni correction for multiple tests. Adjusted significance values < 0.05 were considered significant. Dual GEF region protein dose–response curves for GEF exchange assays were analyzed using a nonlinear regression variable slope (four parameters) model.

### Data availability

The nanopore sequencing reads are available from ENA (PRJEB41557). Analysis pipelines, scripts, and isoform BED files are available via GitHub—haertyw/*KALRN*: Repository of the scripts used for the expression analysis of transcripts arising from *KALRN*.

## Results

### Characterization of full-length *KALRN* transcripts expressed in adult human brain and aorta

**Fig. 2:**
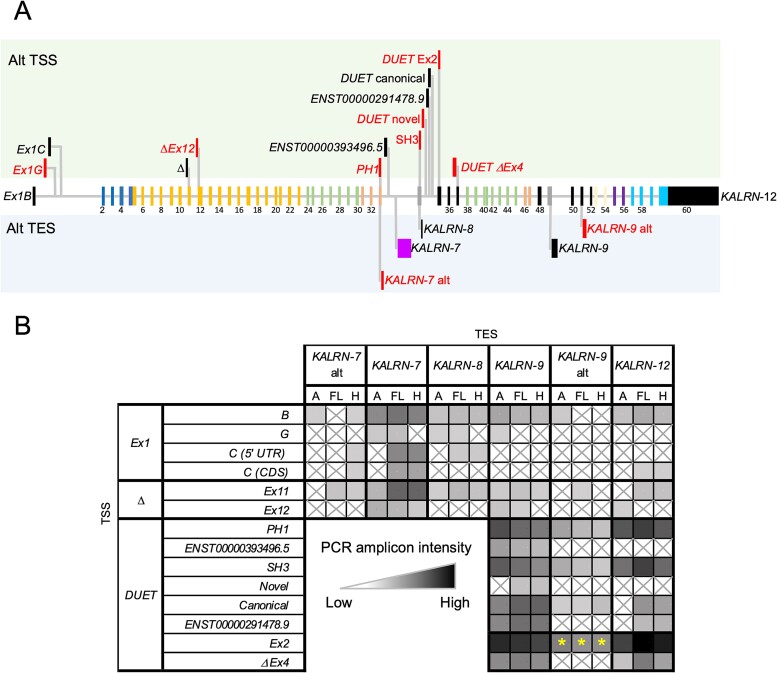
Full-length *KALRN* transcripts detected in adult human brain or aorta. A) *KALRN* transcript structures relative to the *KALRN* gene. Alternative TSS and TES in the brain and/or aorta are indicated relative to the exonic structure of the longest *KALRN* transcript, *KALRN*-12 (ENST00000360013.7). Novel sites identified by 5′ and 3′ RACE are highlighted in red. Exons are colored according to the protein domains that they encode, indicated on the Kalirin protein model in Fig. 1. B) PCR profiling of transcript start and end combinations revealed overlapping and tissue-specific *KALRN* isoform expression. Table shows the PCR amplification matrix with TSSs on the left (forward primers) and TESs (reverse primers) along the top. Relative amplicon abundance as determined by densitometry analysis of amplicon bands on ethidium bromide-stained gels is shown in gray scale. A = aorta, FL = frontal lobe and H = hippocampus, ⊠ = not detected. ^*^Two bands detected: major band abundance shown.

Short-read RNA-seq data (from the GTEx Project [https://www.gtexportal.org]) indicates that brain and arterial tissues are the primary sites of *KALRN* expression in adult human tissues (Fig. S1). Initially, we identified TSS and TES sites in two adult human brain regions (frontal lobe and hippocampus) and aorta. Rather than relying on potentially incomplete public annotations, we performed 5′ and 3’ RACE studies using polyA+ RNA pools derived from multiple individuals for each tissue (Fig. S3). Both annotated and novel TSS and TES were identified in at least one tissue type (Figs. 2A, S4, and S5). 5’ RACE identified a novel exon 1 TSS in the hippocampus, located between the human/rodent conserved 1B and 1C exons, that we have designated exon 1G, as an extension to the current nomenclature originally described by McPherson and colleagues (McPherson et al. 2002). A novel intragenic “Δ” TSS overlapping with exon 12 (ΔEx12) and multiple TSSs associated with the shorter DUET transcripts were also identified. For clarity, exon numbers throughout this manuscript are based on the GENCODE V44 top-ranked full-length *KALRN* transcript ENST00000682506.1.

5’-RACE also identified several TSSs that fall with exons shared with multiple transcripts. However, as these exons do not contain unique sequences, it is difficult to selectively amplify isoforms arising from these TSSs since PCR would also amplify fragments of longer transcripts that contain the same exon.

3’-RACE identified the canonical *KALRN-*7, -8, -9, and -12 TESs. In addition, two novel TESs were identified, an alternative upstream *KALRN-7* and an alternative downstream *KALRN-*9 termini. In both instances, the alternative TES was associated with an in-frame stop codon present in an extension to an annotated exon, i.e. exon 33 for *KALRN*-7 and exon 51 for *KALRN*-9 (Fig. S5). The *KALRN*-7 transcripts derived from the alternative TES is predicted to encode a Kalirin-7 protein that lacks a C-terminal PDZ binding domain, while the alternative *KALRN*-9 transcript is predicted to encode a Kalirin-9 protein with a 108 amino acid C-terminal extension (61 shared with Kalirin-12, data not shown).

We then assessed which TSS and TES combinations are utilized to generate full-length *KALRN* transcripts in the frontal lobe, hippocampus, and aorta by reverse-transcription PCR (RT-PCR). While some combinations were detected at similar levels in all tissues, others displayed tissue-specific or tissue-preferential expression (Fig. 2B). Full-length transcripts arising from the start exon 1G were detected in the aorta and frontal lobe. Shorter transcripts, particularly those arising from the various DUET TSSs displayed some of the highest amplicon intensities, although this may involve PCR amplification bias for shorter sequences.

**Fig. 3:**
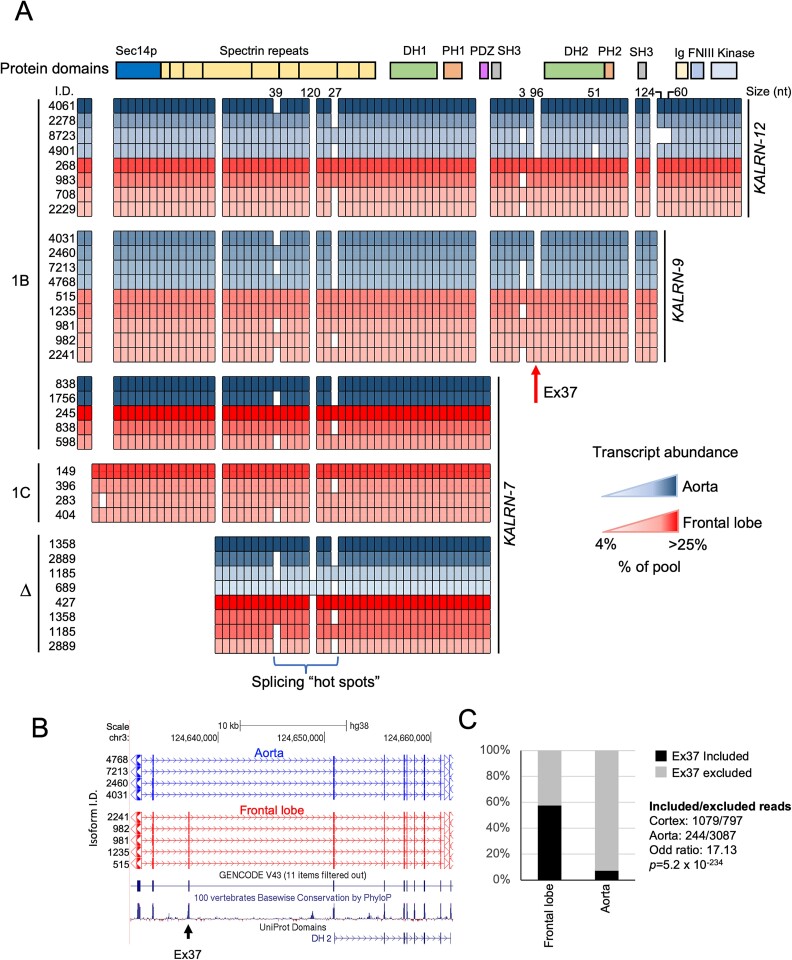
Long-read sequencing analysis of full-length *KALRN* transcripts identifies brain-specific splicing. A) Alignment of nanopore long-read sequences for transcripts contributing to >4% of the transcript pool for the most abundant TSS/TES combinations reveals brain-specific splicing leading to the inclusion of exon 37, encoding 32 amino acids upstream of the second GEF domain in longer *KALRN* isoforms. Hot spots of alternative splicing in the spectrin repeat coding regions were also observed with no apparent tissue specificity. Small boxes represent exonic parts (alternative splice donor/acceptor sites, not to scale) identified in the analysis of all transcripts. The nucleotide (nt) lengths of key exonic parts are indicated at the top. Unique IDs for each isoform are indicated on the left. Percent contribution of each splice isoform to individual TSS/TES amplicon pools is indicated by intensity gradient and colored by tissue. The regions coding the different protein domains (individually colored) are indicated at the top. B) A University of California, Santa Cruz (UCSC) browser (hg38) screenshot of mapped long read sequences for frontal lobe and aorta *KALRN-9* transcripts showing the location of the brain-enriched exon 37 relative to the *KALRN* gene C) Analysis of short-read GTEx splice junction data confirms preferential inclusion of this exon in adult human cortex vs aorta *KALRN* transcripts. Expression data for 42 paired tissues is shown.

All full-length *KALRN* PCR amplicons were then nanopore long-read–sequenced to identify splicing differences. To capture as many splicing events as possible, we included PCR amplicons arising from TSSs within shared exons, with the caveat that some of the sequences would be derived from longer overlapping transcripts. In total, 104 PCR amplicons (each consisting of a pool of potential splice variants) were sequenced including 37 from the frontal lobe, 36 from the hippocampus, and 31 from the aorta. Aligned sequencing reads for the top-ranked splice variants associated with the strongest *KALRN*-7, -9, and -12 PCR amplicons in the frontal lobe and aorta are presented in Fig. 3, while all splice variants contributing to >1% of these amplicon pools for frontal lobe, hippocampus, and aorta samples are presented in Fig. S6. Notably, all aorta *KALRN-*9 and *KALRN-*12 splice isoforms that individually contribute > 4% to each amplicon pool, skipped exon 37, which lies immediately upstream of the region that encodes the second GEF domain (Fig. 3A). The inverse was true for frontal lobe (Fig. 3B) and for the majority of hippocampal *KALRN*-9 and *KALRN*-12 transcripts (Fig. S6).

Analysis of GTEx short *read* RNA-seq splice junction data confirmed preferential inclusion and exclusion of exon 37 in brain and aorta *KALRN* transcripts, respectively (Fig. 3C). Comparison of GTEx median read counts for *KALRN* terminal exons across multiple tissues, indicates that *KALRN*-9 is the most abundant *KALRN* transcript in the aorta, while *KALRN*-7 is the most abundant in the brain (Fig. S7A). *KALRN*-9 terminal exon expression is detectable in the brain but at lower levels. *KALRN*-12 terminal exon levels are expressed at very low levels in this dataset. Assessing *KALRN*-9 (and *KALRN*-12) transcript abundance in short-read RNA-seq data using terminal exon expression is confounded by the contribution of DUET transcripts to the signal.

To estimate the percent contribution of DUET transcripts to *KALRN*-9/*KALRN*-12 terminal exon counts in GTEx data, we subtracted per base normalized read counts for exon 45 (present in *KALRN*-9 and *KALRN*-12 but not DUET transcripts, based on GTEx isoform predictions) from per base normalized read counts for the DUET/*KALRN*-9/*KALRN*-12 shared exon 48, and then divided this number by the shared exon counts and multiplied by 100. Results indicated that DUET transcripts contribute a substantial percentage of the *KALRN*-9 terminal exon signal in the brain (~66% in the frontal lobe) but less so in the aorta (~25%). In skeletal muscle, the majority (~85%) of the *KALRN*-9 terminal exon signal can be attributed to DUET (Fig. S7B).

To estimate levels of *KALRN*-9 transcripts containing exon 37 in different tissues, we multiplied the estimated level of *KALRN*-9 by the ratio of exon 37 “spliced-in” vs “spliced out” (calculated using GTEx splice junction read counts). Based on these data, *KALRN*-9 transcripts containing exon 37 are present in a subset of tissues, albeit at relatively low levels (Fig. S7C). In addition to the brain and aorta, they are detected in skeletal muscle and heart. In the brain, heart, and skeletal muscle, a significant proportion of *KALRN*-9 transcripts contain exon 37, while in the aorta, exon 37 is skipped in most *KALRN*-9 transcripts. In addition to exon 37 skipping, we identified splicing “hot spots” within the spectrin repeat coding regions that alter the amino acid number and, potentially, 3D protein structure (Figs. 3A and S8). These splicing events didn’t display any obvious tissue preference (Fig. 3A). Splice isoforms for abundant DUET amplicons were similar between the different tissues (not shown).

### Functional studies of the top frontal lobe and aorta proteoforms

**Fig. 4:**
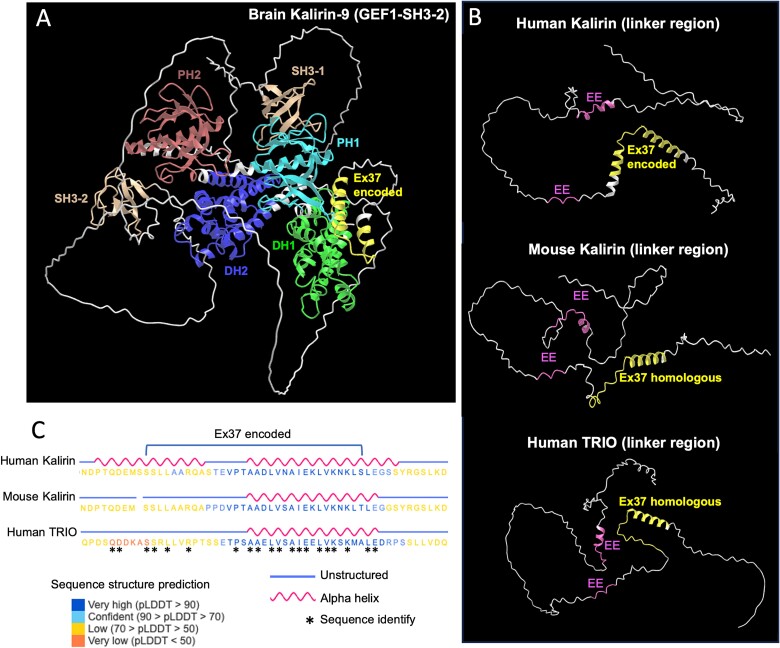
*KALRN* exon 37 is predicted to encode an alpha helix that is potentially conserved between species and paralogous protein, TRIO. A) AlphaFold 3 structural model of the GEF1 to SH3-2 region of the predicted protein for the most abundant brain *KALRN*-9 isoform. The different protein domains and the 32 amino acids encoded by exon 37 (Ex37) are highlighted by different colors. B) The 32 amino acids encode an alpha helix within a GEF linker region that contains previously identified GEF1 activity enhancer elements (EEs). Homologous alpha helices and enhancer elements are present in mouse Kalirin (UniProt I.D. A2CG49) and human TRIO (UniProt I.D. O75962-1) proteins. C) Protein sequence alignments of human and mouse Kalirin and human TRIO helix regions.

The proteins encoded by the most abundant brain and aorta *KALRN* isoforms from Fig. 3 were then predicted (data not shown). Structural modeling (AlphaFold3) of the central region the brain Kalirin-9 protein (identical to the same region of the brain Kalirin-12 proteoform) indicates that the 32 amino acids encoded by exon 37 forms an alpha helix, indicative of potential function (Fig. 4). Notably, this alpha helix is located within a GEF linker region that contains highly conserved enhancer elements that regulate GEF1 activity (Bandekar et al. 2022).

**Fig. 5:**
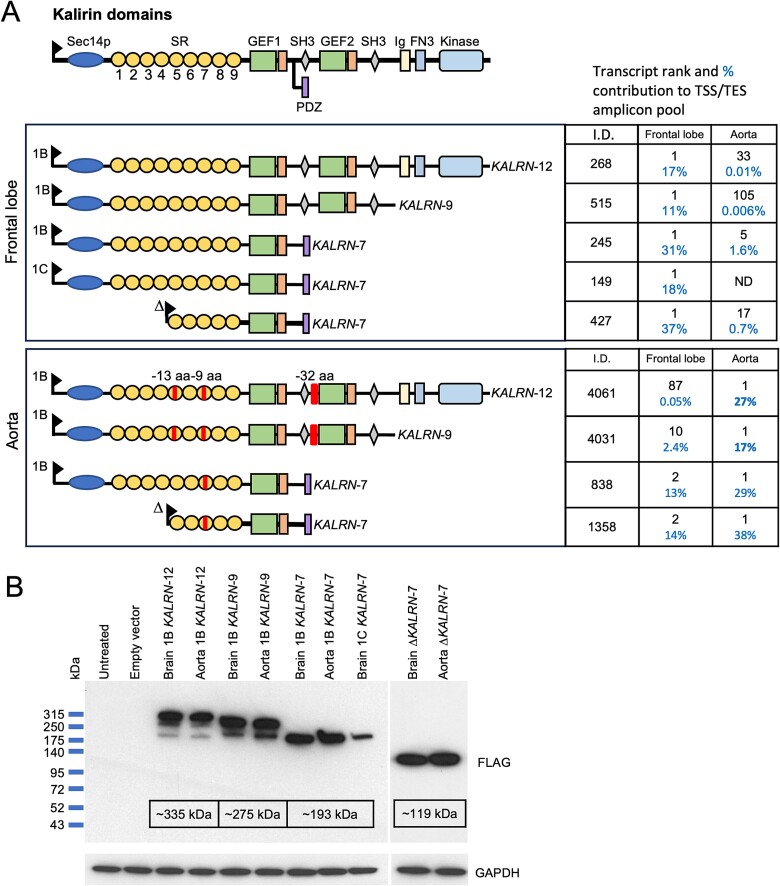
Expression constructs for the most abundant brain and aorta *KALRN* isoforms. A) Protein domains encoded by the most abundant frontal lobe and aorta *KALRN* isoforms from each of the major transcript amplicon pools chosen for functional analysis. Transcript rank and percentage contribution to the amplicon pool (individual transcript start/end combination) in each tissue are indicated. 1C-*KALRN-*7 is selectively expressed in the brain. Red boxes mark the deletion regions present in the different aorta proteoforms due to alternative transcript splicing. B) Western blot analysis of RIPA lysates of HEK293T cells 48 h post-transient transfection with plasmids encoding different N-terminal FLAG-tagged Kalirin proteoforms. Major bands for each proteoform were detected at the expected size. GAPDH was probed to confirm equal protein loading.

Mammalian expression plasmids for the most abundant frontal lobe and aorta *KALRN* isoforms were synthesized to assess the functions of the encoded proteins. An N-terminal FLAG tag was added for protein detection. Transcript rankings within each PCR amplicon pool and the protein domains that each isoform encodes are presented in Fig. 5A. Western blot analysis confirmed that each plasmid expressed a FLAG-tagged protein of the predicted size (Fig. 5B). Kalirin-9 and Kalirin-12 proteins also displayed FLAG-positive bands of lower molecular weight, indicating that a small amount of each protein is cleaved. The size of the bands indicates that the proteins are cleaved at sites that lie between the two GEF domains.

Arguably, the primary catalytic function of Kalirin is to activate RhoGTPases via its GEF domains. The GEF1 domain binds to and activates RAC1 and RHOG, while the GEF2 domain binds to and activates RHOA (Penzes et al. 2001; May et al. 2002).

**Fig. 6:**
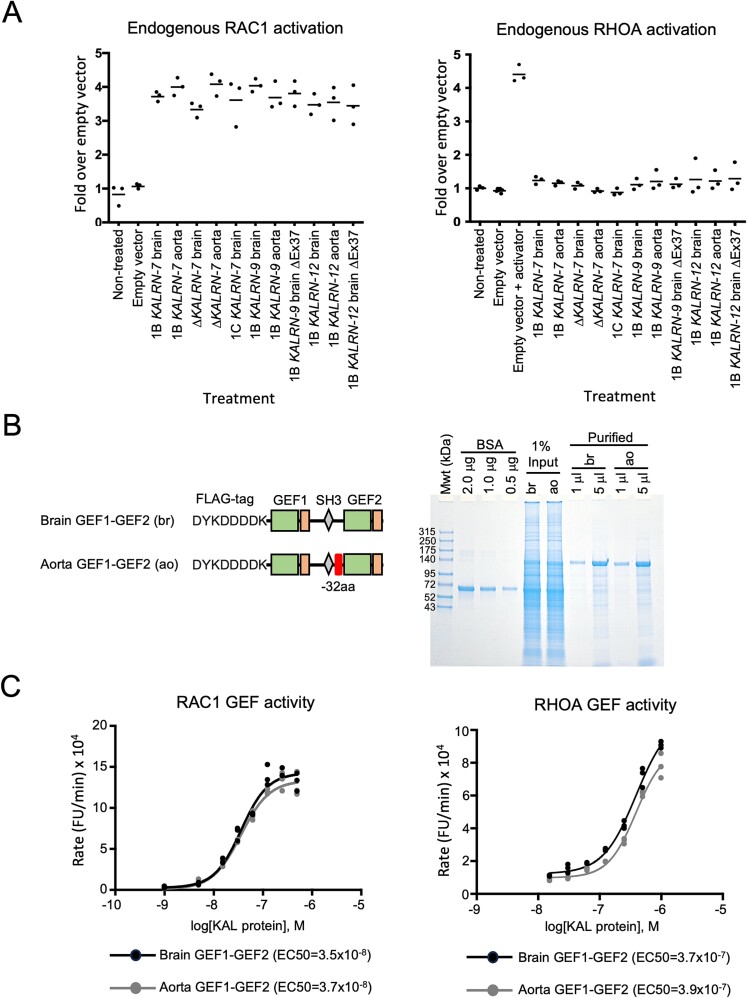
Brain and aorta Kalirin proteoforms display similar GEF activities. A) All Kalirin proteoforms significantly (Tukey’s HSD < 0.001 vs empty vector) activated endogenous RAC1 in serum-starved HEK293T cells in transient transfection studies. Empty vector transfection had no effect in comparison to nontreated controls. Levels of RAC1 activation were not significantly different between the different *KALRN* vector-transfected groups. In contrast, none of the *KALRN* vectors significantly activated endogenous RHOA. Treatment of empty vector transfections with an RHOA activator confirmed that the cells were responsive (Tukey’s HSD < 0.0001 vs empty vector). B) Recombinant brain and aorta dual GEF domain proteins. FLAG-tagged truncated dual GEF Kalirin proteins were immunopurified from transiently transfected HEK293T cell lysates. Protein purity was assessed by SDS-PAGE and Coomassie staining. C) Catalytic activities of the purified Kalirin dual GEF proteins (KAL) were assessed by RAC1 and RHOA GEF exchange assays. EC50s were similar for brain and aorta protein variants within each assay (*P* = 0.69 and 0.8696 for RAC1 and RHOA GEF activities, respectively). RHOA GEF activity was approximately 10-fold lower than RAC1 GEF activity for both proteins (EC50 3.7 × 10^−7^ vs 3.5 × 10^−8^ for brain dual GEF proteins in RHOA vs RAC1 GEF exchange assays, respectively).

Using transient transfection studies, we examined the effects of exogenous expression of the different Kalirin proteoforms on the activation of endogenous RAC1 and RHOA in serum-starved HEK293T cells. Two additional expression vectors containing brain *KALRN*-9 and brain *KALRN*-12 isoforms, each with exon 37 deleted, were cloned and included in the analysis. Exogenous Kalirin protein expression in each sample was confirmed by Western blot (Fig. S9). Levels of activated RAC1 in cell lysates were robustly and significantly increased by exogenous Kalirin expression at 24 h post-transfection when compared to empty vector-transfected or nontreated controls (Fig. 6A). The levels of activated RAC1 were similar between the different *KALRN* vector–transfected groups suggesting that neither the amino acid deletions present in splice isoforms, nor the downstream domains selectively present in longer proteoforms, had any major influence on GEF1 activity in this cell line. Active RHOA levels were not significantly altered by exogenous expression of any of the Kalirin proteoforms (Figs. 6A, S9, and S10).

To directly measure GEF activity and investigate why dual GEF Kalirin proteoforms failed to significantly activate RHOA in transfection experiments, we cloned, expressed, and affinity-purified the dual GEF coding regions of the brain and aorta *KALRN*-9 isoforms and measured their RAC1 and RHOA GEF activity using GEF exchange assays (Fig. 6B and C). The RHOA GEF activity of both aorta and brain-derived proteins was approximately 10-fold lower than their RAC1 GEF activity (Fig. 6C). Thus, the GEF activity of the GEF2 containing Kalirin proteoforms may have been insufficient to activate endogenous RHOA in the transient transfection studies. Although the brain and aorta GEF region proteins differ by 32 amino acids in the linker region between the two GEF domains, the activities of the individual GEF domains were similar between the two proteins.

A *KALRN* missense mutation (rs143835330), that leads to a proline to threonine amino acid change downstream of the GEF2 domain enhances RHOA activity during states of activation (Grubisha et al. 2021). Thus, to determine if aorta and brain dual GEF region proteins function differently under states of RHOA GEF activation, we deleted the regulatory PH2 domain (Fig. S10). As predicted, deletion of the PH2 domain significantly increased RHOA activation in transfection studies; however, additional deletion of exon 37 was without effect. Thus, we conclude that the 32 amino acids encoded by exon 37 do not directly autoregulate either GEF1 or GEF2 activity.

We then examined the effects of exogenous expression of aorta and brain Kalirin-7 and Kalirin-9 proteoforms on neurite outgrowth using SH-SY5Y cells. These cells display an immature neuronal phenotype with few, short processes but have the potential to transform into a more mature neuron-like phenotype with elaborate neurite arborization when stimulated (Hromadkova et al. 2020) Transfection of undifferentiated SH-SY5Y cells with each Kalirin expression vector stimulated overt morphological changes within 12 h of transfection including lamellipodia formation and cell spreading compared to control vector–treated cells (Fig. S11A and B). Each proteoform was present in the cytoplasm and in the tips of outgrowing neurites (Fig. S11A). To quantify cell spreading, we performed morphometric analysis of 80 to 218 cells per group using CellProfiler (Fig. S11C). All Kalirin proteoforms tested significantly increased cell area relative to empty vector controls, without any clear difference in their effects between proteoforms.

**Fig. 7:**
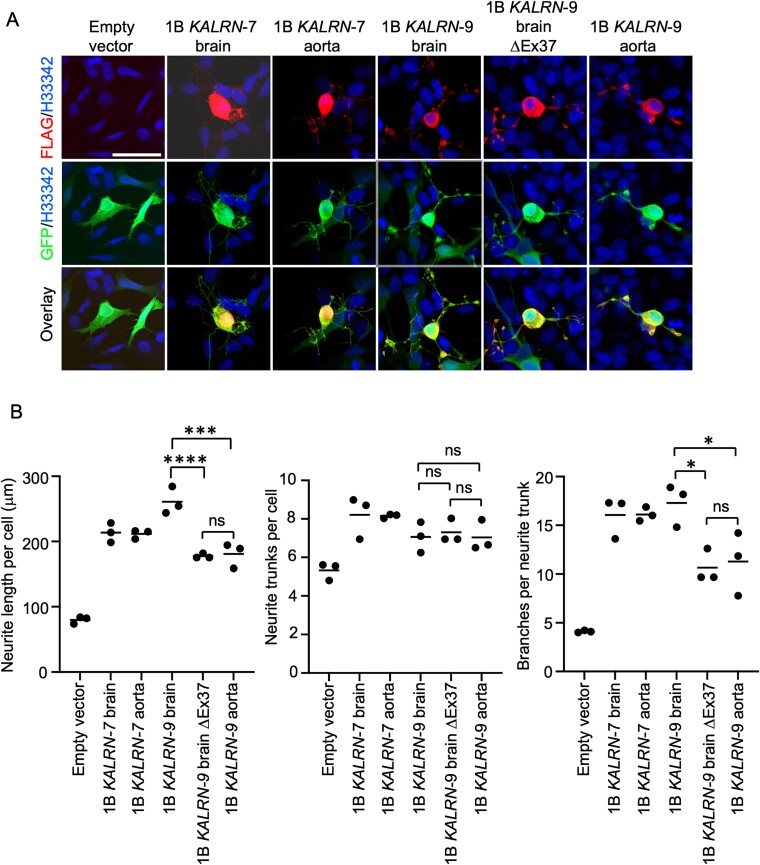
Effects of brain and aorta Kalirin-7 and Kalirin-9 proteoforms on neurite outgrowth in SH-SY5Y cells 48 h after transfection. A) Confocal immunofluorescence microscopy analysis of FLAG-tagged Kalirin protein expression. Each of the Kalirin proteoforms was detected throughout the soma and outgrowing neurites. All groups were cotransfected with a plasmid that expresses a cytoplasmic EGFP, to assist with cell visualization. Scale bar = 50 μm. B) Morphometric analysis of neurite outgrowth. All *KALRN* vectors stimulated neurite outgrowth, with Kalirin-9 proteoforms displaying different potencies. Brain *KALRN*-9ΔEx37 and aorta *KALRN*-9-transfected cells displayed reduced neurite outgrowth (length and branching) compared to those transfected with brain *KALRN*-9. Triplicate transfections per vector were analyzed, and bars indicate the group mean. Individual data points show mean values of >50 cells analyzed per transfection. Differences between groups were assessed using Tukey’s HSD. Adj. *P* < 0.05 was considered significant. Relative to empty vector controls, all Kalirin isoforms significantly increased total neurite length per cell (*P* < 0.0001) and branches per neurite trunk (*P* < 0.05). ^*^P-value < 0.05, ^***^P-value ≤ 0.001, ^****^P-value ≤ 0.0001, and ns = nonsignificant.

At 48 h post-transfection, each of the *KALRN* vector transfected groups displayed elaborate neurite outgrowth with significant increases in total neurite length per cell and the number of branches per neurite, when compared to empty vector controls (Figs. 7A and B and S12). Further, the different Kalirin-9 proteoforms displayed different potencies. Notably, cells transfected with vectors expressing Kalirin-9 proteoforms lacking the 32 amino acids encoded by exon 37 (i.e. brain *KALRN*-9ΔEx37 and aorta *KALRN*-9 groups) displayed blunted neurite outgrowth when compared to cells expressing Kalirin-9 protein containing these 32 amino acids (i.e. brain *KALRN*-9). Differentiated SH-SY5Y cells were resistant to transfection, so it was not possible to examine the effects of exogenous expressed Kalirin proteoforms in this cell population.

## Discussion

### Adult human brain and aorta express a diverse array of KALRN transcripts, some with tissue preferential expression

We performed an in-depth characterization of adult human brain and aorta expressed *KALRN* transcripts using RACE and PCR-targeted long-read sequencing. We identified novel TSS and TES and a diverse array of splice isoforms confirming that, like other genes in the brain (Clark et al. 2020), current annotations underestimate *KALRN* transcript diversity. Further, our studies identify tissue preference for TSS usage. The 1C TSS was detected in the brain but not the aorta, suggesting that the proteins coded by transcripts arising from this TSS may be particularly important for brain function. In the rodent brain, expression of 1C *KALRN*-7 transcripts coincides with synapse formation (Miller et al. 2017b). Notably, the unique N-terminus region of proteins derived from 1C transcripts has been shown to influence cellular localization, phosphoinositide binding, and potential function (Miller et al. 2015).

We detected abundant expression of the short GEF2 encoding DUET transcripts, originally considered a skeletal muscle–specific transcript (Kawai et al. 1999). It is unknown how much DUET protein is expressed in the adult human brain since most studies analyzing Kalirin expression have used antibodies raised against regions of protein that are absent in the shorter DUET proteoforms. Notably, DUET transcript structures were similar between tissues, so we focused our attention on other *KALRN* transcripts that displayed tissue-specific expression profiles.

Long-read sequencing revealed an abundance of splice variation within longer *KALRN* transcripts. Splicing hotspots were observed in spectrin repeat encoding regions 5 and 7, resulting in 13 and 9 amino acid deletions, respectively. The 13 amino acids in spectrin repeat 5 contain multiple serine residues, some of which display isoform-specific phosphorylation (Miller et al. 2017a). Some of these amino acids are in the hinged region between spectrin repeats 4 and 5, previously predicted to be involved in cofactor binding (Vishwanatha et al. 2012). The 13 amino acid sequence also contains a calpain cleavage site predicted to regulate protein activity by cleaving the N-terminal domain (Miller et al. 2017a); however, we did not see evidence of cleavage at this site in any of the exogenous Kalirin proteins in transfected HEK293T lysates. Notably, Western blot analysis indicates that a small amount of each exogenous Kalirin-9 and Kalirin-12 proteoform is proteolytically cleaved at other sites between the two GEF domains. The protein encoded by the most abundant aorta *KALRN-9* transcript, included in functional analyses in this study, contained both spectrin repeat deletions. In contrast, the brain Kalirin-9 protein (encoded by the most abundant brain *KALRN-9* transcript) did not contain either deletion. Both aorta and brain Kalirin-9 proteoforms equivalently activated endogenous RAC1 when exogenously expressed in HEK293T cells, suggesting that neither of these amino acid deletions in the spectrin repeat domains has any major effect on GEF1 activity.

Sequence variation in the GEF domains was restricted to low-abundance transcripts, suggesting that the GEF function is highly conserved and requires tight regulation. However, a major splicing difference between the brain and the aorta was identified between the two GEF domain-encoding regions, which leads to exon 37 skipping in abundant aorta *KALRN*-9 and *KALRN*-12 transcripts. Skipping of the orthologous exon in rodent *Kalrn*-9 transcripts has also been reported (Johnson et al. 2000). Skipping of exon 37 results in an in-frame deletion of 32 amino acids of unknown function immediately upstream of the second GEF domain. Notably, increased skipping of this exon has been observed in *KALRN* transcripts in the brains of individuals with schizophrenia and autism spectrum disorder, suggesting that this event is of potential clinical significance (Gandal et al. 2018).

### The 32 amino acids encoded by exon 37 have no detectable effect on GEF1 or GEF2 activity

While the protein structure of full-length Kalirin has not been definitively determined, CryoEM analysis of Trio, a paralogue of Kalirin, reveals that the central region of the protein encompassing both TrioN (GEF1) and TrioC (GEF2) domains forms a condensed structure, while the spectrin repeats have an extended configuration (Bandekar et al. 2022). This is consistent with AlphaFold 3-predicted protein models for both TRIO and Kalirin. The condensed nature of the central part of TRIO and Kalirin proteins suggests that the region linking the two GEF domains is involved in the intra-molecular regulation of GEF activity. Accordingly, the same study identified a disordered region between the TrioN and TrioC domains that selectively enhances TrioN Rac1 GEF activity. High sequence conservation suggests an equivalent regulatory domain exists in dual GEF Kalirin proteoforms (Bandekar et al. 2022).

The 32 amino acids encoded by exon 37 lie within the GEF linker region that contains conserved GEF1 activity enhancer elements (Bandekar et al. 2022). Structural modeling using AlphaFold 3 reveals that a subset of these 32 amino acids forms an alpha helix that, like the enhancer elements, appears to be conserved between species and paralogues. While the proximity of this alpha helix to the conserved GEF enhancer elements suggests it could function to regulate GEF activity, we saw no evidence of this as assessed by measuring endogenous RAC1 activation in transfection studies and RAC1 GTP loading in GEF exchange assays. One caveat is that Kalirin GEF1 also binds and activates RhoG (May et al. 2002), but effects on this RhoGTPase were not assessed in this study.

Overexpression of dual GEF Kalirin proteoforms in HEK293T cells robustly activated endogenous RAC1 but failed to significantly activate RHOA. Previous studies in primary cortical rat neurons also failed to show any effect of overexpression of wild-type Kalirin-9 on RHOA activation (Grubisha et al. 2021). Further, heterozygous deletion of *KALRN* reduces Kalirin-9 levels, and RAC1 but not RHOA activation, in smooth muscle cells (Wu et al. 2013). This suggests that the RHOA GEF activity of dual GEF Kalirin proteins is relatively weak at resting state. In support of this, we observed a 10-fold lower GEF activity for RHOA vs RAC1 for purified truncated dual GEF proteins in GEF exchange assays. Weak RHOA GEF activity may involve auto-inhibition of the DH2 domain by the PH2 domain. G protein subunit alpha q (Gαq) has been shown to bind the PH2 domains of both Trio and Kalirin, effectively blocking PH2 interaction with DH2 and its inhibitory effects on DH2 activity (Lutz et al. 2007). Consistent with this hypothesis, deletion of the PH2 domain from Kalirin dual GEF region proteins (GEF1-GEF2ΔPH2) increased RhoA activation in our transfection studies. Thus, full GEF2 activity may require the presence of a cofactor. Additional deletion of the 32 amino acids encoded by exon 37 had no effect on RhoA activation. Further, purified brain and aorta dual GEF region proteins displayed similar RHOA GEF activity in GEF exchange assays. This suggests that the 32 amino acids encoded by exon 37 have little effect on GEF2 function, at rest or during states of activation, at least in the absence of yet unidentified cofactors.

### The 32 amino acids encoded by exon 37 alter neurite outgrowth in SH-SY5Y cells

Exogenous expression of Kalirin had vastly different effects on the morphology of the neuronal-like SH-SY5Y cells compared to HEK293T cells. In HEK293T cells, overexpression of full-length Kalirin-7 or Kalirin-9 proteoforms stimulated cell rounding [previously examined in detail by Schiller and colleagues (Schiller et al. 2008)], while in SH-SY5Y cells, the same Kalirin proteoforms stimulated cell flattening, differentiation, and neurite outgrowth. The cell type–specific effects of exogenous Kalirin expression on cellular morphology may be due to different levels of regulatory factors. For example, Gαq transcripts are 4-fold higher in SH-SY5Y cells compared to HEK293 cells in the human protein atlas (https://www.proteinatlas.org), raising the possibility that increased levels of Gαq in SH-SY5Y cells relieve auto-inhibitory effects of the PH2 domain on RHOA GEF activity. Unfortunately, the lower transfection efficiency of SH-SHY5Y compared to HEK293T cells makes it difficult to assess the effects of Kalirin protein overexpression on the global levels of RHOA activation in these cultures. Notably, Kalirin proteins display overlapping and proteoform-specific effects on SH-SY5Y cell morphology. All tested Kalirin proteoforms initially stimulated cell flattening; however, aorta Kalirin-9 and brain Kalirin-9ΔEx37 proteoforms subsequently displayed reduced neurite outgrowth activity compared to brain Kalirin-9 indicative that the region encoded by exon 37 is functional in neurons.

### Potential functions of the brain-enriched alpha helix

It is possible that the 32 amino acids encoded by exon 37 mediate interactions with key regulatory factors. Such interactions would be dependent on the abundance of the binding partner and so are potentially cell type or developmental stage specific. Previous studies demonstrate that the region between the two GEF domains of rodent Kalirin-9, which includes the homologous region encoded by exon 37 in humans, interacts with p75 to regulate axon growth via Nogo receptor-mediated activation of RHOA in cerebellar granule neurons (Harrington et al. 2008). In this context, Kalirin-9 competes with Rho GDP-dissociation inhibitor (RhoGDI) for binding to p75. Notably, the effects of knocking down Kalirin-9 on axon growth were only observed in the presence of the appropriate signaling cue, suggesting that this and other binding factor interactions are context specific.

As with almost all studies of this kind, it is not possible with available antibodies to distinguish between many of the isoforms to prove their existence at a protein level in human tissue samples. This includes the isoforms that display the 32 amino acid differences due to the skipping of exon 37 that we have focused on in this study. However, given the preferential inclusion of this exon in dual GEF *KALRN* transcripts in the brain and reports that splicing out of this exon is increased in schizophrenia and autism spectrum disorder, further characterization of its potential function in an appropriate and physiologically relevant biological context is warranted.

## Supplementary Material

Supp_figures_for_manuscript_bhae470
