# Supplementary material for: Identification and characterization of human KALRN mRNA and Kalirin protein isoforms
Source: Cereb Cortex. 2024 Dec 4;34(12):bhae470. doi: 10.1093/cercor/bhae470 (PMC11630257; doi:10.1093/cercor/bhae470)
Supplement: Supp_figures_for_manuscript_bhae470 [file supp_figures_for_manuscript_bhae470.pdf]

Primer	Sequence	Strand	Genomic coordinates (hg38)
**5'RACE**			
Ex5 RT/outer 2	TTGTAGTCCAGGGAGCCATC	-	chr3:124268809-124268828
Ex4 outer/nested 2	TTGAGGAGGGGCTTGATGAG	-	chr3:124264559-124264578
Ex3 nested	CTGGCCAAATACGTCACGAG	-	chr3:124234915-124234934
Ex32 RT	GTTTTGGGGAGCTGAAGTGG	-	chr3:124492833-124492852
Ex12 outer 1	CTTCGCCTTCCTTGATGACA	-	chr3:124395266-124395285
Ex12 nested 1	GCATCTAGAGTGGCGGTCT	-	chr3:124395238-124395256
Ex39 RT	CCACAATGCCCAGATCCTTG	-	chr3:124655643-124655662
Ex38 outer	CTGCAGGGTCTTTCAAGCTC	-	chr3:124650834-124650853
Ex38 nested	CCCGGTATGAGCTTCCTTCTAG	-	chr3:124650811-124650832
Ex36 outer	CACCCCAACCTTTCTTGCTC	-	chr3:124633851-124633870
Ex36 nested	AGGGGTGTGTGTGACTCTTC	-	chr3:124633885-124633904
**3'RACE**			
Ex23 outer	ATCGGGAGGTCAAGCTGC	+	chr3:124456656-124456673
Ex33 nested	AGCCAACCAGACACCATCTC	+	chr3:124496351-124496370
Ex38 outer	CCCACCCACACCTCCTAAAA	+	chr3:124650876-124650895
Ex48 nested	GGCAGTCCAGGGTTTGAATA	+	chr3:124671845-124671864
Ex58 outer	TGCATTCACAAAGCTACCCG	+	chr3:124712960-124712979
Ex59 nested	GGCCGGCTCTTAGACTACCT	+	chr3:124717257-124717276

Table S1. PCR primers used for 5' and 3' RACE. See methods section for details of annealing temperatures used for PCR.

Primer	Sequence	Strand	Genomic coordinates (hg38)
Ex1C TSS-1	tttctgttggtgctgatattgcAAGGATGAGTTCAGGGTGGG	+	chr3:124094818-124094837
KALRN-12 TES	acttgcctgtcgctctatcttcCTACGTCCCTTGGTTCACCCGGTTG	-	chr3:124719446-124719470
Ex1B TSS	tttctgttggtgctgatattgcCCCTCCCACAGTCATGAACC	+	chr3:124033728-124033747
Ex1C TSS-2	tttctgttggtgctgatattgcGGTATCTCCGCTTGCTCCG	+	chr3:124094872-124094890
Ex1G TSS	tttctgttggtgctgatattgcGAAGCTGAGGCACATGGAG	+	chr3:124094108-124094126
∆Ex11 TSS	tttctgttggtgctgatattgcACTTTGCCTCACTGTTGTTGC	+	chr3:124384818-124384838
∆Ex12 TSS	tttctgttggtgctgatattgcTCTCCCATCTTCCCTGAGTG	+	chr3:124395092-124395111
KALRN-7 alt TES	acttgcctgtcgctctatcttcTCCAGCCCTGTTCTTTCCTC	-	chr3:124496549-124496568
KALRN-7 TES	acttgcctgtcgctctatcttcGAACATGTTGCCCTCTGAGC	-	chr3:124518593-124518612
KALRN-8 TES	acttgcctgtcgctctatcttcACACAGTTACTCGGGAGCTC	-	chr3:124563260-124563279
KALRN-9 TES	acttgcctgtcgctctatcttcTCCTCATACTCCTCTCCCGG	-	chr3:124674636-124674655
KALRN-9 alt TES	acttgcctgtcgctctatcttcCTTTTATAGCTGGGATGGGATACC	-	chr3:124679641-124679664
DUET PH1 TSS	tttctgttggtgctgatattgcGCACCCCATCCTCAGACAAT	+	chr3:124491390-124491409
DUET SH3 TSS	tttctgttggtgctgatattgcATCTCACACTCCCGAAGCAG	+	chr3:124563035-124563054
DUET Novel TSS	tttctgttggtgctgatattgcCTCCCCGCTTCCCAAGGT	+	chr3:124584637-124584654
DUET canonical TSS	tttctgttggtgctgatattgcCTCTTTGGGGTGGCTCTTTG	+	chr3:124584857-124584876
DUET Ex2 TSS	tttctgttggtgctgatattgcAGCTTTGACCTGGGATCTCC	+	chr3:124632638-124632657
DUET Ex4 TSS	tttctgttggtgctgatattgcCCCACACTCTTCTCCCTCTG	+	chr3:124637054-124637073
ENST00000393496_5 TSS	tttctgttggtgctgatattgcAAGCAAATCTGAGAGCAAGCC	+	chr3:124504879-124504899
ENST00000291478_9 TSS	tttctgttggtgctgatattgcTCCCAGTAAGTCAGAGCTGC	+	chr3:124584664-124584683

Table S2. PCR primers for *KALRN* targeted nanopore long-read sequencing. Gene-sequence primer and adaptor sequences are indicated in upper and lower case, respectively. See methods section for details of annealing temperatures used for PCR.

Antibody	Clone	Supplier	Cat. number	Batch number	Method	Working conc.
Anti-FLAG	M2	Merck	F1804	SLCM4081	Western blot	500 ng/ml
					Immunofluorescence	1 μg/ml
Anti-GFP	-	Abcam	ab6556	GR3351352	Immunofluorescence	250 ng/ml
GAPDH	14C10	Cell Signaling Technology	2118	14	Western blot	1 ng/ml
Anti-mouse ECL	-	Cytiva	NA931	17434166	Western blot	50 ng/ml
Anti-rabbit ECL	-	Cytiva	NA934	17824033	Western blot	25 ng/ml
Anti-mouse Alexa Fluor 594	-	Invitrogen	A-21206	2352146	Immunofluorescence	5 μg/ml
Anti-rabbit Alexa Fluor 488	-	Invitrogen	A-21203	2376850	Immunofluorescence	5 μg/ml

Table S3. Antibodies used in this manuscript

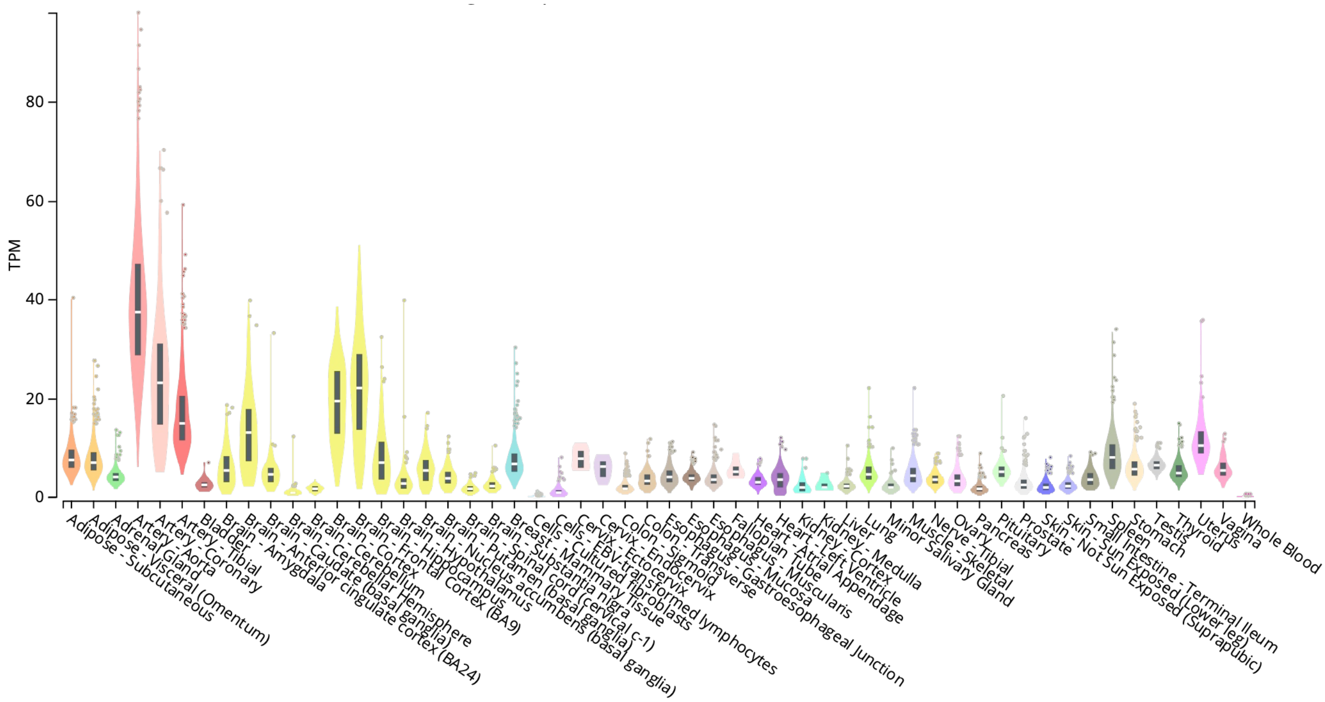

Figure S1- Bulk tissue gene expression for *KALRN* (ENSG00000160145.15) obtained from the The GTEx Portal (GTEx Analysis Release V8, dbGaP Accession phs000424.v8.p2) on 21/12/23.

Figure S2. KALRN isoform coding sequences(CDS) used for expression vectors.

>Isoform 268

ATGAACCCCCCTGAGGGAGCAGCGGAGGAAGGAGGAGCAGCAGACTCGGACGTGGACGCCTTTTTCCGGACAGGGTCTTTTCGGAATGATGGTTTGAAAGCTT
CTGATGTCCTTCCTATCCTAAAGGAAAAGGTGGCCTTCGTGTCTGGGGGTCGTGATAAGCGAGGCGGACCCATCCTGACCTTCCCTGCTCGCAGCAATCATGACAG
AATAAGACAGGAAGACCTGCGGAAACTCGTGACGTATTTGGCCAGCGTGCCAAGTGAGGACGTGTGCAAACGTGGCTTCACTGTCATCATCGACATGCGGGGCT
CCAAGTGGGACCTCATCAAGCCCCTCCTCAAAACGCTGCAGGAAGCCTTTCCAGCTGAGATCCATGTGGCCCTCATCATTAAACCCGACAACTTCTGGCAGAAACA
GAAGACCAACTTTGGCAGCTCCAAATTCATCTTTGAGACGAGCATGGTATCTGTGGAGGGCCTCACAAAGCTGGTGGACCCCTCCCAGCTGACGGAGGAGTTTG
ATGGCTCCCTGGACTACAACCATGAGGAGTGGATCGAACTGCGGCTCTCCCTGGAGGAGTTCTTCAACAGCGCCGTGCACCTGCTCTCGCGCCTCGAGGACCTCC
AGGAGATGCTAGCCCGGAAGGAGTTTCCTGTGGATGTGGAGGGCTCTCGGCGGCTCATTGACGAACACACACAGCTCAAGAAAAAGGTGCTGAAGGCCCCTGT
GGAGGAGCTGGACCGGGAGGGGCAGCGGCTGCTGCAGTGCATCCGCTGCAGCGACGGCTTCTCAGGACGCAACTGCATCCCGGGCAGTGCTGACTTCCAGAG
CCTGGTGCCCAAGATCACCAGTCTCCTGGACAAGCTGCACTCCACCCGGCAGCACCTGCACCAGATGTGGCATGTGCGCAAGCTCAAGCTGGACCAGTGCTTTCA
GCTGCGGCTCTTCGAGCAGGATGCTGAGAAGATGTTTGACTGGATAAGCCACAACAAGGAGTTATTCCTCCAGAGCCACACGGAGATCGGAGTCAGCTACCAGT
ACGCCCTTGACCTCCAGACGCAGCACAATCACTTTGCCATGAACTCCATGAATGCCTATGTCAACATCAACCGCATCATGTCCGTGGCTTCCCGCCTCTCTGAGGCC
GGTCATTATGCCTCACAACAAATCAAGCAGATCTCCACCCAGCTGGACCAGGAGTGGAAGAGCTTCGCTGCTGCCCTGGATGAACGCAGCACCATCCTCGCCATG
TCTGCTGTGTTCCACCAGAAGGCTGAGCAGTTCCTGTCGGGAGTGGATGCCTGGTGCAAGATGTGCAGTGAAGGTGGTCTGCCATCCGAGATGCAAGACCTAGA
GCTGGCAATCCACCACCACCAGACCTTGTATGAGCAGGTGACCCAAGCCTACACAGAGGTCAGCCAGGATGGCAAAGCACTACTTGATGTGCTGCAGCGGCCCC
TGAGCCCTGGGAACTCCGAATCCCTCACGGCCACAGCCAACTACTCCAAGGCAGTGCACCAGGTGCTGGACGTGGTGCATGAGGTGTTACATCACCAGCGACGG
CTGGAGAGCATCTGGCAGCACCGCAAGGTGCGGCTCCACCAGCGGCTGCAGCTCTGCGTCTTCCAGCAGGATGTACAGCAGGTGTTGGACTGGATTGAAAACCA
TGGTGAGGCCTTTCTCAGCAAACACACTGGAGTTGGGAAGTCCCTACATCGAGCCCGGGCCCTGCAGAAGAGGCATGATGACTTTGAAGAGGTGGCTCAGAATA
CGTACACCAATGCGGACAAGCTCCTAGAAGCAGCAGAGCAGTTGGCTCAGACGGGGGAATGTGACCCCGAGGAGATCTACAAGGCAGCTCGACACCTGGAGGT
GCGCATCCAAGACTTCGTGCGCAGGGTGGAGCAGCGGAAGCTTCTCCTGGACATGTCTGTTTCCTTCCACACACACACCAAAGAGTTGTGGACATGGATGGAAG
ACCTTCAGAAGGAGATGTTGGAGGATGTCTGTGCAGATTCTGTGGATGCAGTCCAGGAACTGATCAAGCAGTTCCAGCAGCAGCAGACCGCCACTCTAGATGCCA
CACTCAATGTCATCAAGGAAGGCGAAGACCTTATCCAGCAGCTCAGGTCAGCGCCTCCCTCCCTCGGGGAGCCCAGCGAGGCCAGGGACTCGGCTGTGTCCAAC
AACAAAACACCCCACAGCAGCTCCATCAGCCACATCGAGTCGGTCCTGCAGCAGCTTGATGATGCCCAGGTGCAGATGGAGGAGCTGTTCCACGAGCGGAAGAT
CAAGCTGGACATCTTCCTGCAACTGCGCATCTTTGAGCAGTACACCATCGAGGTGACAGCAGAGCTAGACGCCTGGAATGAAGACTTGCTTCGGCAGATGAATGA
CTTCAACACAGAGGACCTAACCCTGGCAGAACAGCGGCTGCAGCGCCACACAGAACGGAAGCTAGCCATGAACAACATGACCTTTGAGGTTATCCAGCAGGGAC
AGGATCTGCACCAGTACATCACGGAGGTCCAGGCATCAGGAATTGAGTTGATCTGTGAAAAAGACATTGATCTGGCAGCCCAGGTGCAAGAGTTATTGGAATTTC
TCCATGAGAAGCAGCATGAATTGGAGCTCAATGCAGAGCAGACTCATAAGCGGCTAGAGCAGTGCCTCCAATTACGTCACCTCCAGGCTGAAGTCAAACAGGTTC
TGGGATGGATCCGCAATGGAGAGTCAATGCTCAACGCCAGCCTGGTCAATGCCAGCTCTTTGTCGGAAGCAGAGCAGCTGCAGCGGGAGCACGAGCAGTTCCA
ACTGGCCATCGAGTCCCTCTTTCATGCCACTTCCTTGCAGAAGACGCACCAGAGTGCCCTGCAGGTACAGCAGAAAGCCGAGGTGCTGCTCCAGGCCGGCCACTA
CGATGCCGATGCCATCCGGGAATGTGCTGAGAAGGTGGCCCTCCACTGGCAGCAGCTCATGCTGAAGATGGAAGACCGGCTAAAATTGGTCAATGCCTCTGTGGC
CTTTTACAAAACTTCTGAACAGGTGTGTAGTGTCCTGGAGAGCTTAGAGCAAGAATACCGGAGAGATGAGGACTGGTGTGGTGGACGAGATAAGCTGGGGCCA
GCAGCAGAGATCGACCATGTCATTCCCCTCATCAGCAAACATTTGGAACAAAAGGAGGCCTTTCTTAAGGCCTGCACCCTGGCTCGGCGGAATGCTGAGGTGTTT
CTCAAGTACATCCACAGGAACAACGTCAGCATGCCCAGTGTCGCCAGCCACACTCGGGGACCCGAGCAACAAGTGAAAGCCATCCTGAGTGAGCTCCTGCAGAG
GGAGAATCGCGTGCTGCATTTCTGGACCTTGAAGAAGCGGCGGTTAGACCAATGCCAGCAATATGTGGTGTTCGAGCGCAGCGCTAAGCAGGCGCTTGACTGGA
TCCAAGAAACAGGTGAATTTTACCTCTCAACACATACCTCCACTGGAGAGACCACAGAGGAGACTCAGGAACTGCTGAAAGAATATGGGGAATTCAGGGTGCCT
GCCAAGCAAACAAAGGAGAAGGTGAAGCTTCTGATTCAGCTGGCCGATAGCTTTGTGGAAAAAGGCCACATTCATGCCACGGAGATAAGGAAATGGGTGACCA
CGGTGGACAAGCACTACAGAGATTTCTCCCTGAGGATGGGAAAGTACCGATACTCACTGGAGAAAGCCCTAGGAGTCAACACAGAGGATAATAAGGACCTGGAG
CTGGATATTATCCCAGCAAGCCTTTCGGATCGGGAGGTCAAGCTGCGGGACGCCAACCACGAAGTCAATGAAGAGAAGCGGAAGTCAGCCCGGAAGAAAGAAT
TTATTATGGCTGAACTACTCCAGACAGAGAAGGCTTATGTAAGGGATTTGCATGAGTGCTTAGAGACCTACCTGTGGGAAATGACCAGTGGTGTGGAGGAGATCC
CCCCTGGGATCCTCAATAAAGAGCATATCATCTTTGGCAACATCCAAGAGATCTACGATTTCCATAACAACATCTTCCTCAAAGAGCTGGAGAAGTACGAGCAACTG
CCTGAGGATGTGGGACACTGCTTTGTTACCTGGGCAGACAAATTTCAGATGTATGTCACCTACTGTAAAAACAAGCCTGATTCCAACCAGCTTATCCTGGAGCATGC
GGGCACCTTCTTTGATGAGATACAACAGCGGCATGGTCTGGCCAACTCCATCTCTTCCTACCTAATTAAGCCTGTCCAAAGGATCACCAAATATCAACTGCTCCTGA
AGGAACTTTTAACTTGCTGTGAAGAAGGGAAAGGGGAGCTCAAGGATGGCCTGGAGGTGATGCTCAGTGTCCCAAAGAAAGCCAATGATGCCATGCATGTCAGC
ATGCTGGAAGGGTTCGACGAGAACCTGGATGTGCAGGGGGAGTTGATTCTCCAGGATGCCTTTCAAGTGTGGGACCCGAAGTCGCTGATCCGGAAGGGGCGGG
AGCGGCACTTGTTCCTCTTTGAGATCTCCTTGGTTTTTAGCAAGGAGATCAAAGATTCTTCAGGACACACGAAATATGTTTACAAGAACAAGCTACTGACCTCAGA
GCTGGGTGTGACCGAGCACGTGGAGGGCGATCCCTGCAAATTCGCCTTGTGGTCTGGGCGCACCCCATCCTCAGACAATAAAACAGTGCTGAAAGCCTCCAACAT
TGAAACCAAGCAGGAGTGGATCAAGAACATTCGAGAAGTGATTCAAGAAAGGATCATTCACCTGAAAGGAGCTTTAAAGGAGCCACTTCAGCTCCCCAAAACAC
CAGCCAAACAGAGGAACAATAGTAAGAGGGATGGAGTGGAGGATATTGACAGCCAGGGGGATGGGAGCAGCCAACCAGACACCATCTCCATTGCTTCTAGGAC
CTCTCAGAACACAGTGGACAGTGACAAGCTCTCTGGTGGATGTGAGCTGACAGTGGTCCTCCAGGACTTCAGTGCGGGCCACAGCAGTGAGCTGACCATCCAGG
TGGGGCAGACGGTAGAGCTGCTGGAGCGGCCCAGCGAGCGGCCTGGTTGGTGTCTGGTCCGTACCACCGAACGGAGCCCGCCCTTGGAGGGTCTGGTCCCCAG
CAGCGCCCTGTGCATCTCACACTCCCGAAGCAGCGTGGAGATGGACTGCTTCTTCCCCTTGGTGAAAGATGCATACTCTCATTCCTCAAGCGAGAATGGAGGCAA
GTCCGAGTCCGTGGCCAACCTGCAGGCCCAGCCCTCCCTGAACTCCATCCACAGTTCCCCGGGTCCCAAGCGCTCCACCAACACTCTTAAGAAGTGGCTGACGAG
TCCTGTGCGTCGGCTTAACAGCGGGAAGGCAGATGGAAACATCAAAAAGCAGAAGAAAGTTCGCGATGGTCGGAAGAGCTTTGACCTGGGATCTCCCAAGCCT
GGGGATGAAACAACCCCTCAGGGAGACAGCGCTGATGAGAAGAGCAAGAAAGGTTGGGGTGAAGATGAGCCGGATGAAGAGTCACACACACCCCTCCCACCA
CCTATGAAGATTTTTGACAACGACCCTACACAGGATGAAATGTCCTCCTCTTTGCTAGCAGCCCGGCAGGCTTCCACTGAAGTACCTACTGCTGCAGACCTTGTCAA
TGCAATAGAAAAGTTGGTCAAAAACAAGCTGAGTCTAGAAGGAAGCTCATACCGGGGGAGCTTGAAAGACCCTGCAGGCTGCCTGAATGAGGGGATGGCCCCA
CCCACACCTCCTAAAAACCCAGAAGAAGAACAGAAAGCCAAGGCCCTGAGAGGCAGGATGTTTGTCCTGAATGAGCTGGTACAGACAGAGAAAGACTATGTCA
AGGATCTGGGCATTGTGGTGGAGGGCTTCATGAAGAGAATAGAAGAAAAGGGTGTCCCTGAGGATATGCGAGGAAAGGACAAAATCGTGTTTGGAAATATTCAT
CAGATTTATGACTGGCATAAGGATTTTTTCCTGGCGGAACTGGAAAAGTGTATCCAGGAGCAAGACAGATTGGCACAGCTCTTTATTAAGCACGAGCGGAAGCTG
CACATCTACGTGTGGTATTGTCAGAATAAGCCGCGCTCAGAGTACATCGTTGCTGAGTATGACGCCTACTTTGAGGAGGTAAAACAGGAGATAAATCAGAGGCTGA
CACTGAGTGACTTCCTCATCAAGCCCATTCAGAGAATAACAAAATACCAGTTGCTCCTCAAGGACTTCCTGAGATACAGTGAGAAGGCTGGTTTGGAGTGTTCAGA
TATTGAGAAAGCAGTGGAGTTAATGTGCCTTGTTCCCAAACGCTGCAATGACATGATGAATCTAGGACGTCTGCAGGGCTTTGAGGGCACTCTGACTGCTCAGGG
GAAGCTGCTGCAGCAGGACACATTCTATGTGATCGAGCTGGATGCAGGCATGCAGTCCCGGACCAAAGAGAGGCGCGTGTTCCTCTTCGAGCAGATTGTCATCTT
CAGTGAACTGCTCAGGAAGGGATCCCTCACCCCTGGCTACATGTTCAAAAGGAGCATCAAGATGAATTACTTGGTCCTGGAGGAGAATGTGGACAATGATCCCTG
CAAGTTTGCACTCATGAACAGAGAGACTTCTGAGAGGGTTGTTCTGCAAGCCGCCAACGCTGACATCCAGCAGGCCTGGGTGCAGGACATCAATCAAGTCTTAG
AAACACAGCGAGACTTTTTGAATGCACTGCAATCGCCCATTGAGTATCAACGGAAAGAAAGGAGCACAGCTGTGATGAGGTCTCAACCTGCCAGGCTTCCCCAA
GCCAGCCCCAGGCCCTACTCCTCTGTTCCTGCGGGCTCAGAGAAGCCCCCAAAGGGCTCCAGCTATAACCCACCTCTGCCTCCCCTGAAGATATCTACCTCCAATG
GCAGTCCAGGGTTTGAATACCACCAGCCTGGGGACAAGTTCGAAGCCAGCAAGAACGACCTGGGAGGCTGCAATGGGACCTCGTCCATGGCCGTGATCAAAGA
TTACTATGCACTGAAGGAGAATGAAATCTGTGTGAGCCAAGGTGAGGTGGTCCAGGTCCTCGCCGTCAACCAGCAGAACATGTGTCTGGTGTACCAGCCTGCCAG
CGACCATTCCCCCGCCGCCGAGGGCTGGGTCCCAGGCAGCATCCTGGCGCCCCTCACCAAAGCCACAGCAGCAGAAAGTAGTGACGGGAGCATCAAGAAGTCA
TGTTCATGGCATACTCTACGCATGAGAAAGCGGGCGGAAGTGGAGAACACGGGTAAAAATGAAGCCACAGGGCCTCGTAAACCCAAGGATATTCTGGGCAACAA
AGTCTCTGTTAAAGAGACGAACAGTTCCGAGGAATCAGAGTGTGATGATCTTGACCCTAATACTAGCATGGAGATCTTAAATCCAAATTTCATCCAAGAAGTGGCC
CCAGAATTCCTTGTGCCCTTGGTGGATGTGACCTGCTTGCTTGGGGACACAGTGATACTGCAGTGCAAAGTCTGTGGGCGGCCAAAGCCCACCATCACTTGGAAG
GGTCCAGACCAGAACATCCTTGACACTGATAACAGCTCAGCCACATACACGGTCTCCTCTTGTGATTCTGGAGAAATCACCCTGAAGATCTGT

AATCTGATGCCCCAAGACAGTGGGATTTATACCTGCATAGCAACAAATGACCACGGGACCACATCAACGTCTGCAACAGTCAAAGTGCAAGGTGTTCCAGCAG
CCCCTAACCGCCCCATTGCCCAGGAGAGAAGCTGCACCTCCGTGATTCTCCGCTGGCTGCCCCCCTCCAGCACAGGAAACTGCACTATTTCTGGTTACACTGTG
GAGTACAGAGAGGAAGGTTCTCAGATCTGGCAGCAGTCAGTGGCTTCGACCTTGGACACTTACCTCGTCATCGAAGACCTTAGTCCCGGGTGTCCTTATCAGTT
CAGAGTCAGTGCCAGTAACCCCTGGGGAATCAGCCTTCCCAGCGAGCCCTCGGAGTTTGTGCGACTTCCAGAATATGATGCTGCTGCTGATGGTGCCACCATTT
CTTGGAAGGAAAATTTTGACTCAGCTTACACTGAGCTGAATGAAATTGGAAGAGGCCGTTTCTCTATAGTAAAGAAATGCATTCACAAAGCTACCCGCAAAGAT
GTGGCTGTGAAATTTGTTAGCAAAAAAATGAAGAAGAAAGAACAGGCTGCCCACGAGGCTGCCCTGCTTCAGCACCTACAGCACCCCCAGTACATCACTCTCC
ATGACACCTATGAGTCCCCCACATCCTACATCCTGATCTTGGAACTGATGGATGATGGCCGGCTCTTAGACTACCTTATGAATCATGATGAACTGATGGAGGAAAA
AGTAGCTTTCTATATCCGAGACATCATGGAGGCTCTGCAGTACCTTCACAACTGCAGGGTTGCACATTTGGACATAAAGCCTGAAAACCTGCTCATTGACCTACG
GATTCCAGTGCCTCGAGTGAAGCTCATTGACTTGGAGGATGCTGTCCAGATCTCGGGTCACTTCCACATTCACCACCTGCTGGGGAACCCTGAGTTTGCTGCCC
CAGAAGTCATTCAAGGCATCCCCGTCTCCCTGGGGACAGACATCTGGAGCATCGGGGTTCTGACATATGTCATGCTGAGTGGGGTCTCCCCCTTCTTGGATGAG
AGCAAAGAGGAGACATGTATCAACGTATGCAGGGTGGATTTCAGCTTCCCCCATGAATACTTCTGTGGTGTGAGCAATGCTGCCAGAGATTTCATCAATGTGATC
TTACAGGAAGATTTTCGGAGGCGGCCCACAGCAGCCACATGCTTGCAGCATCCATGGCTGCAGCCCCATAATGGCAGCTACTCTAAGATCCCCCTGGACACCTC
CCGCCTAGCATGCTTCATAGAACGTCGCAAGCACCAGAATGATGTGCGGCCTATTCCCAATGTCAAGAGCTACATTGTCAACCGGGTGAACCAAGGGACGTAG

>Isoform 515

ATGAACCCCCCTGAGGGAGCAGCGGAGGAAGGAGGAGCAGCAGACTCGGACGTGGACGCCTTTTTCCGGACAGGGTCTTTTCGGAATGATGGTTTGAAAGC
TTCTGATGTCCTTCCTATCCTAAAGGAAAAGGTGGCCTTCGTGTCTGGGGGTCGTGATAAGCGAGGCGGACCCATCCTGACCTTCCCTGCTCGCAGCAATCATG
ACAGAATAAGACAGGAAGACCTGCGGAAACTCGTGACGTATTTGGCCAGCGTGCCAAGTGAGGACGTGTGCAAACGTGGCTTCACTGTCATCATCGACATGCG
GGGCTCCAAGTGGGACCTCATCAAGCCCCTCCTCAAAACGCTGCAGGAAGCCTTTCCAGCTGAGATCCATGTGGCCCTCATCATTAAACCCGACAACTTCTGGC
AGAAACAGAAGACCAACTTTGGCAGCTCCAAATTCATCTTTGAGACGAGCATGGTATCTGTGGAGGGCCTCACAAAGCTGGTGGACCCCTCCCAGCTGACGG
AGGAGTTTGATGGCTCCCTGGACTACAACCATGAGGAGTGGATCGAACTGCGGCTCTCCCTGGAGGAGTTCTTCAACAGCGCCGTGCACCTGCTCTCGCGCCT
CGAGGACCTCCAGGAGATGCTAGCCCGGAAGGAGTTTCCTGTGGATGTGGAGGGCTCTCGGCGGCTCATTGACGAACACACACAGCTCAAGAAAAAGGTGC
TGAAGGCCCCTGTGGAGGAGCTGGACCGGGAGGGGCAGCGGCTGCTGCAGTGCATCCGCTGCAGCGACGGCTTCTCAGGACGCAACTGCATCCCGGGCAGT
GCTGACTTCCAGAGCCTGGTGCCCAAGATCACCAGTCTCCTGGACAAGCTGCACTCCACCCGGCAGCACCTGCACCAGATGTGGCATGTGCGCAAGCTCAAGC
TGGACCAGTGCTTTCAGCTGCGGCTCTTCGAGCAGGATGCTGAGAAGATGTTTGACTGGATAAGCCACAACAAGGAGTTATTCCTCCAGAGCCACACGGAGAT
CGGAGTCAGCTACCAGTACGCCCTTGACCTCCAGACGCAGCACAATCACTTTGCCATGAACTCCATGAATGCCTATGTCAACATCAACCGCATCATGTCCGTGGC
TTCCCGCCTCTCTGAGGCCGGTCATTATGCCTCACAACAAATCAAGCAGATCTCCACCCAGCTGGACCAGGAGTGGAAGAGCTTCGCTGCTGCCCTGGATGAAC
GCAGCACCATCCTCGCCATGTCTGCTGTGTTCCACCAGAAGGCTGAGCAGTTCCTGTCGGGAGTGGATGCCTGGTGCAAGATGTGCAGTGAAGGTGGTCTGCC
ATCCGAGATGCAAGACCTAGAGCTGGCAATCCACCACCACCAGACCTTGTATGAGCAGGTGACCCAAGCCTACACAGAGGTCAGCCAGGATGGCAAAGCACT
ACTTGATGTGCTGCAGCGGCCCCTGAGCCCTGGGAACTCCGAATCCCTCACGGCCACAGCCAACTACTCCAAGGCAGTGCACCAGGTGCTGGACGTGGTGCAT
GAGGTGTTACATCACCAGCGACGGCTGGAGAGCATCTGGCAGCACCGCAAGGTGCGGCTCCACCAGCGGCTGCAGCTCTGCGTCTTCCAGCAGGATGTACAG
CAGGTGTTGGACTGGATTGAAAACCATGGTGAGGCCTTTCTCAGCAAACACACTGGAGTTGGGAAGTCCCTACATCGAGCCCGGGCCCTGCAGAAGAGGCAT
GATGACTTTGAAGAGGTGGCTCAGAATACGTACACCAATGCGGACAAGCTCCTAGAAGCAGCAGAGCAGTTGGCTCAGACGGGGGAATGTGACCCCGAGGA
GATCTACAAGGCAGCTCGACACCTGGAGGTGCGCATCCAAGACTTCGTGCGCAGGGTGGAGCAGCGGAAGCTTCTCCTGGACATGTCTGTTTCCTTCCACACA
CACACCAAAGAGTTGTGGACATGGATGGAAGACCTTCAGAAGGAGATGTTGGAGGATGTCTGTGCAGATTCTGTGGATGCAGTCCAGGAACTGATCAAGCAG
TTCCAGCAGCAGCAGACCGCCACTCTAGATGCCACACTCAATGTCATCAAGGAAGGCGAAGACCTTATCCAGCAGCTCAGGTCAGCGCCTCCCTCCCTCGGGG
AGCCCAGCGAGGCCAGGGACTCGGCTGTGTCCAACAACAAAACACCCCACAGCAGCTCCATCAGCCACATCGAGTCGGTCCTGCAGCAGCTTGATGATGCCC
AGGTGCAGATGGAGGAGCTGTTCCACGAGCGGAAGATCAAGCTGGACATCTTCCTGCAACTGCGCATCTTTGAGCAGTACACCATCGAGGTGACAGCAGAGC
TAGACGCCTGGAATGAAGACTTGCTTCGGCAGATGAATGACTTCAACACAGAGGACCTAACCCTGGCAGAACAGCGGCTGCAGCGCCACACAGAACGGAAG
CTAGCCATGAACAACATGACCTTTGAGGTTATCCAGCAGGGACAGGATCTGCACCAGTACATCACGGAGGTCCAGGCATCAGGAATTGAGTTGATCTGTGAAA
AAGACATTGATCTGGCAGCCCAGGTGCAAGAGTTATTGGAATTTCTCCATGAGAAGCAGCATGAATTGGAGCTCAATGCAGAGCAGACTCATAAGCGGCTAGA
GCAGTGCCTCCAATTACGTCACCTCCAGGCTGAAGTCAAACAGGTTCTGGGATGGATCCGCAATGGAGAGTCAATGCTCAACGCCAGCCTGGTCAATGCCAGC
TCTTTGTCGGAAGCAGAGCAGCTGCAGCGGGAGCACGAGCAGTTCCAACTGGCCATCGAGTCCCTCTTTCATGCCACTTCCTTGCAGAAGACGCACCAGAGT
GCCCTGCAGGTACAGCAGAAAGCCGAGGTGCTGCTCCAGGCCGGCCACTACGATGCCGATGCCATCCGGGAATGTGCTGAGAAGGTGGCCCTCCACTGGCAG
CAGCTCATGCTGAAGATGGAAGACCGGCTAAAATTGGTCAATGCCTCTGTGGCCTTTTACAAAACTTCTGAACAGGTGTGTAGTGTCCTGGAGAGCTTAGAGCA
AGAATACCGGAGAGATGAGGACTGGTGTGGTGGACGAGATAAGCTGGGGCCAGCAGCAGAGATCGACCATGTCATTCCCCTCATCAGCAAACATTTGGAACA
AAAGGAGGCCTTTCTTAAGGCCTGCACCCTGGCTCGGCGGAATGCTGAGGTGTTTCTCAAGTACATCCACAGGAACAACGTCAGCATGCCCAGTGTCGCCAGC
CACACTCGGGGACCCGAGCAACAAGTGAAAGCCATCCTGAGTGAGCTCCTGCAGAGGGAGAATCGCGTGCTGCATTTCTGGACCTTGAAGAAGCGGCGGTTA
GACCAATGCCAGCAATATGTGGTGTTCGAGCGCAGCGCTAAGCAGGCGCTTGACTGGATCCAAGAAACAGGTGAATTTTACCTCTCAACACATACCTCCACTGG
AGAGACCACAGAGGAGACTCAGGAACTGCTGAAAGAATATGGGGAATTCAGGGTGCCTGCCAAGCAAACAAAGGAGAAGGTGAAGCTTCTGATTCAGCTGG
CCGATAGCTTTGTGGAAAAAGGCCACATTCATGCCACGGAGATAAGGAAATGGGTGACCACGGTGGACAAGCACTACAGAGATTTCTCCCTGAGGATGGGAA
AGTACCGATACTCACTGGAGAAAGCCCTAGGAGTCAACACAGAGGATAATAAGGACCTGGAGCTGGATATTATCCCAGCAAGCCTTTCGGATCGGGAGGTCAA
GCTGCGGGACGCCAACCACGAAGTCAATGAAGAGAAGCGGAAGTCAGCCCGGAAGAAAGAATTTATTATGGCTGAACTACTCCAGACAGAGAAGGCTTATGT
AAGGGATTTGCATGAGTGCTTAGAGACCTACCTGTGGGAAATGACCAGTGGTGTGGAGGAGATCCCCCCTGGGATCCTCAATAAAGAGCATATCATCTTTGGCA
ACATCCAAGAGATCTACGATTTCCATAACAACATCTTCCTCAAAGAGCTGGAGAAGTACGAGCAACTGCCTGAGGATGTGGGACACTGCTTTGTTACCTGGGCA
GACAAATTTCAGATGTATGTCACCTACTGTAAAAACAAGCCTGATTCCAACCAGCTTATCCTGGAGCATGCGGGCACCTTCTTTGATGAGATACAACAGCGGCAT
GGTCTGGCCAACTCCATCTCTTCCTACCTAATTAAGCCTGTCCAAAGGATCACCAAATATCAACTGCTCCTGAAGGAACTTTTAACTTGCTGTGAAGAAGGGAAA
GGGGAGCTCAAGGATGGCCTGGAGGTGATGCTCAGTGTCCCAAAGAAAGCCAATGATGCCATGCATGTCAGCATGCTGGAAGGGTTCGACGAGAACCTGGAT
GTGCAGGGGGAGTTGATTCTCCAGGATGCCTTTCAAGTGTGGGACCCGAAGTCGCTGATCCGGAAGGGGCGGGAGCGGCACTTGTTCCTCTTTGAGATCTCC
TTGGTTTTTAGCAAGGAGATCAAAGATTCTTCAGGACACACGAAATATGTTTACAAGAACAAGCTACTGACCTCAGAGCTGGGTGTGACCGAGCACGTGGAGG
GCGATCCCTGCAAATTCGCCTTGTGGTCTGGGCGCACCCCATCCTCAGACAATAAAACAGTGCTGAAAGCCTCCAACATTGAAACCAAGCAGGAGTGGATCAA
GAACATTCGAGAAGTGATTCAAGAAAGGATCATTCACCTGAAAGGAGCTTTAAAGGAGCCACTTCAGCTCCCCAAAACACCAGCCAAACAGAGGAACAATAG
TAAGAGGGATGGAGTGGAGGATATTGACAGCCAGGGGGATGGGAGCAGCCAACCAGACACCATCTCCATTGCTTCTAGGACCTCTCAGAACACAGTGGACAG
TGACAAGCTCTCTGGTGGATGTGAGCTGACAGTGGTCCTCCAGGACTTCAGTGCGGGCCACAGCAGTGAGCTGACCATCCAGGTGGGGCAGACGGTAGAGCT
GCTGGAGCGGCCCAGCGAGCGGCCTGGTTGGTGTCTGGTCCGTACCACCGAACGGAGCCCGCCCTTGGAGGGTCTGGTCCCCAGCAGCGCCCTGTGCATCTC
ACACTCCCGAAGCAGCGTGGAGATGGACTGCTTCTTCCCCTTGGTGAAAGATGCATACTCTCATTCCTCAAGCGAGAATGGAGGCAAGTCCGAGTCCGTGGCC
AACCTGCAGGCCCAGCCCTCCCTGAACTCCATCCACAGTTCCCCGGGTCCCAAGCGCTCCACCAACACTCTTAAGAAGTGGCTGACGAGTCCTGTGCGTCGGC
TTAACAGCGGGAAGGCAGATGGAAACATCAAAAAGCAGAAGAAAGTTCGCGATGGTCGGAAGAGCTTTGACCTGGGATCTCCCAAGCCTGGGGATGAAACA
ACCCCTCAGGGAGACAGCGCTGATGAGAAGAGCAAGAAAGGTTGGGGTGAAGATGAGCCGGATGAAGAGTCACACACACCCCTCCCACCACCTATGAAGAT
TTTTGACAACGACCCTACACAGGATGAAATGTCCTCCTCTTTGCTAGCAGCCCGGCAGGCTTCCACTGAAGTACCTACTGCTGCAGACCTTGTCAATGCAATAGA
AAAGTTGGTCAAAAACAAGCTGAGTCTAGAAGGAAGCTCATACCGGGGGAGCTTGAAAGACCCTGCAGGCTGCCTGAATGAGGGGATGGCCCCACCCACAC
CTCCTAAAAACCCAGAAGAAGAACAGAAAGCCAAGGCCCTGAGAGGCAGGATGTTTGTCCTGAATGAGCTGGTACAGACAGAGAAAGACTATGTCAAGGATC
TGGGCATTGTGGTGGAGGGCTTCATGAAGAGAATAGAAGAAAAGGGTGTCCCTGAGGATATGCGAGGAAAGGACAAAATCGTGTTTGGAAATATTCATCAGA
TTTATGACTGGCATAAGGATTTTTTCCTGGCGGAACTGGAAAAGTGTATCCAGGAGCAAGACAGATTGGCACAGCTCTTTATTAAGCACGAGCGGAAGCTGCA
CATCTACGTGTGGTATTGTCAGAATAAGCCGCGCTCAGAGTACATCGTTGCTGAGTATGACGCCTACTTTGAGGAGGTAAAACAGGAGATAAATCAGAGGCTGA
CACTGAGTGACTTCCTCATCAAGCCCATTCAGAGAATAACAAAATACCAGTTGCTCCTCAAGGACTTCCTGAGATACAGTGAGAAGGCTGGTTTGGAGTGTTCA
GATATTGAGAAAGCAGTGGAGTTAATGTGCCTTGTTCCCAAACGCTGCAATGACATGATGAATCTAGGACGTCTGCAGGGCTTTGAGGGCACTCTGACTGCTCA
GGGGAAGCTGCTGCAGCAGGACACATTCTATGTGATCGAGCTGGATGCAGGCATGCAGTCCCGGACCAAAGAGAGGCGCGTGTTCC

TCTTCGAGCAGATTGTCATCTTCAGTGAACTGCTCAGGAAGGGATCCCTCACCCCTGGCTACATGTTCAAAAGGAGCATCAAGATGAATTACTTGGTCCTGGAGG
AGAATGTGGACAATGATCCCTGCAAGTTTGCACTCATGAACAGAGAGACTTCTGAGAGGGTTGTTCTGCAAGCCGCCAACGCTGACATCCAGCAGGCCTGGGT
GCAGGACATCAATCAAGTCTTAGAAACACAGCGAGACTTTTTGAATGCACTGCAATCGCCCATTGAGTATCAACGGAAAGAAAGGAGCACAGCTGTGATGAGG
TCTCAACCTGCCAGGCTTCCCCAAGCCAGCCCCAGGCCCTACTCCTCTGTTCCTGCGGGCTCAGAGAAGCCCCCAAAGGGCTCCAGCTATAACCCACCTCTGCCT
CCCCTGAAGATATCTACCTCCAATGGCAGTCCAGGGTTTGAATACCACCAGCCTGGGGACAAGTTCGAAGCCAGCAAGAACGACCTGGGAGGCTGCAATGGGA
CCTCGTCCATGGCCGTGATCAAAGATTACTATGCACTGAAGGAGAATGAAATCTGTGTGAGCCAAGGTGAGGTGGTCCAGGTCCTCGCCGTCAACCAGCAGAAC
ATGTGTCTGGTGTACCAGCCTGCCAGCGACCATTCCCCCGCCGCCGAGGGCTGGGTCCCAGGCAGCATCCTGGCGCCCCTCACCAAAGCCACAGCAGCAGAAA
GTAGTGACGGGAGCATCAAGTAA

>Isoform 245

ATGAACCCCCCTGAGGGAGCAGCGGAGGAAGGAGGAGCAGCAGACTCGGACGTGGACGCCTTTTTCCGGACAGGGTCTTTTCGGAATGATGGTTTGAAAGC
TTCTGATGTCCTTCCTATCCTAAAGGAAAAGGTGGCCTTCGTGTCTGGGGGTCGTGATAAGCGAGGCGGACCCATCCTGACCTTCCCTGCTCGCAGCAATCATGA
CAGAATAAGACAGGAAGACCTGCGGAAACTCGTGACGTATTTGGCCAGCGTGCCAAGTGAGGACGTGTGCAAACGTGGCTTCACTGTCATCATCGACATGCGG
GGCTCCAAGTGGGACCTCATCAAGCCCCTCCTCAAAACGCTGCAGGAAGCCTTTCCAGCTGAGATCCATGTGGCCCTCATCATTAAACCCGACAACTTCTGGCA
GAAACAGAAGACCAACTTTGGCAGCTCCAAATTCATCTTTGAGACGAGCATGGTATCTGTGGAGGGCCTCACAAAGCTGGTGGACCCCTCCCAGCTGACGGAG
GAGTTTGATGGCTCCCTGGACTACAACCATGAGGAGTGGATCGAACTGCGGCTCTCCCTGGAGGAGTTCTTCAACAGCGCCGTGCACCTGCTCTCGCGCCTCGA
GGACCTCCAGGAGATGCTAGCCCGGAAGGAGTTTCCTGTGGATGTGGAGGGCTCTCGGCGGCTCATTGACGAACACACACAGCTCAAGAAAAAGGTGCTGAA
GGCCCCTGTGGAGGAGCTGGACCGGGAGGGGCAGCGGCTGCTGCAGTGCATCCGCTGCAGCGACGGCTTCTCAGGACGCAACTGCATCCCGGGCAGTGCTG
ACTTCCAGAGCCTGGTGCCCAAGATCACCAGTCTCCTGGACAAGCTGCACTCCACCCGGCAGCACCTGCACCAGATGTGGCATGTGCGCAAGCTCAAGCTGGA
CCAGTGCTTTCAGCTGCGGCTCTTCGAGCAGGATGCTGAGAAGATGTTTGACTGGATAAGCCACAACAAGGAGTTATTCCTCCAGAGCCACACGGAGATCGGA
GTCAGCTACCAGTACGCCCTTGACCTCCAGACGCAGCACAATCACTTTGCCATGAACTCCATGAATGCCTATGTCAACATCAACCGCATCATGTCCGTGGCTTCCC
GCCTCTCTGAGGCCGGTCATTATGCCTCACAACAAATCAAGCAGATCTCCACCCAGCTGGACCAGGAGTGGAAGAGCTTCGCTGCTGCCCTGGATGAACGCAG
CACCATCCTCGCCATGTCTGCTGTGTTCCACCAGAAGGCTGAGCAGTTCCTGTCGGGAGTGGATGCCTGGTGCAAGATGTGCAGTGAAGGTGGTCTGCCATCCG
AGATGCAAGACCTAGAGCTGGCAATCCACCACCACCAGACCTTGTATGAGCAGGTGACCCAAGCCTACACAGAGGTCAGCCAGGATGGCAAAGCACTACTTGA
TGTGCTGCAGCGGCCCCTGAGCCCTGGGAACTCCGAATCCCTCACGGCCACAGCCAACTACTCCAAGGCAGTGCACCAGGTGCTGGACGTGGTGCATGAGGTG
TTACATCACCAGCGACGGCTGGAGAGCATCTGGCAGCACCGCAAGGTGCGGCTCCACCAGCGGCTGCAGCTCTGCGTCTTCCAGCAGGATGTACAGCAGGTGT
TGGACTGGATTGAAAACCATGGTGAGGCCTTTCTCAGCAAACACACTGGAGTTGGGAAGTCCCTACATCGAGCCCGGGCCCTGCAGAAGAGGCATGATGACTT
TGAAGAGGTGGCTCAGAATACGTACACCAATGCGGACAAGCTCCTAGAAGCAGCAGAGCAGTTGGCTCAGACGGGGGAATGTGACCCCGAGGAGATCTACAA
GGCAGCTCGACACCTGGAGGTGCGCATCCAAGACTTCGTGCGCAGGGTGGAGCAGCGGAAGCTTCTCCTGGACATGTCTGTTTCCTTCCACACACACACACCAAA
GAGTTGTGGACATGGATGGAAGACCTTCAGAAGGAGATGTTGGAGGATGTCTGTGCAGATTCTGTGGATGCAGTCCAGGAACTGATCAAGCAGTTCCAGCAGC
AGCAGACCGCCACTCTAGATGCCACACTCAATGTCATCAAGGAAGGCGAAGACCTTATCCAGCAGCTCAGGTCAGCGCCTCCCTCCCTCGGGGAGCCCAGCGA
GGCCAGGGACTCGGCTGTGTCCAACAACAAAACACCCCACAGCAGCTCCATCAGCCACATCGAGTCGGTCCTGCAGCAGCTTGATGATGCCCAGGTGCAGATG
GAGGAGCTGTTCCACGAGCGGAAGATCAAGCTGGACATCTTCCTGCAACTGCGCATCTTTGAGCAGTACACCATCGAGGTGACAGCAGAGCTAGACGCCTGGA
ATGAAGACTTGCTTCGGCAGATGAATGACTTCAACACAGAGGACCTAACCCTGGCAGAACAGCGGCTGCAGCGCCACACAGAACGGAAGCTAGCCATGAACA
ACATGACCTTTGAGGTTATCCAGCAGGGACAGGATCTGCACCAGTACATCACGGAGGTCCAGGCATCAGGAATTGAGTTGATCTGTGAAAAAGACATTGATCTG
GCAGCCCAGGTGCAAGAGTTATTGGAATTTCTCCATGAGAAGCAGCATGAATTGGAGCTCAATGCAGAGCAGACTCATAAGCGGCTAGAGCAGTGCCTCCAATT
ACGTCACCTCCAGGCTGAAGTCAAACAGGTTCTGGGATGGATCCGCAATGGAGAGTCAATGCTCAACGCCAGCCTGGTCAATGCCAGCTCTTTGTCGGAAGCA
GAGCAGCTGCAGCGGGAGCACGAGCAGTTCCAACTGGCCATCGAGTCCCTCTTTCATGCCACTTCCTTGCAGAAGACGCACCAGAGTGCCCTGCAGGTACAGC
AGAAAGCCGAGGTGCTGCTCCAGGCCGGCCACTACGATGCCGATGCCATCCGGGAATGTGCTGAGAAGGTGGCCCTCCACTGGCAGCAGCTCATGCTGAAGAT
GGAAGACCGGCTAAAATTGGTCAATGCCTCTGTGGCCTTTTACAAAACTTCTGAACAGGTGTGTAGTGTCCTGGAGAGCTTAGAGCAAGAATACCGGAGAGAT
GAGGACTGGTGTGGTGGACGAGATAAGCTGGGGCCAGCAGCAGAGATCGACCATGTCATTCCCCTCATCAGCAAACATTTGGAACAAAAGGAGGCCTTTCTTA
AGGCCTGCACCCTGGCTCGGCGGAATGCTGAGGTGTTTCTCAAGTACATCCACAGGAACAACGTCAGCATGCCCAGTGTCGCCAGCCACACTCGGGGACCCGA
GCAACAAGTGAAAGCCATCCTGAGTGAGCTCCTGCAGAGGGAGAATCGCGTGCTGCATTTCTGGACCTTGAAGAAGCGGCGGTTAGACCAATGCCAGCAATAT
GTGGTGTTCGAGCGCAGCGCTAAGCAGGCGCTTGACTGGATCCAAGAAACAGGTGAATTTTACCTCTCAACACATACCTCCACTGGAGAGACCACAGAGGAGA
CTCAGGAACTGCTGAAAGAATATGGGGAATTCAGGGTGCCTGCCAAGCAAACAAAGGAGAAGGTGAAGCTTCTGATTCAGCTGGCCGATAGCTTTGTGGAAA
AAGGCCACATTCATGCCACGGAGATAAGGAAATGGGTGACCACGGTGGACAAGCACTACAGAGATTTCTCCCTGAGGATGGGAAAGTACCGATACTCACTGGA
GAAAGCCCTAGGAGTCAACACAGAGGATAATAAGGACCTGGAGCTGGATATTATCCCAGCAAGCCTTTCGGATCGGGAGGTCAAGCTGCGGGACGCCAACCAC
GAAGTCAATGAAGAGAAGCGGAAGTCAGCCCGGAAGAAAGAATTTATTATGGCTGAACTACTCCAGACAGAGAAGGCTTATGTAAGGGATTTGCATGAGTGCT
TAGAGACCTACCTGTGGGAAATGACCAGTGGTGTGGAGGAGATCCCCCCTGGGATCCTCAATAAAGAGCATATCATCTTTGGCAACATCCAAGAGATCTACGATT
TCCATAACAACATCTTCCTCAAAGAGCTGGAGAAGTACGAGCAACTGCCTGAGGATGTGGGACACTGCTTTGTTACCTGGGCAGACAAATTTCAGATGTATGTC
ACCTACTGTAAAAACAAGCCTGATTCCAACCAGCTTATCCTGGAGCATGCGGGCACCTTCTTTGATGAGATACAACAGCGGCATGGTCTGGCCAACTCCATCTCT
TCCTACCTAATTAAGCCTGTCCAAAGGATCACCAAATATCAACTGCTCCTGAAGGAACTTTTAACTTGCTGTGAAGAAGGGAAAGGGGAGCTCAAGGATGGCCT
GGAGGTGATGCTCAGTGTCCCAAAGAAAGCCAATGATGCCATGCATGTCAGCATGCTGGAAGGGTTCGACGAGAACCTGGATGTGCAGGGGGAGTTGATTCTC
CAGGATGCCTTTCAAGTGTGGGACCCGAAGTCGCTGATCCGGAAGGGGCGGGAGCGGCACTTGTTCCTCTTTGAGATCTCCTTGGTTTTTAGCAAGGAGATCA
AAGATTCTTCAGGACACACGAAATATGTTTACAAGAACAAGCTACTGACCTCAGAGCTGGGTGTGACCGAGCACGTGGAGGGCGATCCCTGCAAATTCGCCTT
GTGGTCTGGGCGCACCCCATCCTCAGACAATAAAACAGTGCTGAAAGCCTCCAACATTGAAACCAAGCAGGAGTGGATCAAGAACATTCGAGAAGTGATTCAA
GAAAGGATCATTCACCTGAAAGGAGCTTTAAAGGAGCCACTTCAGCTCCCCAAAACACCAGCCAAACAGAGGAACAATAGTAAGAGGGATGGAGTGGAGGAT
ATTGACAGCCAGGGGGATGGGAGCAGCCAACCAGACACCATCTCCATTGCTTCTAGGACCTCTCAGAACACAGTGGACAGTGACAAGGATGGCAACCTTGTTC
CTCGGTGGCACCTGGGACCTGGAGATCCTTTCTCCACTTACGTTTAG

>Isoform 149

ATGACGGACCGCTTCTGGGACCAGTGGTATCTCTGGTATCTCCGCTTGCTCCGGCTGCTGGATCGAGGGTCTTTTCGGAATGATGGTTTGAAAGCTTCTGATGTC
CTTCCTATCCTAAAGGAAAAGGTGGCCTTCGTGTCTGGGGGTCGTGATAAGCGAGGCGGACCCATCCTGACCTTCCCTGCTCGCAGCAATCATGACAGAATAAG
ACAGGAAGACCTGCGGAAACTCGTGACGTATTTGGCCAGCGTGCCAAGTGAGGACGTGTGCAAACGTGGCTTCACTGTCATCATCGACATGCGGGGCTCCAAG
TGGGACCTCATCAAGCCCCTCCTCAAAACGCTGCAGGAAGCCTTTCCAGCTGAGATCCATGTGGCCCTCATCATTAAACCCGACAACTTCTGGCAGAAACAGAA
GACCAACTTTGGCAGCTCCAAATTCATCTTTGAGACGAGCATGGTATCTGTGGAGGGCCTCACAAAGCTGGTGGACCCCTCCCAGCTGACGGAGGAGTTTGAT
GGCTCCCTGGACTACAACCATGAGGAGTGGATCGAACTGCGGCTCTCCCTGGAGGAGTTCTTCAACAGCGCCGTGCACCTGCTCTCGCGCCTCGAGGACCTCC
AGGAGATGCTAGCCCGGAAGGAGTTTCCTGTGGATGTGGAGGGCTCTCGGCGGCTCATTGACGAACACACACAGCTCAAGAAAAAGGTGCTGAAGGCCCCTG
TGGAGGAGCTGGACCGGGAGGGGCAGCGGCTGCTGCAGTGCATCCGCTGCAGCGACGGCTTCTCAGGACGCAACTGCATCCCGGGCAGTGCTGACTTCCAG
AGCCTGGTGCCCAAGATCACCAGTCTCCTGGACAAGCTGCACTCCACCCGGCAGCACCTGCACCAGATGTGGCATGTGCGCAAGCTCAAGCTGGACCAGTGCT
TTCAGCTGCGGCTCTTCGAGCAGGATGCTGAGAAGATGTTTGACTGGATAAGCCACAACAAGGAGTTATTCCTCCAGAGCCACACGGAGATCGGAGTCAGCTA
CCAGTACGCCCTTGACCTCCAGACGCAGCACAATCACTTTGCCATGAACTCCATGAATGCCTATGTCAACATCAACCGCATCATGTCCGTGGCTTCCCGCCTCTCT
GAGGCCGGTCATTATGCCTCACAACAAATCAAGCAGATCTCCACCCAGCTGGACCAGGAGTGGAAGAGCTTCGCTGCTGCCCTGGATGAACGCAGCACCATCC
TCGCCATGTCTGCTGTGTTCCACCAGAAGGCTGAGCAGTTCCTGTCGGGAGTGGATGCCTGGTGCAAGATGTGCAGTGAAGGTGGTCTGCCATCCGAGATGC

AAGACCTAGAGCTGGCAATCCACCACCACCAGACCTTGTATGAGCAGGTGACCCAAGCCTACACAGAGGTCAGCCAGGATGGCAAAGCACTACTTGATGTG
CTGCAGCGGCCCCTGAGCCCTGGGAACTCCGAATCCCTCACGGCCACAGCCAACTACTCCAAGGCAGTGCACCAGGTGCTGGACGTGGTGCATGAGGTGT
TACATCACCAGCGACGGCTGGAGAGCATCTGGCAGCACCGCAAGGTGCGGCTCCACCAGCGGCTGCAGCTCTGCGTCTTCCAGCAGGATGTACAGCAGGT
GTTGGACTGGATTGAAAACCATGGTGAGGCCTTTCTCAGCAAACACACTGGAGTTGGGAAGTCCCTACATCGAGCCCGGGCCCTGCAGAAGAGGCATGAT
GACTTTGAAGAGGTGGCTCAGAATACGTACACCAATGCGGACAAGCTCCTAGAAGCAGCAGAGCAGTTGGCTCAGACGGGGGAATGTGACCCCGAGGAG
ATCTACAAGGCAGCTCGACACCTGGAGGTGCGCATCCAAGACTTCGTGCGCAGGGTGGAGCAGCGGAAGCTTCTCCTGGACATGTCTGTTTCCTTCCACAC
ACACACCAAAGAGTTGTGGACATGGATGGAAGACCTTCAGAAGGAGATGTTGGAGGATGTCTGTGCAGATTCTGTGGATGCAGTCCAGGAACTGATCAAG
CAGTTCCAGCAGCAGCAGACCGCCACTCTAGATGCCACACTCAATGTCATCAAGGAAGGCGAAGACCTTATCCAGCAGCTCAGGTCAGCGCCTCCCTCCCTC
GGGGAGCCCAGCGAGGCCAGGGACTCGGCTGTGTCCAACAACAAAACACCCCACAGCAGCTCCATCAGCCACATCGAGTCGGTCCTGCAGCAGCTTGATG
ATGCCCAGGTGCAGATGGAGGAGCTGTTCCACGAGCGGAAGATCAAGCTGGACATCTTCCTGCAACTGCGCATCTTTGAGCAGTACACCATCGAGGTGACA
GCAGAGCTAGACGCCTGGAATGAAGACTTGCTTCGGCAGATGAATGACTTCAACACAGAGGACCTAACCCTGGCAGAACAGCGGCTGCAGCGCCACACAG
AACGGAAGCTAGCCATGAACAACATGACCTTTGAGGTTATCCAGCAGGGACAGGATCTGCACCAGTACATCACGGAGGTCCAGGCATCAGGAATTGAGTTG
ATCTGTGAAAAAGACATTGATCTGGCAGCCCAGGTGCAAGAGTTATTGGAATTTCTCCATGAGAAGCAGCATGAATTGGAGCTCAATGCAGAGCAGACTCAT
AAGCGGCTAGAGCAGTGCCTCCAATTACGTCACCTCCAGGCTGAAGTCAAACAGGTTCTGGGATGGATCCGCAATGGAGAGTCAATGCTCAACGCCAGCCT
GGTCAATGCCAGCTCTTTGTCGGAAGCAGAGCAGCTGCAGCGGGAGCACGAGCAGTTCCAACTGGCCATCGAGTCCCTCTTTCATGCCACTTCCTTGCAGA
AGACGCACCAGAGTGCCCTGCAGGTACAGCAGAAAGCCGAGGTGCTGCTCCAGGCCGGCCACTACGATGCCGATGCCATCCGGGAATGTGCTGAGAAGGT
GGCCCTCCACTGGCAGCAGCTCATGCTGAAGATGGAAGACCGGCTAAAATTGGTCAATGCCTCTGTGGCCTTTTACAAAACTTCTGAACAGGTGTGTAGTGT
CCTGGAGAGCTTAGAGCAAGAATACCGGAGAGATGAGGACTGGTGTGGTGGACGAGATAAGCTGGGGCCAGCAGCAGAGATCGACCATGTCATTCCCCTC
ATCAGCAAACATTTGGAACAAAAGGAGGCCTTTCTTAAGGCCTGCACCCTGGCTCGGCGGAATGCTGAGGTGTTTCTCAAGTACATCCACAGGAACAACGT
CAGCATGCCCAGTGTCGCCAGCCACACTCGGGGACCCGAGCAACAAGTGAAAGCCATCCTGAGTGAGCTCCTGCAGAGGGAGAATCGCGTGCTGCATTTC
TGGACCTTGAAGAAGCGGCGGTTAGACCAATGCCAGCAATATGTGGTGTTCGAGCGCAGCGCTAAGCAGGCGCTTGACTGGATCCAAGAAACAGGTGAAT
TTTACCTCTCAACACATACCTCCACTGGAGAGACCACAGAGGAGACTCAGGAACTGCTGAAAGAATATGGGGAATTCAGGGTGCCTGCCAAGCAAACAAA
GGAGAAGGTGAAGCTTCTGATTCAGCTGGCCGATAGCTTTGTGGAAAAAGGCCACATTCATGCCACGGAGATAAGGAAATGGGTGACCACGGTGGACAAG
CACTACAGAGATTTCTCCCTGAGGATGGGAAAGTACCGATACTCACTGGAGAAAGCCCTAGGAGTCAACACAGAGGATAATAAGGACCTGGAGCTGGATAT
TATCCCAGCAAGCCTTTCGGATCGGGAGGTCAAGCTGCGGGACGCCAACCACGAAGTCAATGAAGAGAAGCGGAAGTCAGCCCGGAAGAAAGAATTTATT
ATGGCTGAACTACTCCAGACAGAGAAGGCTTATGTAAGGGATTTGCATGAGTGCTTAGAGACCTACCTGTGGGAAATGACCAGTGGTGTGGAGGAGATCCC
CCCTGGGATCCTCAATAAAGAGCATATCATCTTTGGCAACATCCAAGAGATCTACGATTTCCATAACAACATCTTCCTCAAAGAGCTGGAGAAGTACGAGCAA
CTGCCTGAGGATGTGGGACACTGCTTTGTTACCTGGGCAGACAAATTTCAGATGTATGTCACCTACTGTAAAAACAAGCCTGATTCCAACCAGCTTATCCTGG
AGCATGCGGGCACCTTCTTTGATGAGATACAACAGCGGCATGGTCTGGCCAACTCCATCTCTTCCTACCTAATTAAGCCTGTCCAAAGGATCACCAAATATCA
ACTGCTCCTGAAGGAACTTTTAACTTGCTGTGAAGAAGGGAAAGGGGAGCTCAAGGATGGCCTGGAGGTGATGCTCAGTGTCCCAAAGAAAGCCAATGAT
GCCATGCATGTCAGCATGCTGGAAGGGTTCGACGAGAACCTGGATGTGCAGGGGGAGTTGATTCTCCAGGATGCCTTTCAAGTGTGGGACCCGAAGTCGC
TGATCCGGAAGGGGCGGGAGCGGCACTTGTTCCTCTTTGAGATCTCCTTGGTTTTTAGCAAGGAGATCAAAGATTCTTCAGGACACACGAAATATGTTTACA
AGAACAAGCTACTGACCTCAGAGCTGGGTGTGACCGAGCACGTGGAGGGCGATCCCTGCAAATTCGCCTTGTGGTCTGGGCGCACCCCATCCTCAGACAAT
AAAACAGTGCTGAAAGCCTCCAACATTGAAACCAAGCAGGAGTGGATCAAGAACATTCGAGAAGTGATTCAAGAAAGGATCATTCACCTGAAAGGAGCTT
TAAAGGAGCCACTTCAGCTCCCCAAAACACCAGCCAAACAGAGGAACAATAGTAAGAGGGATGGAGTGGAGGATATTGACAGCCAGGGGGATGGGAGCA
GCCAACCAGACACCATCTCCATTGCTTCTAGGACCTCTCAGAACACAGTGGACAGTGACAAGGATGGCAACCTTGTTCCTCGGTGGCACCTGGGACCTGGA
GATCCTTTCTCCACTTACGTTTAG

>Isoform 427

ATGTCTGTTTCCTTCCACACACACACCAAAGAGTTGTGGACATGGATGGAAGACCTTCAGAAGGAGATGTTGGAGGATGTCTGTGCAGATTCTGTGGAT
GCAGTCCAGGAACTGATCAAGCAGTTCCAGCAGCAGCAGACCGCCACTCTAGATGCCACACTCAATGTCATCAAGGAAGGCGAAGACCTTATCCAGCAG
CTCAGGTCAGCGCCTCCCTCCCTCGGGGAGCCCAGCGAGGCCAGGGACTCGGCTGTGTCCAACAACAAAACACCCCACAGCAGCTCCATCAGCCACATC
GAGTCGGTCCTGCAGCAGCTTGATGATGCCCAGGTGCAGATGGAGGAGCTGTTCCACGAGCGGAAGATCAAGCTGGACATCTTCCTGCAACTGCGCATC
TTTGAGCAGTACACCATCGAGGTGACAGCAGAGCTAGACGCCTGGAATGAAGACTTGCTTCGGCAGATGAATGACTTCAACACAGAGGACCTAACCCTG
GCAGAACAGCGGCTGCAGCGCCACACAGAACGGAAGCTAGCCATGAACAACATGACCTTTGAGGTTATCCAGCAGGGACAGGATCTGCACCAGTACAT
CACGGAGGTCCAGGCATCAGGAATTGAGTTGATCTGTGAAAAAGACATTGATCTGGCAGCCCAGGTGCAAGAGTTATTGGAATTTCTCCATGAGAAGCA
GCATGAATTGGAGCTCAATGCAGAGCAGACTCATAAGCGGCTAGAGCAGTGCCTCCAATTACGTCACCTCCAGGCTGAAGTCAAACAGGTTCTGGGATG
GATCCGCAATGGAGAGTCAATGCTCAACGCCAGCCTGGTCAATGCCAGCTCTTTGTCGGAAGCAGAGCAGCTGCAGCGGGAGCACGAGCAGTTCCAACT
GGCCATCGAGTCCCTCTTTCATGCCACTTCCTTGCAGAAGACGCACCAGAGTGCCCTGCAGGTACAGCAGAAAGCCGAGGTGCTGCTCCAGGCCGGCCA
CTACGATGCCGATGCCATCCGGGAATGTGCTGAGAAGGTGGCCCTCCACTGGCAGCAGCTCATGCTGAAGATGGAAGACCGGCTAAAATTGGTCAATGC
CTCTGTGGCCTTTTACAAAACTTCTGAACAGGTGTGTAGTGTCCTGGAGAGCTTAGAGCAAGAATACCGGAGAGATGAGGACTGGTGTGGTGGACGAG
ATAAGCTGGGGCCAGCAGCAGAGATCGACCATGTCATTCCCCTCATCAGCAAACATTTGGAACAAAAGGAGGCCTTTCTTAAGGCCTGCACCCTGGCTCG
GCGGAATGCTGAGGTGTTTCTCAAGTACATCCACAGGAACAACGTCAGCATGCCCAGTGTCGCCAGCCACACTCGGGGACCCGAGCAACAAGTGAAAGC
CATCCTGAGTGAGCTCCTGCAGAGGGAGAATCGCGTGCTGCATTTCTGGACCTTGAAGAAGCGGCGGTTAGACCAATGCCAGCAATATGTGGTGTTCGA
GCGCAGCGCTAAGCAGGCGCTTGACTGGATCCAAGAAACAGGTGAATTTTACCTCTCAACACATACCTCCACTGGAGAGACCACAGAGGAGACTCAGGA
ACTGCTGAAAGAATATGGGGAATTCAGGGTGCCTGCCAAGCAAACAAAGGAGAAGGTGAAGCTTCTGATTCAGCTGGCCGATAGCTTTGTGGAAAAAG
GCCACATTCATGCCACGGAGATAAGGAAATGGGTGACCACGGTGGACAAGCACTACAGAGATTTCTCCCTGAGGATGGGAAAGTACCGATACTCACTG
GAGAAAGCCCTAGGAGTCAACACAGAGGATAATAAGGACCTGGAGCTGGATATTATCCCAGCAAGCCTTTCGGATCGGGAGGTCAAGCTGCGGGACGC
CAACCACGAAGTCAATGAAGAGAAGCGGAAGTCAGCCCGGAAGAAAGAATTTATTATGGCTGAACTACTCCAGACAGAGAAGGCTTATGTAAGGGATT
TGCATGAGTGCTTAGAGACCTACCTGTGGGAAATGACCAGTGGTGTGGAGGAGATCCCCCCTGGGATCCTCAATAAAGAGCATATCATCTTTGGCAACA
TCCAAGAGATCTACGATTTCCATAACAACATCTTCCTCAAAGAGCTGGAGAAGTACGAGCAACTGCCTGAGGATGTGGGACACTGCTTTGTTACCTGGGC
AGACAAATTTCAGATGTATGTCACCTACTGTAAAAACAAGCCTGATTCCAACCAGCTTATCCTGGAGCATGCGGGCACCTTCTTTGATGAGATACAACAGC
GGCATGGTCTGGCCAACTCCATCTCTTCCTACCTAATTAAGCCTGTCCAAAGGATCACCAAATATCAACTGCTCCTGAAGGAACTTTTAACTTGCTGTGAA
GAAGGGAAAGGGGAGCTCAAGGATGGCCTGGAGGTGATGCTCAGTGTCCCAAAGAAAGCCAATGATGCCATGCATGTCAGCATGCTGGAAGGGTTCG
ACGAGAACCTGGATGTGCAGGGGGAGTTGATTCTCCAGGATGCCTTTCAAGTGTGGGACCCGAAGTCGCTGATCCGGAAGGGGCGGGAGCGGCACTTG
TTCCTCTTTGAGATCTCCTTGGTTTTTAGCAAGGAGATCAAAGATTCTTCAGGACACACGAAATATGTTTACAAGAACAAGCTACTGACCTCAGAGCTGGG
TGTGACCGAGCACGTGGAGGGCGATCCCTGCAAATTCGCCTTGTGGTCTGGGCGCACCCCATCCTCAGACAATAAAACAGTGCTGAAAGCCTCCAACATT
GAAACCAAGCAGGAGTGGATCAAGAACATTCGAGAAGTGATTCAAGAAAGGATCATTCACCTGAAAGGAGCTTTAAAGGAGCCACTTCAGCTCCCCAAA
ACACCAGCCAAACAGAGGAACAATAGTAAGAGGGATGGAGTGGAGGATATTGACAGCCAGGGGGATGGGAGCAGCCAACCAGACACCATCTCCATTG
CTTCTAGGACCTCTCAGAACACAGTGGACAGTGACAAGGATGGCAACCTTGTTCCTCGGTGGCACCTGGGACCTGGAGATCCTTTCTCCACTTACGTTTA
G

>Isoform 4061

ATGAACCCCCCTGAGGGAGCAGCGGAGGAAGGAGGAGCAGCAGACTCGGACGTGGACGCCTTTTTCCGGACAGGGTCTTTTCGGAATGATGGTTTGAAAGC
TTCTGATGTCCTTCCTATCCTAAAGGAAAAGGTGGCCTTCGTGTCTGGGGGTCGTGATAAGCGAGGCGGACCCATCCTGACCTTCCCTGCTCGCAGCAATCATGA
CAGAATAAGACAGGAAGACCTGCGGAAACTCGTGACGTATTTGGCCAGCGTGCCAAGTGAGGACGTGTGCAAACGTGGCTTCACTGTCATCATCGACATGCGG
GGCTCCAAGTGGGACCTCATCAAGCCCCTCCTCAAAACGCTGCAGGAAGCCTTTCCAGCTGAGATCCATGTGGCCCTCATCATTAAACCCGACAACTTCTGGCA
GAAACAGAAGACCAACTTTGGCAGCTCCAAATTCATCTTTGAGACGAGCATGGTATCTGTGGAGGGCCTCACAAAGCTGGTGGACCCCTCCCAGCTGACGGAG
GAGTTTGATGGCTCCCTGGACTACAACCATGAGGAGTGGATCGAACTGCGGCTCTCCCTGGAGGAGTTCTTCAACAGCGCCGTGCACCTGCTCTCGCGCCTCGA
GGACCTCCAGGAGATGCTAGCCCGGAAGGAGTTTCCTGTGGATGTGGAGGGCTCTCGGCGGCTCATTGACGAACACACACAGCTCAAGAAAAAGGTGCTGAA
GGCCCCTGTGGAGGAGCTGGACCGGGAGGGGCAGCGGCTGCTGCAGTGCATCCGCTGCAGCGACGGCTTCTCAGGACGCAACTGCATCCCGGGCAGTGCTG
ACTTCCAGAGCCTGGTGCCCAAGATCACCAGTCTCCTGGACAAGCTGCACTCCACCCGGCAGCACCTGCACCAGATGTGGCATGTGCGCAAGCTCAAGCTGGA
CCAGTGCTTTCAGCTGCGGCTCTTCGAGCAGGATGCTGAGAAGATGTTTGACTGGATAAGCCACAACAAGGAGTTATTCCTCCAGAGCCACACGGAGATCGGA
GTCAGCTACCAGTACGCCCTTGACCTCCAGACGCAGCACAATCACTTTGCCATGAACTCCATGAATGCCTATGTCAACATCAACCGCATCATGTCCGTGGCTTCCC
GCCTCTCTGAGGCCGGTCATTATGCCTCACAACAAATCAAGCAGATCTCCACCCAGCTGGACCAGGAGTGGAAGAGCTTCGCTGCTGCCCTGGATGAACGCAGC
ACCATCCTCGCCATGTCTGCTGTGTTCCACCAGAAGGCTGAGCAGTTCCTGTCGGGAGTGGATGCCTGGTGCAAGATGTGCAGTGAAGGTGGTCTGCCATCCGA
GATGCAAGACCTAGAGCTGGCAATCCACCACCACCAGACCTTGTATGAGCAGGTGACCCAAGCCTACACAGAGGTCAGCCAGGATGGCAAAGCACTACTTGAT
GTGCTGCAGCGGCCCCTGAGCCCTGGGAACTCCGAATCCCTCACGGCCACAGCCAACTACTCCAAGGCAGTGCACCAGGTGCTGGACGTGGTGCATGAGGTGT
TACATCACCAGCGACGGCTGGAGAGCATCTGGCAGCACCGCAAGGTGCGGCTCCACCAGCGGCTGCAGCTCTGCGTCTTCCAGCAGGATGTACAGCAGGTGTT
GGACTGGATTGAAAACCATGGTGAGGCCTTTCTCAGCAAACACACTGGAGTTGGGAAGTCCCTACATCGAGCCCGGGCCCTGCAGAAGAGGCATGATGACTTT
GAAGAGGTGGCTCAGAATACGTACACCAATGCGGACAAGCTCCTAGAAGCAGCAGAGCAGTTGGCTCAGACGGGGGAATGTGACCCCGAGGAGATCTACAAG
GCAGCTCGACACCTGGAGGTGCGCATCCAAGACTTCGTGCGCAGGGTGGAGCAGCGGAAGCTTCTCCTGGACATGTCTGTTTCCTTCCACACACACACCAAAG
AGTTGTGGACATGGATGGAAGACCTTCAGAAGGAGATGTTGGAGGATGTCTGTGCAGATTCTGTGGATGCAGTCCAGGAACTGATCAAGCAGTTCCAGCAGCA
GCAGACCGCCACTCTAGATGCCACACTCAATGTCATCAAGGAAGGCGAAGACCTTATCCAGCAGCTCAGGGACTCGGCTGTGTCCAACAACAAAACACCCCACA
GCAGCTCCATCAGCCACATCGAGTCGGTCCTGCAGCAGCTTGATGATGCCCAGGTGCAGATGGAGGAGCTGTTCCACGAGCGGAAGATCAAGCTGGACATCTT
CCTGCAACTGCGCATCTTTGAGCAGTACACCATCGAGGTGACAGCAGAGCTAGACGCCTGGAATGAAGACTTGCTTCGGCAGATGAATGACTTCAACACAGAG
GACCTAACCCTGGCAGAACAGCGGCTGCAGCGCCACACAGAACGGAAGCTAGCCATGAACAACATGACCTTTGAGGTTATCCAGCAGGGACAGGATCTGCACC
AGTACATCACGGAGGTCCAGGCATCAGGAATTGAGTTGATCTGTGAAAAAGACATTGATCTGGCAGCCCAGGTGCAAGAGTTATTGGAATTTCTCCATGAGAAG
CAGCATGAATTGGAGCTCAATGCAGAGCAGACTCATAAGCGGCTAGAGCAGTGCCTCCAATTACGTCACCTCCAGGCTGAAGTCAAACAGGTTCTGGGATGGAT
CCGCAATGGAGAGTCAATGCTCAACGCCAGCCTGGTCAATGCCAGCTCTTTGTCGGAAGCAGAGCAGCTGCAGCGGGAGCACGAGCAGTTCCAACTGGCCATC
GAGAAGACGCACCAGAGTGCCCTGCAGGTACAGCAGAAAGCCGAGGTGCTGCTCCAGGCCGGCCACTACGATGCCGATGCCATCCGGGAATGTGCTGAGAAG
GTGGCCCTCCACTGGCAGCAGCTCATGCTGAAGATGGAAGACCGGCTAAAATTGGTCAATGCCTCTGTGGCCTTTTACAAAACTTCTGAACAGGTGTGTAGTGT
CCTGGAGAGCTTAGAGCAAGAATACCGGAGAGATGAGGACTGGTGTGGTGGACGAGATAAGCTGGGGCCAGCAGCAGAGATCGACCATGTCATTCCCCTCATC
AGCAAACATTTGGAACAAAAGGAGGCCTTTCTTAAGGCCTGCACCCTGGCTCGGCGGAATGCTGAGGTGTTTCTCAAGTACATCCACAGGAACAACGTCAGCA
TGCCCAGTGTCGCCAGCCACACTCGGGGACCCGAGCAACAAGTGAAAGCCATCCTGAGTGAGCTCCTGCAGAGGGAGAATCGCGTGCTGCATTTCTGGACCTT
GAAGAAGCGGCGGTTAGACCAATGCCAGCAATATGTGGTGTTCGAGCGCAGCGCTAAGCAGGCGCTTGACTGGATCCAAGAAACAGGTGAATTTTACCTCTCA
ACACATACCTCCACTGGAGAGACCACAGAGGAGACTCAGGAACTGCTGAAAGAATATGGGGAATTCAGGGTGCCTGCCAAGCAAACAAAGGAGAAGGTGAA
GCTTCTGATTCAGCTGGCCGATAGCTTTGTGGAAAAAGGCCACATTCATGCCACGGAGATAAGGAAATGGGTGACCACGGTGGACAAGCACTACAGAGATTTCT
CCCTGAGGATGGGAAAGTACCGATACTCACTGGAGAAAGCCCTAGGAGTCAACACAGAGGATAATAAGGACCTGGAGCTGGATATTATCCCAGCAAGCCTTTCG
GATCGGGAGGTCAAGCTGCGGGACGCCAACCACGAAGTCAATGAAGAGAAGCGGAAGTCAGCCCGGAAGAAAGAATTTATTATGGCTGAACTACTCCAGACA
GAGAAGGCTTATGTAAGGGATTTGCATGAGTGCTTAGAGACCTACCTGTGGGAAATGACCAGTGGTGTGGAGGAGATCCCCCCTGGGATCCTCAATAAAGAGC
ATATCATCTTTGGCAACATCCAAGAGATCTACGATTTCCATAACAACATCTTCCTCAAAGAGCTGGAGAAGTACGAGCAACTGCCTGAGGATGTGGGACACTGCT
TTGTTACCTGGGCAGACAAATTTCAGATGTATGTCACCTACTGTAAAAACAAGCCTGATTCCAACCAGCTTATCCTGGAGCATGCGGGCACCTTCTTTGATGAGAT
ACAACAGCGGCATGGTCTGGCCAACTCCATCTCTTCCTACCTAATTAAGCCTGTCCAAAGGATCACCAAATATCAACTGCTCCTGAAGGAACTTTTAACTTGCTGT
GAAGAAGGGAAAGGGGAGCTCAAGGATGGCCTGGAGGTGATGCTCAGTGTCCCAAAGAAAGCCAATGATGCCATGCATGTCAGCATGCTGGAAGGGTTCGAC
GAGAACCTGGATGTGCAGGGGGAGTTGATTCTCCAGGATGCCTTTCAAGTGTGGGACCCGAAGTCGCTGATCCGGAAGGGGCGGGAGCGGCACTTGTTCCTC
TTTGAGATCTCCTTGGTTTTTAGCAAGGAGATCAAAGATTCTTCAGGACACACGAAATATGTTTACAAGAACAAGCTACTGACCTCAGAGCTGGGTGTGACCGA
GCACGTGGAGGGCGATCCCTGCAAATTCGCCTTGTGGTCTGGGCGCACCCCATCCTCAGACAATAAAACAGTGCTGAAAGCCTCCAACATTGAAACCAAGCAG
GAGTGGATCAAGAACATTCGAGAAGTGATTCAAGAAAGGATCATTCACCTGAAAGGAGCTTTAAAGGAGCCACTTCAGCTCCCCAAAACACCAGCCAAACAGA
GGAACAATAGTAAGAGGGATGGAGTGGAGGATATTGACAGCCAGGGGGATGGGAGCAGCCAACCAGACACCATCTCCATTGCTTCTAGGACCTCTCAGAACAC
AGTGGACAGTGACAAGCTCTCTGGTGGATGTGAGCTGACAGTGGTCCTCCAGGACTTCAGTGCGGGCCACAGCAGTGAGCTGACCATCCAGGTGGGGCAGAC
GGTAGAGCTGCTGGAGCGGCCCAGCGAGCGGCCTGGTTGGTGTCTGGTCCGTACCACCGAACGGAGCCCGCCCTTGGAGGGTCTGGTCCCCAGCAGCGCCCT
GTGCATCTCACACTCCCGAAGCAGCGTGGAGATGGACTGCTTCTTCCCCTTGGTGAAAGATGCATACTCTCATTCCTCAAGCGAGAATGGAGGCAAGTCCGAGT
CCGTGGCCAACCTGCAGGCCCAGCCCTCCCTGAACTCCATCCACAGTTCCCCGGGTCCCAAGCGCTCCACCAACACTCTTAAGAAGTGGCTGACGAGTCCTGTG
CGTCGGCTTAACAGCGGGAAGGCAGATGGAAACATCAAAAAGCAGAAGAAAGTTCGCGATGGTCGGAAGAGCTTTGACCTGGGATCTCCCAAGCCTGGGGAT
GAAACAACCCCTCAGGGAGACAGCGCTGATGAGAAGAGCAAGAAAGGTTGGGGTGAAGATGAGCCGGATGAAGAGTCACACACACCCCTCCCACCACCTATG
AAGATTTTTGACAACGACCCTACACAGGATGAAATGAGTCTAGAAGGAAGCTCATACCGGGGGAGCTTGAAAGACCCTGCAGGCTGCCTGAATGAGGGGATG
GCCCCACCCACACCTCCTAAAAACCCAGAAGAAGAACAGAAAGCCAAGGCCCTGAGAGGCAGGATGTTTGTCCTGAATGAGCTGGTACAGACAGAGAAAGAC
TATGTCAAGGATCTGGGCATTGTGGTGGAGGGCTTCATGAAGAGAATAGAAGAAAAGGGTGTCCCTGAGGATATGCGAGGAAAGGACAAAATCGTGTTTGGA
AATATTCATCAGATTTATGACTGGCATAAGGATTTTTTCCTGGCGGAACTGGAAAAGTGTATCCAGGAGCAAGACAGATTGGCACAGCTCTTTATTAAGCACGAG
CGGAAGCTGCACATCTACGTGTGGTATTGTCAGAATAAGCCGCGCTCAGAGTACATCGTTGCTGAGTATGACGCCTACTTTGAGGAGGTAAAACAGGAGATAAAT
CAGAGGCTGACACTGAGTGACTTCCTCATCAAGCCCATTCAGAGAATAACAAAATACCAGTTGCTCCTCAAGGACTTCCTGAGATACAGTGAGAAGGCTGGTTT
GGAGTGTTCAGATATTGAGAAAGCAGTGGAGTTAATGTGCCTTGTTCCCAAACGCTGCAATGACATGATGAATCTAGGACGTCTGCAGGGCTTTGAGGGCACTC
TGACTGCTCAGGGGAAGCTGCTGCAGCAGGACACATTCTATGTGATCGAGCTGGATGCAGGCATGCAGTCCCGGACCAAAGAGAGGCGCGTGTTCCTCTTCGA
GCAGATTGTCATCTTCAGTGAACTGCTCAGGAAGGGATCCCTCACCCCTGGCTACATGTTCAAAAGGAGCATCAAGATGAATTACTTGGTCCTGGAGGAGAATG
TGGACAATGATCCCTGCAAGTTTGCACTCATGAACAGAGAGACTTCTGAGAGGGTTGTTCTGCAAGCCGCCAACGCTGACATCCAGCAGGCCTGGGTGCAGGA
CATCAATCAAGTCTTAGAAACACAGCGAGACTTTTTGAATGCACTGCAATCGCCCATTGAGTATCAACGGAAAGAAAGGAGCACAGCTGTGATGAGGTCTCAAC
CTGCCAGGCTTCCCCAAGCCAGCCCCAGGCCCTACTCCTCTGTTCCTGCGGGCTCAGAGAAGCCCCCAAAGGGCTCCAGCTATAACCCACCTCTGCCTCCCCTGA
AGATATCTACCTCCAATGGCAGTCCAGGGTTTGAATACCACCAGCCTGGGGACAAGTTCGAAGCCAGCAAGAACGACCTGGGAGGCTGCAATGGGACCTCGTC
CATGGCCGTGATCAAAGATTACTATGCACTGAAGGAGAATGAAATCTGTGTGAGCCAAGGTGAGGTGGTCCAGGTCCTCGCCGTCAACCAGCAGAACATGTGTC
TGGTGTACCAGCCTGCCAGCGACCATTCCCCCGCCGCCGAGGGCTGGGTCCCAGGCAGCATCCTGGCGCCCCTCACCAAAGCCACAGCAGCAGAAAGTAGTGA
CGGGAGCATCAAGAAGTCATGTTCATGGCATACTCTACGCATGAGAAAGCGGGCGGAAGTGGAGAACACGGGTAAAAATGAAGCCACAGGGCCTCGTAAACC
CAAGGATATTCTGGGCAACAAAGTCTCTGTTAAAGAGACGAACAGTTCCGAGGAATCAGAGTGTGATGATCTTGACCCTAATACTAGCATGGAGATCTTAAATCC
AAATTTCATCCAAGAAGTGGCCCCAGAATTCCTTGTGCCCTTGGTGGATGTGACCTGCTTGCTTGGGGACACAGTGATACTGCAGTGCAAAGTCTGTGGGCGG
CCAAAGCCCACCATCACTTGGAAGGGTCCAGACCAGAACATCCTTGACACTGATAACAGCTCAGCCACATACACGGTCTCCTCTTGTGATTCTGGAGAAATCA
CCCTGAAGATCTGTAATCTGATGCCCCAAGACAGTGGGATTTATACCTGCATAGCAACAAATGACCACGGGACCACATCAACGTCTGCAACAGTCAAAGTGC
AAGGTGTTCCAGCAGCCCCTAACCGCCCCATTGCCCAGGAGAGAAGCTGCACCTCCGTGATTCTCCGCTGGCTGCCCCCCTCCAGCACAGGAAACTGCACTAT
TTCTGGTTACACTGTGGAGTACAGAGAGGAAGGTTCTCAGATCTGGCAGCAGTCAGTGGCTTCGACCTTGGACACTTACCTCGTCATCGAAGACCTTAGTCCC
GGGTGTCCTTATCAGTTCAGAGTCAGTGCCAGTAACCCCTGGGGAATCAGCCTTCCCAGCGAGCCCTCGGAGTTTGTGCGACTTCCAGAATATGATGCTGCTG
CTGATGGTGCCACCATTTCTTGGAAGGAAAATTTTGACTCAGCTTACACTGAGCTGAATGAAATTGGAAGAGGCCGTTTCTCTATAGTAAAGAAATGCATTCA
CAAAGCTACCCGCAAAGATGTGGCTGTGAAATTTGTTAGCAAAAAAATGAAGAAGAAAGAACAGGCTGCCCACGAGGCTGCCC

TGCTTCAGCACCTACAGCACCCCCAGTACATCACTCTCCATGACACCTATGAGTCCCCCACATCCTACATCCTGATCTTGGAACTGATGGATGATGGCCGGCTCT
TAGACTACCTTATGAATCATGATGAACTGATGGAGGAAAAAGTAGCTTTCTATATCCGAGACATCATGGAGGCTCTGCAGTACCTTCACAACTGCAGGGTTGC
ACATTTGGACATAAAGCCTGAAAACCTGCTCATTGACCTACGGATTCCAGTGCCTCGAGTGAAGCTCATTGACTTGGAGGATGCTGTCCAGATCTCGGGTCACT
TCCACATTCACCACCTGCTGGGGAACCCTGAGTTTGCTGCCCCAGAAGTCATTCAAGGCATCCCCGTCTCCCTGGGGACAGACATCTGGAGCATCGGGGTTCTG
ACATATGTCATGCTGAGTGGGGTCTCCCCCTTCTTGGATGAGAGCAAAGAGGAGACATGTATCAACGTATGCAGGGTGGATTTCAGCTTCCCCCATGAATACTT
CTGTGGTGTGAGCAATGCTGCCAGAGATTTCATCAATGTGATCTTACAGGAAGATTTTCGGAGGCGGCCCACAGCAGCCACATGCTTGCAGCATCCATGGCTG
CAGCCCCATAATGGCAGCTACTCTAAGATCCCCCTGGACACCTCCCGCCTAGCATGCTTCATAGAACGTCGCAAGCACCAGAATGATGTGCGGCCTATTCCCAA
TGTCAAGAGCTACATTGTCAACCGGGTGAACCAAGGGACGTAG

>Isoform 4031

ATGAACCCCCCTGAGGGAGCAGCGGAGGAAGGAGGAGCAGCAGACTCGGACGTGGACGCCTTTTTCCGGACAGGGTCTTTTCGGAATGATGGTTTGAAAGC
TTCTGATGTCCTTCCTATCCTAAAGGAAAAGGTGGCCTTCGTGTCTGGGGGTCGTGATAAGCGAGGCGGACCCATCCTGACCTTCCCTGCTCGCAGCAATCATG
ACAGAATAAGACAGGAAGACCTGCGGAAACTCGTGACGTATTTGGCCAGCGTGCCAAGTGAGGACGTGTGCAAACGTGGCTTCACTGTCATCATCGACATGC
GGGGCTCCAAGTGGGACCTCATCAAGCCCCTCCTCAAAACGCTGCAGGAAGCCTTTCCAGCTGAGATCCATGTGGCCCTCATCATTAAACCCGACAACTTCTGG
CAGAAACAGAAGACCAACTTTGGCAGCTCCAAATTCATCTTTGAGACGAGCATGGTATCTGTGGAGGGCCTCACAAAGCTGGTGGACCCCTCCCAGCTGACGG
AGGAGTTTGATGGCTCCCTGGACTACAACCATGAGGAGTGGATCGAACTGCGGCTCTCCCTGGAGGAGTTCTTCAACAGCGCCGTGCACCTGCTCTCGCGCCT
CGAGGACCTCCAGGAGATGCTAGCCCGGAAGGAGTTTCCTGTGGATGTGGAGGGCTCTCGGCGGCTCATTGACGAACACACACAGCTCAAGAAAAAGGTGCT
GAAGGCCCCTGTGGAGGAGCTGGACCGGGAGGGGCAGCGGCTGCTGCAGTGCATCCGCTGCAGCGACGGCTTCTCAGGACGCAACTGCATCCCGGGCAGTG
CTGACTTCCAGAGCCTGGTGCCCAAGATCACCAGTCTCCTGGACAAGCTGCACTCCACCCGGCAGCACCTGCACCAGATGTGGCATGTGCGCAAGCTCAAGCT
GGACCAGTGCTTTCAGCTGCGGCTCTTCGAGCAGGATGCTGAGAAGATGTTTGACTGGATAAGCCACAACAAGGAGTTATTCCTCCAGAGCCACACGGAGATC
GGAGTCAGCTACCAGTACGCCCTTGACCTCCAGACGCAGCACAATCACTTTGCCATGAACTCCATGAATGCCTATGTCAACATCAACCGCATCATGTCCGTGGC
TTCCCGCCTCTCTGAGGCCGGTCATTATGCCTCACAACAAATCAAGCAGATCTCCACCCAGCTGGACCAGGAGTGGAAGAGCTTCGCTGCTGCCCTGGATGAA
CGCAGCACCATCCTCGCCATGTCTGCTGTGTTCCACCAGAAGGCTGAGCAGTTCCTGTCGGGAGTGGATGCCTGGTGCAAGATGTGCAGTGAAGGTGGTCTGC
CATCCGAGATGCAAGACCTAGAGCTGGCAATCCACCACCACCAGACCTTGTATGAGCAGGTGACCCAAGCCTACACAGAGGTCAGCCAGGATGGCAAAGCAC
TACTTGATGTGCTGCAGCGGCCCCTGAGCCCTGGGAACTCCGAATCCCTCACGGCCACAGCCAACTACTCCAAGGCAGTGCACCAGGTGCTGGACGTGGTGCA
TGAGGTGTTACATCACCAGCGACGGCTGGAGAGCATCTGGCAGCACCGCAAGGTGCGGCTCCACCAGCGGCTGCAGCTCTGCGTCTTCCAGCAGGATGTACA
GCAGGTGTTGGACTGGATTGAAAACCATGGTGAGGCCTTTCTCAGCAAACACACTGGAGTTGGGAAGTCCCTACATCGAGCCCGGGCCCTGCAGAAGAGGCA
TGATGACTTTGAAGAGGTGGCTCAGAATACGTACACCAATGCGGACAAGCTCCTAGAAGCAGCAGAGCAGTTGGCTCAGACGGGGGAATGTGACCCCGAGG
AGATCTACAAGGCAGCTCGACACCTGGAGGTGCGCATCCAAGACTTCGTGCGCAGGGTGGAGCAGCGGAAGCTTCTCCTGGACATGTCTGTTTCCTTCCACAC
ACACACCAAAGAGTTGTGGACATGGATGGAAGACCTTCAGAAGGAGATGTTGGAGGATGTCTGTGCAGATTCTGTGGATGCAGTCCAGGAACTGATCAAGCA
GTTCCAGCAGCAGCAGACCGCCACTCTAGATGCCACACTCAATGTCATCAAGGAAGGCGAAGACCTTATCCAGCAGCTCAGGGACTCGGCTGTGTCCAACAAC
AAAACACCCCACAGCAGCTCCATCAGCCACATCGAGTCGGTCCTGCAGCAGCTTGATGATGCCCAGGTGCAGATGGAGGAGCTGTTCCACGAGCGGAAGATC
AAGCTGGACATCTTCCTGCAACTGCGCATCTTTGAGCAGTACACCATCGAGGTGACAGCAGAGCTAGACGCCTGGAATGAAGACTTGCTTCGGCAGATGAATG
ACTTCAACACAGAGGACCTAACCCTGGCAGAACAGCGGCTGCAGCGCCACACAGAACGGAAGCTAGCCATGAACAACATGACCTTTGAGGTTATCCAGCAGG
GACAGGATCTGCACCAGTACATCACGGAGGTCCAGGCATCAGGAATTGAGTTGATCTGTGAAAAAGACATTGATCTGGCAGCCCAGGTGCAAGAGTTATTGG
AATTTCTCCATGAGAAGCAGCATGAATTGGAGCTCAATGCAGAGCAGACTCATAAGCGGCTAGAGCAGTGCCTCCAATTACGTCACCTCCAGGCTGAAGTCAA
ACAGGTTCTGGGATGGATCCGCAATGGAGAGTCAATGCTCAACGCCAGCCTGGTCAATGCCAGCTCTTTGTCGGAAGCAGAGCAGCTGCAGCGGGAGCACGA
GCAGTTCCAACTGGCCATCGAGAAGACGCACCAGAGTGCCCTGCAGGTACAGCAGAAAGCCGAGGTGCTGCTCCAGGCCGGCCACTACGATGCCGATGCCAT
CCGGGAATGTGCTGAGAAGGTGGCCCTCCACTGGCAGCAGCTCATGCTGAAGATGGAAGACCGGCTAAAATTGGTCAATGCCTCTGTGGCCTTTTACAAAACT
TCTGAACAGGTGTGTAGTGTCCTGGAGAGCTTAGAGCAAGAATACCGGAGAGATGAGGACTGGTGTGGTGGACGAGATAAGCTGGGGCCAGCAGCAGAGA
TCGACCATGTCATTCCCCTCATCAGCAAACATTTGGAACAAAAGGAGGCCTTTCTTAAGGCCTGCACCCTGGCTCGGCGGAATGCTGAGGTGTTTCTCAAGTAC
ATCCACAGGAACAACGTCAGCATGCCCAGTGTCGCCAGCCACACTCGGGGACCCGAGCAACAAGTGAAAGCCATCCTGAGTGAGCTCCTGCAGAGGGAGAAT
CGCGTGCTGCATTTCTGGACCTTGAAGAAGCGGCGGTTAGACCAATGCCAGCAATATGTGGTGTTCGAGCGCAGCGCTAAGCAGGCGCTTGACTGGATCCAA
GAAACAGGTGAATTTTACCTCTCAACACATACCTCCACTGGAGAGACCACAGAGGAGACTCAGGAACTGCTGAAAGAATATGGGGAATTCAGGGTGCCTGCC
AAGCAAACAAAGGAGAAGGTGAAGCTTCTGATTCAGCTGGCCGATAGCTTTGTGGAAAAAGGCCACATTCATGCCACGGAGATAAGGAAATGGGTGACCAC
GGTGGACAAGCACTACAGAGATTTCTCCCTGAGGATGGGAAAGTACCGATACTCACTGGAGAAAGCCCTAGGAGTCAACACAGAGGATAATAAGGACCTGG
AGCTGGATATTATCCCAGCAAGCCTTTCGGATCGGGAGGTCAAGCTGCGGGACGCCAACCACGAAGTCAATGAAGAGAAGCGGAAGTCAGCCCGGAAGAAA
GAATTTATTATGGCTGAACTACTCCAGACAGAGAAGGCTTATGTAAGGGATTTGCATGAGTGCTTAGAGACCTACCTGTGGGAAATGACCAGTGGTGTGGAG
GAGATCCCCCCTGGGATCCTCAATAAAGAGCATATCATCTTTGGCAACATCCAAGAGATCTACGATTTCCATAACAACATCTTCCTCAAAGAGCTGGAGAAGTA
CGAGCAACTGCCTGAGGATGTGGGACACTGCTTTGTTACCTGGGCAGACAAATTTCAGATGTATGTCACCTACTGTAAAAACAAGCCTGATTCCAACCAGCTTA
TCCTGGAGCATGCGGGCACCTTCTTTGATGAGATACAACAGCGGCATGGTCTGGCCAACTCCATCTCTTCCTACCTAATTAAGCCTGTCCAAAGGATCACCAAA
TATCAACTGCTCCTGAAGGAACTTTTAACTTGCTGTGAAGAAGGGAAAGGGGAGCTCAAGGATGGCCTGGAGGTGATGCTCAGTGTCCCAAAGAAAGCCAAT
GATGCCATGCATGTCAGCATGCTGGAAGGGTTCGACGAGAACCTGGATGTGCAGGGGGAGTTGATTCTCCAGGATGCCTTTCAAGTGTGGGACCCGAAGTCG
CTGATCCGGAAGGGGCGGGAGCGGCACTTGTTCCTCTTTGAGATCTCCTTGGTTTTTAGCAAGGAGATCAAAGATTCTTCAGGACACACGAAATATGTTTACAA
GAACAAGCTACTGACCTCAGAGCTGGGTGTGACCGAGCACGTGGAGGGCGATCCCTGCAAATTCGCCTTGTGGTCTGGGCGCACCCCATCCTCAGACAATAA
AACAGTGCTGAAAGCCTCCAACATTGAAACCAAGCAGGAGTGGATCAAGAACATTCGAGAAGTGATTCAAGAAAGGATCATTCACCTGAAAGGAGCTTTAAA
GGAGCCACTTCAGCTCCCCAAAACACCAGCCAAACAGAGGAACAATAGTAAGAGGGATGGAGTGGAGGATATTGACAGCCAGGGGGATGGGAGCAGCCAA
CCAGACACCATCTCCATTGCTTCTAGGACCTCTCAGAACACAGTGGACAGTGACAAGCTCTCTGGTGGATGTGAGCTGACAGTGGTCCTCCAGGACTTCAGTGC
GGGCCACAGCAGTGAGCTGACCATCCAGGTGGGGCAGACGGTAGAGCTGCTGGAGCGGCCCAGCGAGCGGCCTGGTTGGTGTCTGGTCCGTACCACCGAAC
GGAGCCCGCCCTTGGAGGGTCTGGTCCCCAGCAGCGCCCTGTGCATCTCACACTCCCGAAGCAGCGTGGAGATGGACTGCTTCTTCCCCTTGGTGAAAGATGC
ATACTCTCATTCCTCAAGCGAGAATGGAGGCAAGTCCGAGTCCGTGGCCAACCTGCAGGCCCAGCCCTCCCTGAACTCCATCCACAGTTCCCCGGGTCCCAAGC
GCTCCACCAACACTCTTAAGAAGTGGCTGACGAGTCCTGTGCGTCGGCTTAACAGCGGGAAGGCAGATGGAAACATCAAAAAGCAGAAGAAAGTTCGCGATG
GTCGGAAGAGCTTTGACCTGGGATCTCCCAAGCCTGGGGATGAAACAACCCCTCAGGGAGACAGCGCTGATGAGAAGAGCAAGAAAGGTTGGGGTGAAGAT
GAGCCGGATGAAGAGTCACACACACCCCTCCCACCACCTATGAAGATTTTTGACAACGACCCTACACAGGATGAAATGAGTCTAGAAGGAAGCTCATACCGGG
GGAGCTTGAAAGACCCTGCAGGCTGCCTGAATGAGGGGATGGCCCCACCCACACCTCCTAAAAACCCAGAAGAAGAACAGAAAGCCAAGGCCCTGAGAGGC
AGGATGTTTGTCCTGAATGAGCTGGTACAGACAGAGAAAGACTATGTCAAGGATCTGGGCATTGTGGTGGAGGGCTTCATGAAGAGAATAGAAGAAAAGGG
TGTCCCTGAGGATATGCGAGGAAAGGACAAAATCGTGTTTGGAAATATTCATCAGATTTATGACTGGCATAAGGATTTTTTCCTGGCGGAACTGGAAAAGTGT
ATCCAGGAGCAAGACAGATTGGCACAGCTCTTTATTAAGCACGAGCGGAAGCTGCACATCTACGTGTGGTATTGTCAGAATAAGCCGCGCTCAGAGTACATCG
TTGCTGAGTATGACGCCTACTTTGAGGAGGTAAAACAGGAGATAAATCAGAGGCTGACACTGAGTGACTTCCTCATCAAGCCCATTCAGAGAATAACAAAATA
CCAGTTGCTCCTCAAGGACTTCCTGAGATACAGTGAGAAGGCTGGTTTGGAGTGTTCAGATATTGAGAAAGCAGTGGAGTTAATGTGCCTTGTTCCCAAACGC
TGCAATGACATGATGAATCTAGGACGTCTGCAGGGCTTTGAGGGCACTCTGACTGCTCAGGGGAAGCTGCTGCAGCAGGACACATTCTATGTGATCGAGCTG
GATGCAGGCATGCAGTCCCGGACCAAAGAGAGGCGCGTGTTCCTCTTCGAGCAGATTGTCATCTTCAGTGAACTGCTCAGGAAGGGATCCCTCACCCCTGGCT
ACATGTTCAAAAGGAGCATCAAGATGAATTACTTGGTCCTGGAGGAGAATGTGGACAATGATCCCTGCAAGTTTGCACTCATGAACAGAGAGACTTCTGAGA
GGGTTGTTCTGCAAGCCGCCAACGCTGACATCCAGCAGGCCTGGGTGCAGGACATCAATCAAGTCTTAGAAACACAGCGAGACTTTTTGAATGCACTGCAATC
GCCCATTGAGTATCAACGGAAAGAAAGGAGCACAGCTGTGATGAGGTCTCAACCTGCCAGGCTTCCCCAAGCCAGCCCCAGGCCCTACTCCTCTGTTCCTGCG
GGCTCAGAGAAGCCCCCAAAGGGCTCCAGCTATAACCCACCTCTGCCTCCCCTGAAGATATCTACCTCCAATGGCAGTCCAGGGTTTGAATACCACCAGCCTGG
GGACAAGTTCGAAGCCAGCAAGAACGACCTGGGAGGCTGCAATGGGACCTCGTCCATGGCCGTGATCAAAGATTACTATGCACTGAAGGAGAATGAAATCTG
TGTGAGCCAAGGTGAGGTGGTCCAGGTCCTCGCCGTCAACCAGCAGAACATGTGTCTGGTGTACCAGCCTGCCAGCGACCATTCCCCCGCCGCCGAGGGCTG
GGTCCCAGGCAGCATCCTGGCGCCCCTCACCAAAGCCACAGCAGCAGAAAGTAGTGACGGGAGCATCAAGTAA

>Isoform 838

ATGAACCCCCCTGAGGGAGCAGCGGAGGAAGGAGGAGCAGCAGACTCGGACGTGGACGCCTTTTTCCGGACAGGGTCTTTTCGGAATGATGGTTTGAAAGC
TTCTGATGTCCTTCCTATCCTAAAGGAAAAGGTGGCCTTCGTGTCTGGGGGTCGTGATAAGCGAGGCGGACCCATCCTGACCTTCCCTGCTCGCAGCAATCAT
GACAGAATAAGACAGGAAGACCTGCGGAAACTCGTGACGTATTTGGCCAGCGTGCCAAGTGAGGACGTGTGCAAACGTGGCTTCACTGTCATCATCGACATG
CGGGGCTCCAAGTGGGACCTCATCAAGCCCCTCCTCAAAACGCTGCAGGAAGCCTTTCCAGCTGAGATCCATGTGGCCCTCATCATTAAACCCGACAACTTCT
GGCAGAAACAGAAGACCAACTTTGGCAGCTCCAAATTCATCTTTGAGACGAGCATGGTATCTGTGGAGGGCCTCACAAAGCTGGTGGACCCCTCCCAGCTGA
CGGAGGAGTTTGATGGCTCCCTGGACTACAACCATGAGGAGTGGATCGAACTGCGGCTCTCCCTGGAGGAGTTCTTCAACAGCGCCGTGCACCTGCTCTCGC
GCCTCGAGGACCTCCAGGAGATGCTAGCCCGGAAGGAGTTTCCTGTGGATGTGGAGGGCTCTCGGCGGCTCATTGACGAACACACACAGCTCAAGAAAAAG
GTGCTGAAGGCCCCTGTGGAGGAGCTGGACCGGGAGGGGCAGCGGCTGCTGCAGTGCATCCGCTGCAGCGACGGCTTCTCAGGACGCAACTGCATCCCGG
GCAGTGCTGACTTCCAGAGCCTGGTGCCCAAGATCACCAGTCTCCTGGACAAGCTGCACTCCACCCGGCAGCACCTGCACCAGATGTGGCATGTGCGCAAGC
TCAAGCTGGACCAGTGCTTTCAGCTGCGGCTCTTCGAGCAGGATGCTGAGAAGATGTTTGACTGGATAAGCCACAACAAGGAGTTATTCCTCCAGAGCCACA
CGGAGATCGGAGTCAGCTACCAGTACGCCCTTGACCTCCAGACGCAGCACAATCACTTTGCCATGAACTCCATGAATGCCTATGTCAACATCAACCGCATCAT
GTCCGTGGCTTCCCGCCTCTCTGAGGCCGGTCATTATGCCTCACAACAAATCAAGCAGATCTCCACCCAGCTGGACCAGGAGTGGAAGAGCTTCGCTGCTGCC
CTGGATGAACGCAGCACCATCCTCGCCATGTCTGCTGTGTTCCACCAGAAGGCTGAGCAGTTCCTGTCGGGAGTGGATGCCTGGTGCAAGATGTGCAGTGAA
GGTGGTCTGCCATCCGAGATGCAAGACCTAGAGCTGGCAATCCACCACCACCAGACCTTGTATGAGCAGGTGACCCAAGCCTACACAGAGGTCAGCCAGGAT
GGCAAAGCACTACTTGATGTGCTGCAGCGGCCCCTGAGCCCTGGGAACTCCGAATCCCTCACGGCCACAGCCAACTACTCCAAGGCAGTGCACCAGGTGCTG
GACGTGGTGCATGAGGTGTTACATCACCAGCGACGGCTGGAGAGCATCTGGCAGCACCGCAAGGTGCGGCTCCACCAGCGGCTGCAGCTCTGCGTCTTCCAG
CAGGATGTACAGCAGGTGTTGGACTGGATTGAAAACCATGGTGAGGCCTTTCTCAGCAAACACACTGGAGTTGGGAAGTCCCTACATCGAGCCCGGGCCCTG
CAGAAGAGGCATGATGACTTTGAAGAGGTGGCTCAGAATACGTACACCAATGCGGACAAGCTCCTAGAAGCAGCAGAGCAGTTGGCTCAGACGGGGGAAT
GTGACCCCGAGGAGATCTACAAGGCAGCTCGACACCTGGAGGTGCGCATCCAAGACTTCGTGCGCAGGGTGGAGCAGCGGAAGCTTCTCCTGGACATGTCT
GTTTCCTTCCACACACACACCAAAGAGTTGTGGACATGGATGGAAGACCTTCAGAAGGAGATGTTGGAGGATGTCTGTGCAGATTCTGTGGATGCAGTCCAG
GAACTGATCAAGCAGTTCCAGCAGCAGCAGACCGCCACTCTAGATGCCACACTCAATGTCATCAAGGAAGGCGAAGACCTTATCCAGCAGCTCAGGTCAGCG
CCTCCCTCCCTCGGGGAGCCCAGCGAGGCCAGGGACTCGGCTGTGTCCAACAACAAAACACCCCACAGCAGCTCCATCAGCCACATCGAGTCGGTCCTGCAG
CAGCTTGATGATGCCCAGGTGCAGATGGAGGAGCTGTTCCACGAGCGGAAGATCAAGCTGGACATCTTCCTGCAACTGCGCATCTTTGAGCAGTACACCATC
GAGGTGACAGCAGAGCTAGACGCCTGGAATGAAGACTTGCTTCGGCAGATGAATGACTTCAACACAGAGGACCTAACCCTGGCAGAACAGCGGCTGCAGCG
CCACACAGAACGGAAGCTAGCCATGAACAACATGACCTTTGAGGTTATCCAGCAGGGACAGGATCTGCACCAGTACATCACGGAGGTCCAGGCATCAGGAAT
TGAGTTGATCTGTGAAAAAGACATTGATCTGGCAGCCCAGGTGCAAGAGTTATTGGAATTTCTCCATGAGAAGCAGCATGAATTGGAGCTCAATGCAGAGCA
GACTCATAAGCGGCTAGAGCAGTGCCTCCAATTACGTCACCTCCAGGCTGAAGTCAAACAGGTTCTGGGATGGATCCGCAATGGAGAGTCAATGCTCAACGC
CAGCCTGGTCAATGCCAGCTCTTTGTCGGAAGCAGAGCAGCTGCAGCGGGAGCACGAGCAGTTCCAACTGGCCATCGAGAAGACGCACCAGAGTGCCCTGC
AGGTACAGCAGAAAGCCGAGGTGCTGCTCCAGGCCGGCCACTACGATGCCGATGCCATCCGGGAATGTGCTGAGAAGGTGGCCCTCCACTGGCAGCAGCTC
ATGCTGAAGATGGAAGACCGGCTAAAATTGGTCAATGCCTCTGTGGCCTTTTACAAAACTTCTGAACAGGTGTGTAGTGTCCTGGAGAGCTTAGAGCAAGAA
TACCGGAGAGATGAGGACTGGTGTGGTGGACGAGATAAGCTGGGGCCAGCAGCAGAGATCGACCATGTCATTCCCCTCATCAGCAAACATTTGGAACAAAA
GGAGGCCTTTCTTAAGGCCTGCACCCTGGCTCGGCGGAATGCTGAGGTGTTTCTCAAGTACATCCACAGGAACAACGTCAGCATGCCCAGTGTCGCCAGCCAC
ACTCGGGGACCCGAGCAACAAGTGAAAGCCATCCTGAGTGAGCTCCTGCAGAGGGAGAATCGCGTGCTGCATTTCTGGACCTTGAAGAAGCGGCGGTTAGA
CCAATGCCAGCAATATGTGGTGTTCGAGCGCAGCGCTAAGCAGGCGCTTGACTGGATCCAAGAAACAGGTGAATTTTACCTCTCAACACATACCTCCACTGGA
GAGACCACAGAGGAGACTCAGGAACTGCTGAAAGAATATGGGGAATTCAGGGTGCCTGCCAAGCAAACAAAGGAGAAGGTGAAGCTTCTGATTCAGCTGG
CCGATAGCTTTGTGGAAAAAGGCCACATTCATGCCACGGAGATAAGGAAATGGGTGACCACGGTGGACAAGCACTACAGAGATTTCTCCCTGAGGATGGGA
AAGTACCGATACTCACTGGAGAAAGCCCTAGGAGTCAACACAGAGGATAATAAGGACCTGGAGCTGGATATTATCCCAGCAAGCCTTTCGGATCGGGAGGTC
AAGCTGCGGGACGCCAACCACGAAGTCAATGAAGAGAAGCGGAAGTCAGCCCGGAAGAAAGAATTTATTATGGCTGAACTACTCCAGACAGAGAAGGCTTA
TGTAAGGGATTTGCATGAGTGCTTAGAGACCTACCTGTGGGAAATGACCAGTGGTGTGGAGGAGATCCCCCCTGGGATCCTCAATAAAGAGCATATCATCTT
TGGCAACATCCAAGAGATCTACGATTTCCATAACAACATCTTCCTCAAAGAGCTGGAGAAGTACGAGCAACTGCCTGAGGATGTGGGACACTGCTTTGTTACC
TGGGCAGACAAATTTCAGATGTATGTCACCTACTGTAAAAACAAGCCTGATTCCAACCAGCTTATCCTGGAGCATGCGGGCACCTTCTTTGATGAGATACAAC
AGCGGCATGGTCTGGCCAACTCCATCTCTTCCTACCTAATTAAGCCTGTCCAAAGGATCACCAAATATCAACTGCTCCTGAAGGAACTTTTAACTTGCTGTGAA
GAAGGGAAAGGGGAGCTCAAGGATGGCCTGGAGGTGATGCTCAGTGTCCCAAAGAAAGCCAATGATGCCATGCATGTCAGCATGCTGGAAGGGTTCGACG
AGAACCTGGATGTGCAGGGGGAGTTGATTCTCCAGGATGCCTTTCAAGTGTGGGACCCGAAGTCGCTGATCCGGAAGGGGCGGGAGCGGCACTTGTTCCTC
TTTGAGATCTCCTTGGTTTTTAGCAAGGAGATCAAAGATTCTTCAGGACACACGAAATATGTTTACAAGAACAAGCTACTGACCTCAGAGCTGGGTGTGACCG
AGCACGTGGAGGGCGATCCCTGCAAATTCGCCTTGTGGTCTGGGCGCACCCCATCCTCAGACAATAAAACAGTGCTGAAAGCCTCCAACATTGAAACCAAGC
AGGAGTGGATCAAGAACATTCGAGAAGTGATTCAAGAAAGGATCATTCACCTGAAAGGAGCTTTAAAGGAGCCACTTCAGCTCCCCAAAACACCAGCCAAAC
AGAGGAACAATAGTAAGAGGGATGGAGTGGAGGATATTGACAGCCAGGGGGATGGGAGCAGCCAACCAGACACCATCTCCATTGCTTCTAGGACCTCTCAG
AACACAGTGGACAGTGACAAGGATGGCAACCTTGTTCCTCGGTGGCACCTGGGACCTGGAGATCCTTTCTCCACTTACGTTTAG

>Isoform 1358

ATGTCTGTTTCCTTCCACACACACACCAAAGAGTTGTGGACATGGATGGAAGACCTTCAGAAGGAGATGTTGGAGGATGTCTGTGCAGATTCTGTGGATGCA
GTCCAGGAACTGATCAAGCAGTTCCAGCAGCAGCAGACCGCCACTCTAGATGCCACACTCAATGTCATCAAGGAAGGCGAAGACCTTATCCAGCAGCTCAGG
TCAGCGCCTCCCTCCCTCGGGGAGCCCAGCGAGGCCAGGGACTCGGCTGTGTCCAACAACAAAACACCCCACAGCAGCTCCATCAGCCACATCGAGTCGGTC
CTGCAGCAGCTTGATGATGCCCAGGTGCAGATGGAGGAGCTGTTCCACGAGCGGAAGATCAAGCTGGACATCTTCCTGCAACTGCGCATCTTTGAGCAGTAC
ACCATCGAGGTGACAGCAGAGCTAGACGCCTGGAATGAAGACTTGCTTCGGCAGATGAATGACTTCAACACAGAGGACCTAACCCTGGCAGAACAGCGGCT
GCAGCGCCACACAGAACGGAAGCTAGCCATGAACAACATGACCTTTGAGGTTATCCAGCAGGGACAGGATCTGCACCAGTACATCACGGAGGTCCAGGCAT
CAGGAATTGAGTTGATCTGTGAAAAAGACATTGATCTGGCAGCCCAGGTGCAAGAGTTATTGGAATTTCTCCATGAGAAGCAGCATGAATTGGAGCTCAATG
CAGAGCAGACTCATAAGCGGCTAGAGCAGTGCCTCCAATTACGTCACCTCCAGGCTGAAGTCAAACAGGTTCTGGGATGGATCCGCAATGGAGAGTCAATGC
TCAACGCCAGCCTGGTCAATGCCAGCTCTTTGTCGGAAGCAGAGCAGCTGCAGCGGGAGCACGAGCAGTTCCAACTGGCCATCGAGAAGACGCACCAGAGT
GCCCTGCAGGTACAGCAGAAAGCCGAGGTGCTGCTCCAGGCCGGCCACTACGATGCCGATGCCATCCGGGAATGTGCTGAGAAGGTGGCCCTCCACTGGCA
GCAGCTCATGCTGAAGATGGAAGACCGGCTAAAATTGGTCAATGCCTCTGTGGCCTTTTACAAAACTTCTGAACAGGTGTGTAGTGTCCTGGAGAGCTTAGA
GCAAGAATACCGGAGAGATGAGGACTGGTGTGGTGGACGAGATAAGCTGGGGCCAGCAGCAGAGATCGACCATGTCATTCCCCTCATCAGCAAACATTTGG
AACAAAAGGAGGCCTTTCTTAAGGCCTGCACCCTGGCTCGGCGGAATGCTGAGGTGTTTCTCAAGTACATCCACAGGAACAACGTCAGCATGCCCAGTGTCG
CCAGCCACACTCGGGGACCCGAGCAACAAGTGAAAGCCATCCTGAGTGAGCTCCTGCAGAGGGAGAATCGCGTGCTGCATTTCTGGACCTTGAAGAAGCGG
CGGTTAGACCAATGCCAGCAATATGTGGTGTTCGAGCGCAGCGCTAAGCAGGCGCTTGACTGGATCCAAGAAACAGGTGAATTTTACCTCTCAACACATACCT
CCACTGGAGAGACCACAGAGGAGACTCAGGAACTGCTGAAAGAATATGGGGAATTCAGGGTGCCTGCCAAGCAAACAAAGGAGAAGGTGAAGCTTCTGAT
TCAGCTGGCCGATAGCTTTGTGGAAAAAGGCCACATTCATGCCACGGAGATAAGGAAATGGGTGACCACGGTGGACAAGCACTACAGAGATTTCTCCCTGAG
GATGGGAAAGTACCGATACTCACTGGAGAAAGCCCTAGGAGTCAACACAGAGGATAATAAGGACCTGGAGCTGGATATTATCCCAGCAAGCCTTTCGGATC
GGGAGGTCAAGCTGCGGGACGCCAACCACGAAGTCAATGAAGAGAAGCGGAAGTCAGCCCGGAAGAAAGAATTTATTATGGCTGAACTACTCCAGACAGA
GAAGGCTTATGTAAGGGATTTGCATGAGTGCTTAGAGACCTACCTGTGGGAAATGACCAGTGGTGTGGAGGAGATCCCCCCTGGGATCCTCAATAAAGAGCA
TATCATCTTTGGCAACATCCAAGAGATCTACGATTTCCATAACAACATCTTCCTCAAAGAGCTGGAGAAGTACGAGCAACTGCCTGAGGATGTGGGACACTGC
TTTGTTACCTGGGCAGACAAATTTCAGATGTATGTCACCTACTGTAAAAACAAGCCTGATTCCAACCAGCTTATCCTGGAGCATGCGGGCACCTTCTTTGATGA
GATACAACAGCGGCATGGTCTGGCCAACTCCATCTCTTCCTACCTAATTAAGCCTGTCCAAAGGATCACCAAATATCAACTGCTCCTGAAGGAACTTTTAACTT
GCTGTGAAGAAGGGAAAGGGGAGCTCAAGGATGGCCTGGAGGTGATGCTCAGTGTCCCAAAGAAAGCCAATGATGCCATGCATGTCAGCATGCTGGAAGG
GTTCGACGAGAACCTGGATGTGCAGGGGGAGTTGATTCTCCAGGATGCCTTTCAAGTGTGGGACCCGAAGTCGCTGATCCGGAAGGGGCGGGAGCGGCACT
TGTTCCTCTTTGAGATCTCCTTGGTTTTTAGCAAGGAGATCAAAGATTCTTCAGGACACACGAAATATGTTTACAAGAACAAGCTACTGACCTCAGAGCTGGGT

GTGACCGAGCACGTGGAGGGCGATCCCTGCAAATTCGCCTTGTGGTCTGGGCGCACCCCATCCTCAGACAATAAAACAGTGCTGAAAGCCTCCAACATTGA
AACCAAGCAGGAGTGGATCAAGAACATTCGAGAAGTGATTCAAGAAAGGATCATTCACCTGAAAGGAGCTTTAAAGGAGCCACTTCAGCTCCCCAAAACAC
CAGCCAAACAGAGGAACAATAGTAAGAGGGATGGAGTGGAGGATATTGACAGCCAGGGGGATGGGAGCAGCCAACCAGACACCATCTCCATTGCTTCTA
GGACCTCTCAGAACACAGTGGACAGTGACAAGGATGGCAACCTTGTTCCTCGGTGGCACCTGGGACCTGGAGATCCTTTCTCCACTTACGTTTAG

isoform 515 ∆Ex37

ATGAACCCCCCTGAGGGAGCAGCGGAGGAAGGAGGAGCAGCAGACTCGGACGTGGACGCCTTTTTCCGGACAGGGTCTTTTCGGAATGATGGTTTGAAAG
CTTCTGATGTCCTTCCTATCCTAAAGGAAAAGGTGGCCTTCGTGTCTGGGGGTCGTGATAAGCGAGGCGGACCCATCCTGACCTTCCCTGCTCGCAGCAATC
ATGACAGAATAAGACAGGAAGACCTGCGGAAACTCGTGACGTATTTGGCCAGCGTGCCAAGTGAGGACGTGTGCAAACGTGGCTTCACTGTCATCATCGAC
ATGCGGGGCTCCAAGTGGGACCTCATCAAGCCCCTCCTCAAAACGCTGCAGGAAGCCTTTCCAGCTGAGATCCATGTGGCCCTCATCATTAAACCCGACAAC
TTCTGGCAGAAACAGAAGACCAACTTTGGCAGCTCCAAATTCATCTTTGAGACGAGCATGGTATCTGTGGAGGGCCTCACAAAGCTGGTGGACCCCTCCCAG
CTGACGGAGGAGTTTGATGGCTCCCTGGACTACAACCATGAGGAGTGGATCGAACTGCGGCTCTCCCTGGAGGAGTTCTTCAACAGCGCCGTGCACCTGCT
CTCGCGCCTCGAGGACCTCCAGGAGATGCTAGCCCGGAAGGAGTTTCCTGTGGATGTGGAGGGCTCTCGGCGGCTCATTGACGAACACACACAGCTCAAGA
AAAAGGTGCTGAAGGCCCCTGTGGAGGAGCTGGACCGGGAGGGGCAGCGGCTGCTGCAGTGCATCCGCTGCAGCGACGGCTTCTCAGGACGCAACTGCA
TCCCGGGCAGTGCTGACTTCCAGAGCCTGGTGCCCAAGATCACCAGTCTCCTGGACAAGCTGCACTCCACCCGGCAGCACCTGCACCAGATGTGGCATGTGC
GCAAGCTCAAGCTGGACCAGTGCTTTCAGCTGCGGCTCTTCGAGCAGGATGCTGAGAAGATGTTTGACTGGATAAGCCACAACAAGGAGTTATTCCTCCAG
AGCCACACGGAGATCGGAGTCAGCTACCAGTACGCCCTTGACCTCCAGACGCAGCACAATCACTTTGCCATGAACTCCATGAATGCCTATGTCAACATCAAC
CGCATCATGTCCGTGGCTTCCCGCCTCTCTGAGGCCGGTCATTATGCCTCACAACAAATCAAGCAGATCTCCACCCAGCTGGACCAGGAGTGGAAGAGCTTC
GCTGCTGCCCTGGATGAACGCAGCACCATCCTCGCCATGTCTGCTGTGTTCCACCAGAAGGCTGAGCAGTTCCTGTCGGGAGTGGATGCCTGGTGCAAGAT
GTGCAGTGAAGGTGGTCTGCCATCCGAGATGCAAGACCTAGAGCTGGCAATCCACCACCACCAGACCTTGTATGAGCAGGTGACCCAAGCCTACACAGAGG
TCAGCCAGGATGGCAAAGCACTACTTGATGTGCTGCAGCGGCCCCTGAGCCCTGGGAACTCCGAATCCCTCACGGCCACAGCCAACTACTCCAAGGCAGTG
CACCAGGTGCTGGACGTGGTGCATGAGGTGTTACATCACCAGCGACGGCTGGAGAGCATCTGGCAGCACCGCAAGGTGCGGCTCCACCAGCGGCTGCAGC
TCTGCGTCTTCCAGCAGGATGTACAGCAGGTGTTGGACTGGATTGAAAACCATGGTGAGGCCTTTCTCAGCAAACACACTGGAGTTGGGAAGTCCCTACATC
GAGCCCGGGCCCTGCAGAAGAGGCATGATGACTTTGAAGAGGTGGCTCAGAATACGTACACCAATGCGGACAAGCTCCTAGAAGCAGCAGAGCAGTTGGC
TCAGACGGGGGAATGTGACCCCGAGGAGATCTACAAGGCAGCTCGACACCTGGAGGTGCGCATCCAAGACTTCGTGCGCAGGGTGGAGCAGCGGAAGCT
TCTCCTGGACATGTCTGTTTCCTTCCACACACACACCAAAGAGTTGTGGACATGGATGGAAGACCTTCAGAAGGAGATGTTGGAGGATGTCTGTGCAGATTC
TGTGGATGCAGTCCAGGAACTGATCAAGCAGTTCCAGCAGCAGCAGACCGCCACTCTAGATGCCACACTCAATGTCATCAAGGAAGGCGAAGACCTTATCC
AGCAGCTCAGGTCAGCGCCTCCCTCCCTCGGGGAGCCCAGCGAGGCCAGGGACTCGGCTGTGTCCAACAACAAAACACCCCACAGCAGCTCCATCAGCCAC
ATCGAGTCGGTCCTGCAGCAGCTTGATGATGCCCAGGTGCAGATGGAGGAGCTGTTCCACGAGCGGAAGATCAAGCTGGACATCTTCCTGCAACTGCGCAT
CTTTGAGCAGTACACCATCGAGGTGACAGCAGAGCTAGACGCCTGGAATGAAGACTTGCTTCGGCAGATGAATGACTTCAACACAGAGGACCTAACCCTGG
CAGAACAGCGGCTGCAGCGCCACACAGAACGGAAGCTAGCCATGAACAACATGACCTTTGAGGTTATCCAGCAGGGACAGGATCTGCACCAGTACATCAC
GGAGGTCCAGGCATCAGGAATTGAGTTGATCTGTGAAAAAGACATTGATCTGGCAGCCCAGGTGCAAGAGTTATTGGAATTTCTCCATGAGAAGCAGCATG
AATTGGAGCTCAATGCAGAGCAGACTCATAAGCGGCTAGAGCAGTGCCTCCAATTACGTCACCTCCAGGCTGAAGTCAAACAGGTTCTGGGATGGATCCGC
AATGGAGAGTCAATGCTCAACGCCAGCCTGGTCAATGCCAGCTCTTTGTCGGAAGCAGAGCAGCTGCAGCGGGAGCACGAGCAGTTCCAACTGGCCATCG
AGTCCCTCTTTCATGCCACTTCCTTGCAGAAGACGCACCAGAGTGCCCTGCAGGTACAGCAGAAAGCCGAGGTGCTGCTCCAGGCCGGCCACTACGATGCC
GATGCCATCCGGGAATGTGCTGAGAAGGTGGCCCTCCACTGGCAGCAGCTCATGCTGAAGATGGAAGACCGGCTAAAATTGGTCAATGCCTCTGTGGCCTT
TTACAAAACTTCTGAACAGGTGTGTAGTGTCCTGGAGAGCTTAGAGCAAGAATACCGGAGAGATGAGGACTGGTGTGGTGGACGAGATAAGCTGGGGCCA
GCAGCAGAGATCGACCATGTCATTCCCCTCATCAGCAAACATTTGGAACAAAAGGAGGCCTTTCTTAAGGCCTGCACCCTGGCTCGGCGGAATGCTGAGGT
GTTTCTCAAGTACATCCACAGGAACAACGTCAGCATGCCCAGTGTCGCCAGCCACACTCGGGGACCCGAGCAACAAGTGAAAGCCATCCTGAGTGAGCTCC
TGCAGAGGGAGAATCGCGTGCTGCATTTCTGGACCTTGAAGAAGCGGCGGTTAGACCAATGCCAGCAATATGTGGTGTTCGAGCGCAGCGCTAAGCAGGC
GCTTGACTGGATCCAAGAAACAGGTGAATTTTACCTCTCAACACATACCTCCACTGGAGAGACCACAGAGGAGACTCAGGAACTGCTGAAAGAATATGGGG
AATTCAGGGTGCCTGCCAAGCAAACAAAGGAGAAGGTGAAGCTTCTGATTCAGCTGGCCGATAGCTTTGTGGAAAAAGGCCACATTCATGCCACGGAGATA
AGGAAATGGGTGACCACGGTGGACAAGCACTACAGAGATTTCTCCCTGAGGATGGGAAAGTACCGATACTCACTGGAGAAAGCCCTAGGAGTCAACACAG
AGGATAATAAGGACCTGGAGCTGGATATTATCCCAGCAAGCCTTTCGGATCGGGAGGTCAAGCTGCGGGACGCCAACCACGAAGTCAATGAAGAGAAGCG
GAAGTCAGCCCGGAAGAAAGAATTTATTATGGCTGAACTACTCCAGACAGAGAAGGCTTATGTAAGGGATTTGCATGAGTGCTTAGAGACCTACCTGTGGG
AAATGACCAGTGGTGTGGAGGAGATCCCCCCTGGGATCCTCAATAAAGAGCATATCATCTTTGGCAACATCCAAGAGATCTACGATTTCCATAACAACATCT
TCCTCAAAGAGCTGGAGAAGTACGAGCAACTGCCTGAGGATGTGGGACACTGCTTTGTTACCTGGGCAGACAAATTTCAGATGTATGTCACCTACTGTAAAA
ACAAGCCTGATTCCAACCAGCTTATCCTGGAGCATGCGGGCACCTTCTTTGATGAGATACAACAGCGGCATGGTCTGGCCAACTCCATCTCTTCCTACCTAAT
TAAGCCTGTCCAAAGGATCACCAAATATCAACTGCTCCTGAAGGAACTTTTAACTTGCTGTGAAGAAGGGAAAGGGGAGCTCAAGGATGGCCTGGAGGTG
ATGCTCAGTGTCCCAAAGAAAGCCAATGATGCCATGCATGTCAGCATGCTGGAAGGGTTCGACGAGAACCTGGATGTGCAGGGGGAGTTGATTCTCCAGG
ATGCCTTTCAAGTGTGGGACCCGAAGTCGCTGATCCGGAAGGGGCGGGAGCGGCACTTGTTCCTCTTTGAGATCTCCTTGGTTTTTAGCAAGGAGATCAAA
GATTCTTCAGGACACACGAAATATGTTTACAAGAACAAGCTACTGACCTCAGAGCTGGGTGTGACCGAGCACGTGGAGGGCGATCCCTGCAAATTCGCCTT
GTGGTCTGGGCGCACCCCATCCTCAGACAATAAAACAGTGCTGAAAGCCTCCAACATTGAAACCAAGCAGGAGTGGATCAAGAACATTCGAGAAGTGATTC
AAGAAAGGATCATTCACCTGAAAGGAGCTTTAAAGGAGCCACTTCAGCTCCCCAAAACACCAGCCAAACAGAGGAACAATAGTAAGAGGGATGGAGTGGA
GGATATTGACAGCCAGGGGGATGGGAGCAGCCAACCAGACACCATCTCCATTGCTTCTAGGACCTCTCAGAACACAGTGGACAGTGACAAGCTCTCTGGTG
GATGTGAGCTGACAGTGGTCCTCCAGGACTTCAGTGCGGGCCACAGCAGTGAGCTGACCATCCAGGTGGGGCAGACGGTAGAGCTGCTGGAGCGGCCCA
GCGAGCGGCCTGGTTGGTGTCTGGTCCGTACCACCGAACGGAGCCCGCCCTTGGAGGGTCTGGTCCCCAGCAGCGCCCTGTGCATCTCACACTCCCGAAGC
AGCGTGGAGATGGACTGCTTCTTCCCCTTGGTGAAAGATGCATACTCTCATTCCTCAAGCGAGAATGGAGGCAAGTCCGAGTCCGTGGCCAACCTGCAGGC
CCAGCCCTCCCTGAACTCCATCCACAGTTCCCCGGGTCCCAAGCGCTCCACCAACACTCTTAAGAAGTGGCTGACGAGTCCTGTGCGTCGGCTTAACAGCGG
GAAGGCAGATGGAAACATCAAAAAGCAGAAGAAAGTTCGCGATGGTCGGAAGAGCTTTGACCTGGGATCTCCCAAGCCTGGGGATGAAACAACCCCTCAG
GGAGACAGCGCTGATGAGAAGAGCAAGAAAGGTTGGGGTGAAGATGAGCCGGATGAAGAGTCACACACACCCCTCCCACCACCTATGAAGATTTTTGACA
ACGACCCTACACAGGATGAAATGAGTCTAGAAGGAAGCTCATACCGGGGGAGCTTGAAAGACCCTGCAGGCTGCCTGAATGAGGGGATGGCCCCACCCAC
ACCTCCTAAAAACCCAGAAGAAGAACAGAAAGCCAAGGCCCTGAGAGGCAGGATGTTTGTCCTGAATGAGCTGGTACAGACAGAGAAAGACTATGTCAAG
GATCTGGGCATTGTGGTGGAGGGCTTCATGAAGAGAATAGAAGAAAAGGGTGTCCCTGAGGATATGCGAGGAAAGGACAAAATCGTGTTTGGAAATATTC
ATCAGATTTATGACTGGCATAAGGATTTTTTCCTGGCGGAACTGGAAAAGTGTATCCAGGAGCAAGACAGATTGGCACAGCTCTTTATTAAGCACGAGCGG
AAGCTGCACATCTACGTGTGGTATTGTCAGAATAAGCCGCGCTCAGAGTACATCGTTGCTGAGTATGACGCCTACTTTGAGGAGGTAAAACAGGAGATAAA
TCAGAGGCTGACACTGAGTGACTTCCTCATCAAGCCCATTCAGAGAATAACAAAATACCAGTTGCTCCTCAAGGACTTCCTGAGATACAGTGAGAAGGCTGG
TTTGGAGTGTTCAGATATTGAGAAAGCAGTGGAGTTAATGTGCCTTGTTCCCAAACGCTGCAATGACATGATGAATCTAGGACGTCTGCAGGGCTTTGAGG
GCACTCTGACTGCTCAGGGGAAGCTGCTGCAGCAGGACACATTCTATGTGATCGAGCTGGATGCAGGCATGCAGTCCCGGACCAAAGAGAGGCGCGTGTT
CCTCTTCGAGCAGATTGTCATCTTCAGTGAACTGCTCAGGAAGGGATCCCTCACCCCTGGCTACATGTTCAAAAGGAGCATCAAGATGAATTACTTGGTCCTG
GAGGAGAATGTGGACAATGATCCCTGCAAGTTTGCACTCATGAACAGAGAGACTTCTGAGAGGGTTGTTCTGCAAGCCGCCAACGCTGACATCCAGCAGGC
CTGGGTGCAGGACATCAATCAAGTCTTAGAAACACAGCGAGACTTTTTGAATGCACTGCAATCGCCCATTGAGTATCAACGGAAAGAAAGGAGCACAGCTG
TGATGAGGTCTCAACCTGCCAGGCTTCCCCAAGCCAGCCCCAGGCCCTACTCCTCTGTTCCTGCGGGCTCAGAGAAGCCCCCAAAGGGCTCCAGCTATAACC
CACCTCTGCCTCCCCTGAAGATATCTACCTCCAATGGCAGTCCAGGGTTTGAATACCACCAGCCTGGGGACAAGTTCGAAGCCAGCAAGAACGACCTGGGA
GGCTGCAATGGGACCTCGTCCATGGCCGTGATCAAAGATTACTATGCACTGAAGGAGAATGAAATCTGTGTGAGCCAAGGTGAGGTGGTCCAGGTCCTCGC
CGTCAACCAGCAGAACATGTGTCTGGTGTACCAGCCTGCCAGCGACCATTCCCCCGCCGCCGAGGGCTGGGTCCCAGGCAGCATCCTGGCGCCCCTCACCA
AAGCCACAGCAGCAGAAAGTAGTGACGGGAGCATCAAGTAA

>isoform 268 ΔEx37

ATGAACCCCCCTGAGGGAGCAGCGGAGGAAGGAGGAGCAGCAGACTCGGACGTGGACGCCTTTTTCCGGACAGGGTCTTTTCGGAATGATGGTTTGAAA
GCTTCTGATGTCCTTCCTATCCTAAAGGAAAAGGTGGCCTTCGTGTCTGGGGGTCGTGATAAGCGAGGCGGACCCATCCTGACCTTCCCTGCTCGCAGCAA
TCATGACAGAATAAGACAGGAAGACCTGCGGAAACTCGTGACGTATTTGGCCAGCGTGCCAAGTGAGGACGTGTGCAAACGTGGCTTCACTGTCATCATC
GACATGCGGGGCTCCAAGTGGGACCTCATCAAGCCCCTCCTCAAAACGCTGCAGGAAGCCTTTCCAGCTGAGATCCATGTGGCCCTCATCATTAAACCCGA
CAACTTCTGGCAGAAACAGAAGACCAACTTTGGCAGCTCCAAATTCATCTTTGAGACGAGCATGGTATCTGTGGAGGGCCTCACAAAGCTGGTGGACCCCT
CCCAGCTGACGGAGGAGTTTGATGGCTCCCTGGACTACAACCATGAGGAGTGGATCGAACTGCGGCTCTCCCTGGAGGAGTTCTTCAACAGCGCCGTGCA
CCTGCTCTCGCGCCTCGAGGACCTCCAGGAGATGCTAGCCCGGAAGGAGTTTCCTGTGGATGTGGAGGGCTCTCGGCGGCTCATTGACGAACACACACAG
CTCAAGAAAAAGGTGCTGAAGGCCCCTGTGGAGGAGCTGGACCGGGAGGGGCAGCGGCTGCTGCAGTGCATCCGCTGCAGCGACGGCTTCTCAGGACG
CAACTGCATCCCGGGCAGTGCTGACTTCCAGAGCCTGGTGCCCAAGATCACCAGTCTCCTGGACAAGCTGCACTCCACCCGGCAGCACCTGCACCAGATGT
GGCATGTGCGCAAGCTCAAGCTGGACCAGTGCTTTCAGCTGCGGCTCTTCGAGCAGGATGCTGAGAAGATGTTTGACTGGATAAGCCACAACAAGGAGTT
ATTCCTCCAGAGCCACACGGAGATCGGAGTCAGCTACCAGTACGCCCTTGACCTCCAGACGCAGCACAATCACTTTGCCATGAACTCCATGAATGCCTATGT
CAACATCAACCGCATCATGTCCGTGGCTTCCCGCCTCTCTGAGGCCGGTCATTATGCCTCACAACAAATCAAGCAGATCTCCACCCAGCTGGACCAGGAGT
GGAAGAGCTTCGCTGCTGCCCTGGATGAACGCAGCACCATCCTCGCCATGTCTGCTGTGTTCCACCAGAAGGCTGAGCAGTTCCTGTCGGGAGTGGATGC
CTGGTGCAAGATGTGCAGTGAAGGTGGTCTGCCATCCGAGATGCAAGACCTAGAGCTGGCAATCCACCACCACCAGACCTTGTATGAGCAGGTGACCCAA
GCCTACACAGAGGTCAGCCAGGATGGCAAAGCACTACTTGATGTGCTGCAGCGGCCCCTGAGCCCTGGGAACTCCGAATCCCTCACGGCCACAGCCAACT
ACTCCAAGGCAGTGCACCAGGTGCTGGACGTGGTGCATGAGGTGTTACATCACCAGCGACGGCTGGAGAGCATCTGGCAGCACCGCAAGGTGCGGCTCC
ACCAGCGGCTGCAGCTCTGCGTCTTCCAGCAGGATGTACAGCAGGTGTTGGACTGGATTGAAAACCATGGTGAGGCCTTTCTCAGCAAACACACTGGAGT
TGGGAAGTCCCTACATCGAGCCCGGGCCCTGCAGAAGAGGCATGATGACTTTGAAGAGGTGGCTCAGAATACGTACACCAATGCGGACAAGCTCCTAGA
AGCAGCAGAGCAGTTGGCTCAGACGGGGGAATGTGACCCCGAGGAGATCTACAAGGCAGCTCGACACCTGGAGGTGCGCATCCAAGACTTCGTGCGCAG
GGTGGAGCAGCGGAAGCTTCTCCTGGACATGTCTGTTTCCTTCCACACACACACCAAAGAGTTGTGGACATGGATGGAAGACCTTCAGAAGGAGATGTTG
GAGGATGTCTGTGCAGATTCTGTGGATGCAGTCCAGGAACTGATCAAGCAGTTCCAGCAGCAGCAGACCGCCACTCTAGATGCCACACTCAATGTCATCAA
GGAAGGCGAAGACCTTATCCAGCAGCTCAGGTCAGCGCCTCCCTCCCTCGGGGAGCCCAGCGAGGCCAGGGACTCGGCTGTGTCCAACAACAAAACACCC
CACAGCAGCTCCATCAGCCACATCGAGTCGGTCCTGCAGCAGCTTGATGATGCCCAGGTGCAGATGGAGGAGCTGTTCCACGAGCGGAAGATCAAGCTG
GACATCTTCCTGCAACTGCGCATCTTTGAGCAGTACACCATCGAGGTGACAGCAGAGCTAGACGCCTGGAATGAAGACTTGCTTCGGCAGATGAATGACTT
CAACACAGAGGACCTAACCCTGGCAGAACAGCGGCTGCAGCGCCACACAGAACGGAAGCTAGCCATGAACAACATGACCTTTGAGGTTATCCAGCAGGG
ACAGGATCTGCACCAGTACATCACGGAGGTCCAGGCATCAGGAATTGAGTTGATCTGTGAAAAAGACATTGATCTGGCAGCCCAGGTGCAAGAGTTATTG
GAATTTCTCCATGAGAAGCAGCATGAATTGGAGCTCAATGCAGAGCAGACTCATAAGCGGCTAGAGCAGTGCCTCCAATTACGTCACCTCCAGGCTGAAG
TCAAACAGGTTCTGGGATGGATCCGCAATGGAGAGTCAATGCTCAACGCCAGCCTGGTCAATGCCAGCTCTTTGTCGGAAGCAGAGCAGCTGCAGCGGGA
GCACGAGCAGTTCCAACTGGCCATCGAGTCCCTCTTTCATGCCACTTCCTTGCAGAAGACGCACCAGAGTGCCCTGCAGGTACAGCAGAAAGCCGAGGTG
CTGCTCCAGGCCGGCCACTACGATGCCGATGCCATCCGGGAATGTGCTGAGAAGGTGGCCCTCCACTGGCAGCAGCTCATGCTGAAGATGGAAGACCGG
CTAAAATTGGTCAATGCCTCTGTGGCCTTTTACAAAACTTCTGAACAGGTGTGTAGTGTCCTGGAGAGCTTAGAGCAAGAATACCGGAGAGATGAGGACT
GGTGTGGTGGACGAGATAAGCTGGGGCCAGCAGCAGAGATCGACCATGTCATTCCCCTCATCAGCAAACATTTGGAACAAAAGGAGGCCTTTCTTAAGGC
CTGCACCCTGGCTCGGCGGAATGCTGAGGTGTTTCTCAAGTACATCCACAGGAACAACGTCAGCATGCCCAGTGTCGCCAGCCACACTCGGGGACCCGAG
CAACAAGTGAAAGCCATCCTGAGTGAGCTCCTGCAGAGGGAGAATCGCGTGCTGCATTTCTGGACCTTGAAGAAGCGGCGGTTAGACCAATGCCAGCAAT
ATGTGGTGTTCGAGCGCAGCGCTAAGCAGGCGCTTGACTGGATCCAAGAAACAGGTGAATTTTACCTCTCAACACATACCTCCACTGGAGAGACCACAGA
GGAGACTCAGGAACTGCTGAAAGAATATGGGGAATTCAGGGTGCCTGCCAAGCAAACAAAGGAGAAGGTGAAGCTTCTGATTCAGCTGGCCGATAGCTT
TGTGGAAAAAGGCCACATTCATGCCACGGAGATAAGGAAATGGGTGACCACGGTGGACAAGCACTACAGAGATTTCTCCCTGAGGATGGGAAAGTACCG
ATACTCACTGGAGAAAGCCCTAGGAGTCAACACAGAGGATAATAAGGACCTGGAGCTGGATATTATCCCAGCAAGCCTTTCGGATCGGGAGGTCAAGCTG
CGGGACGCCAACCACGAAGTCAATGAAGAGAAGCGGAAGTCAGCCCGGAAGAAAGAATTTATTATGGCTGAACTACTCCAGACAGAGAAGGCTTATGTA
AGGGATTTGCATGAGTGCTTAGAGACCTACCTGTGGGAAATGACCAGTGGTGTGGAGGAGATCCCCCCTGGGATCCTCAATAAAGAGCATATCATCTTTG
GCAACATCCAAGAGATCTACGATTTCCATAACAACATCTTCCTCAAAGAGCTGGAGAAGTACGAGCAACTGCCTGAGGATGTGGGACACTGCTTTGTTACC
TGGGCAGACAAATTTCAGATGTATGTCACCTACTGTAAAAACAAGCCTGATTCCAACCAGCTTATCCTGGAGCATGCGGGCACCTTCTTTGATGAGATACA
ACAGCGGCATGGTCTGGCCAACTCCATCTCTTCCTACCTAATTAAGCCTGTCCAAAGGATCACCAAATATCAACTGCTCCTGAAGGAACTTTTAACTTGCTGT
GAAGAAGGGAAAGGGGAGCTCAAGGATGGCCTGGAGGTGATGCTCAGTGTCCCAAAGAAAGCCAATGATGCCATGCATGTCAGCATGCTGGAAGGGTT
CGACGAGAACCTGGATGTGCAGGGGGAGTTGATTCTCCAGGATGCCTTTCAAGTGTGGGACCCGAAGTCGCTGATCCGGAAGGGGCGGGAGCGGCACTT
GTTCCTCTTTGAGATCTCCTTGGTTTTTAGCAAGGAGATCAAAGATTCTTCAGGACACACGAAATATGTTTACAAGAACAAGCTACTGACCTCAGAGCTGGG
TGTGACCGAGCACGTGGAGGGCGATCCCTGCAAATTCGCCTTGTGGTCTGGGCGCACCCCATCCTCAGACAATAAAACAGTGCTGAAAGCCTCCAACATTG
AAACCAAGCAGGAGTGGATCAAGAACATTCGAGAAGTGATTCAAGAAAGGATCATTCACCTGAAAGGAGCTTTAAAGGAGCCACTTCAGCTCCCCAAAAC
ACCAGCCAAACAGAGGAACAATAGTAAGAGGGATGGAGTGGAGGATATTGACAGCCAGGGGGATGGGAGCAGCCAACCAGACACCATCTCCATTGCTTC
TAGGACCTCTCAGAACACAGTGGACAGTGACAAGCTCTCTGGTGGATGTGAGCTGACAGTGGTCCTCCAGGACTTCAGTGCGGGCCACAGCAGTGAGCTG
ACCATCCAGGTGGGGCAGACGGTAGAGCTGCTGGAGCGGCCCAGCGAGCGGCCTGGTTGGTGTCTGGTCCGTACCACCGAACGGAGCCCGCCCTTGGAG
GGTCTGGTCCCCAGCAGCGCCCTGTGCATCTCACACTCCCGAAGCAGCGTGGAGATGGACTGCTTCTTCCCCTTGGTGAAAGATGCATACTCTCATTCCTCA
AGCGAGAATGGAGGCAAGTCCGAGTCCGTGGCCAACCTGCAGGCCCAGCCCTCCCTGAACTCCATCCACAGTTCCCCGGGTCCCAAGCGCTCCACCAACA
CTCTTAAGAAGTGGCTGACGAGTCCTGTGCGTCGGCTTAACAGCGGGAAGGCAGATGGAAACATCAAAAAGCAGAAGAAAGTTCGCGATGGTCGGAAGA
GCTTTGACCTGGGATCTCCCAAGCCTGGGGATGAAACAACCCCTCAGGGAGACAGCGCTGATGAGAAGAGCAAGAAAGGTTGGGGTGAAGATGAGCCG
GATGAAGAGTCACACACACCCCTCCCACCACCTATGAAGATTTTTGACAACGACCCTACACAGGATGAAATGAGTCTAGAAGGAAGCTCATACCGGGGGA
GCTTGAAAGACCCTGCAGGCTGCCTGAATGAGGGGATGGCCCCACCCACACCTCCTAAAAACCCAGAAGAAGAACAGAAAGCCAAGGCCCTGAGAGGCA
GGATGTTTGTCCTGAATGAGCTGGTACAGACAGAGAAAGACTATGTCAAGGATCTGGGCATTGTGGTGGAGGGCTTCATGAAGAGAATAGAAGAAAAGG
GTGTCCCTGAGGATATGCGAGGAAAGGACAAAATCGTGTTTGGAAATATTCATCAGATTTATGACTGGCATAAGGATTTTTTCCTGGCGGAACTGGAAAA
GTGTATCCAGGAGCAAGACAGATTGGCACAGCTCTTTATTAAGCACGAGCGGAAGCTGCACATCTACGTGTGGTATTGTCAGAATAAGCCGCGCTCAGAG
TACATCGTTGCTGAGTATGACGCCTACTTTGAGGAGGTAAAACAGGAGATAAATCAGAGGCTGACACTGAGTGACTTCCTCATCAAGCCCATTCAGAGAAT
AACAAAATACCAGTTGCTCCTCAAGGACTTCCTGAGATACAGTGAGAAGGCTGGTTTGGAGTGTTCAGATATTGAGAAAGCAGTGGAGTTAATGTGCCTT
GTTCCCAAACGCTGCAATGACATGATGAATCTAGGACGTCTGCAGGGCTTTGAGGGCACTCTGACTGCTCAGGGGAAGCTGCTGCAGCAGGACACATTCT
ATGTGATCGAGCTGGATGCAGGCATGCAGTCCCGGACCAAAGAGAGGCGCGTGTTCCTCTTCGAGCAGATTGTCATCTTCAGTGAACTGCTCAGGAAGGG
ATCCCTCACCCCTGGCTACATGTTCAAAAGGAGCATCAAGATGAATTACTTGGTCCTGGAGGAGAATGTGGACAATGATCCCTGCAAGTTTGCACTCATGA
ACAGAGAGACTTCTGAGAGGGTTGTTCTGCAAGCCGCCAACGCTGACATCCAGCAGGCCTGGGTGCAGGACATCAATCAAGTCTTAGAAACACAGCGAG
ACTTTTTGAATGCACTGCAATCGCCCATTGAGTATCAACGGAAAGAAAGGAGCACAGCTGTGATGAGGTCTCAACCTGCCAGGCTTCCCCAAGCCAGCCCC
AGGCCCTACTCCTCTGTTCCTGCGGGCTCAGAGAAGCCCCCAAAGGGCTCCAGCTATAACCCACCTCTGCCTCCCCTGAAGATATCTACCTCCAATGGCAGT
CCAGGGTTTGAATACCACCAGCCTGGGGACAAGTTCGAAGCCAGCAAGAACGACCTGGGAGGCTGCAATGGGACCTCGTCCATGGCCGTGATCAAAGAT
TACTATGCACTGAAGGAGAATGAAATCTGTGTGAGCCAAGGTGAGGTGGTCCAGGTCCTCGCCGTCAACCAGCAGAACATGTGTCTGGTGTACCAGCCTG
CCAGCGACCATTCCCCCGCCGCCGAGGGCTGGGTCCCAGGCAGCATCCTGGCGCCCCTCACCAAAGCCACAGCAGCAGAAAGTAGTGACGGGAGCATCA
AGAAGTCATGTTCATGGCATACTCTACGCATGAGAAAGCGGGCGGAAGTGGAGAACACGGGTAAAAATGAAGCCACAGGGCCTCGTAAACCCAAGGATA
TTCTGGGCAACAAAGTCTCTGTTAAAGAGACGAACAGTTCCGAGGAATCAGAGTGTGATGATCTTGACCCTAATACTAGCATGGAGATCTTAAATCCAAAT
TTCATCCAAGAAGTGGCCCCAGAATTCCTTGTGCCCTTGGTGGATGTGACCTGCTTGCTTGGGGACACAGTGATACTGCAGTGCAAAGTCTGTGGGCGGCC
AAAGCCCACCATCACTTGGAAGGGTCCAGACCAGAACATCCTTGACACTGATAACAGCTCAGCCACATACACGGTCTCCTCTTGTGATTCTGGAGAAATCA
CCCTGAAGATCTGTAATCTGATGCCCCAAGACAGTGGGATTTATACCTGCATAGCAACAAATGACCACGGGACCACATCAACGTCTGCAACAGTCAAAGTG
CAAGGTGTTCCAGCAGCCCCTAACCGCCCCATTGCCCAGGAGAGAAGCTGCACCTCCGTGATTCTCCGCTGGCTGCCCCCCTCCAGCACAGGAAACTGCAC
TATTTCTGGTTACACTGTGGAGTACAGAGAGGAAGGTTCTCAGATCTGGCAGCAGTCAGTGGCTTCGACCTTGGACACTTACCTCGTCATCGAAGACCTTA
GTCCCGGGTGTCCTTATCAGTTCAGAGTCAGTGCCAGTAACCCCTGGGGAATCAGCCTTCCCAGCGAGCCCTCGGAGTTTGTGCGACTTCCAGAATATGAT
GCTGCTGCTGATGGTGCCACCATTTCTTGGAAGGAAAATTTTGACTCAGCTTACACTGAGCTGAATGAAATTGGAAGAGGCCGTTTCTCTATAGTAAAGAA
ATGCATTCACAAAGCTACCCGCAAAGATGTGGCTGTGAAATTTGTTAGCAAAAAAATGAAGAAGAAAGAACAGGCTGCCCACGAGGCTGCCCTGCTTCAG

CACCTACAGCACCCCCAGTACATCACTCTCCATGACACCTATGAGTCCCCCACATCCTACATCCTGATCTTGGAACTGATGGATGATGGCCGGCTCTTAGACT
ACCTTATGAATCATGATGAACTGATGGAGGAAAAAGTAGCTTTCTATATCCGAGACATCATGGAGGCTCTGCAGTACCTTCACAACTGCAGGGTTGCACAT
TTGGACATAAAGCCTGAAAACCTGCTCATTGACCTACGGATTCCAGTGCCTCGAGTGAAGCTCATTGACTTGGAGGATGCTGTCCAGATCTCGGGTCACTTC
CACATTCACCACCTGCTGGGGAACCCTGAGTTTGCTGCCCCAGAAGTCATTCAAGGCATCCCCGTCTCCCTGGGGACAGACATCTGGAGCATCGGGGTTCT
GACATATGTCATGCTGAGTGGGGTCTCCCCCTTCTTGGATGAGAGCAAAGAGGAGACATGTATCAACGTATGCAGGGTGGATTTCAGCTTCCCCCATGAAT
ACTTCTGTGGTGTGAGCAATGCTGCCAGAGATTTCATCAATGTGATCTTACAGGAAGATTTTCGGAGGCGGCCCACAGCAGCCACATGCTTGCAGCATCCA
TGGCTGCAGCCCCATAATGGCAGCTACTCTAAGATCCCCCTGGACACCTCCCGCCTAGCATGCTTCATAGAACGTCGCAAGCACCAGAATGATGTGCGGCC
TATTCCCAATGTCAAGAGCTACATTGTCAACCGGGTGAACCAAGGGACGTAG

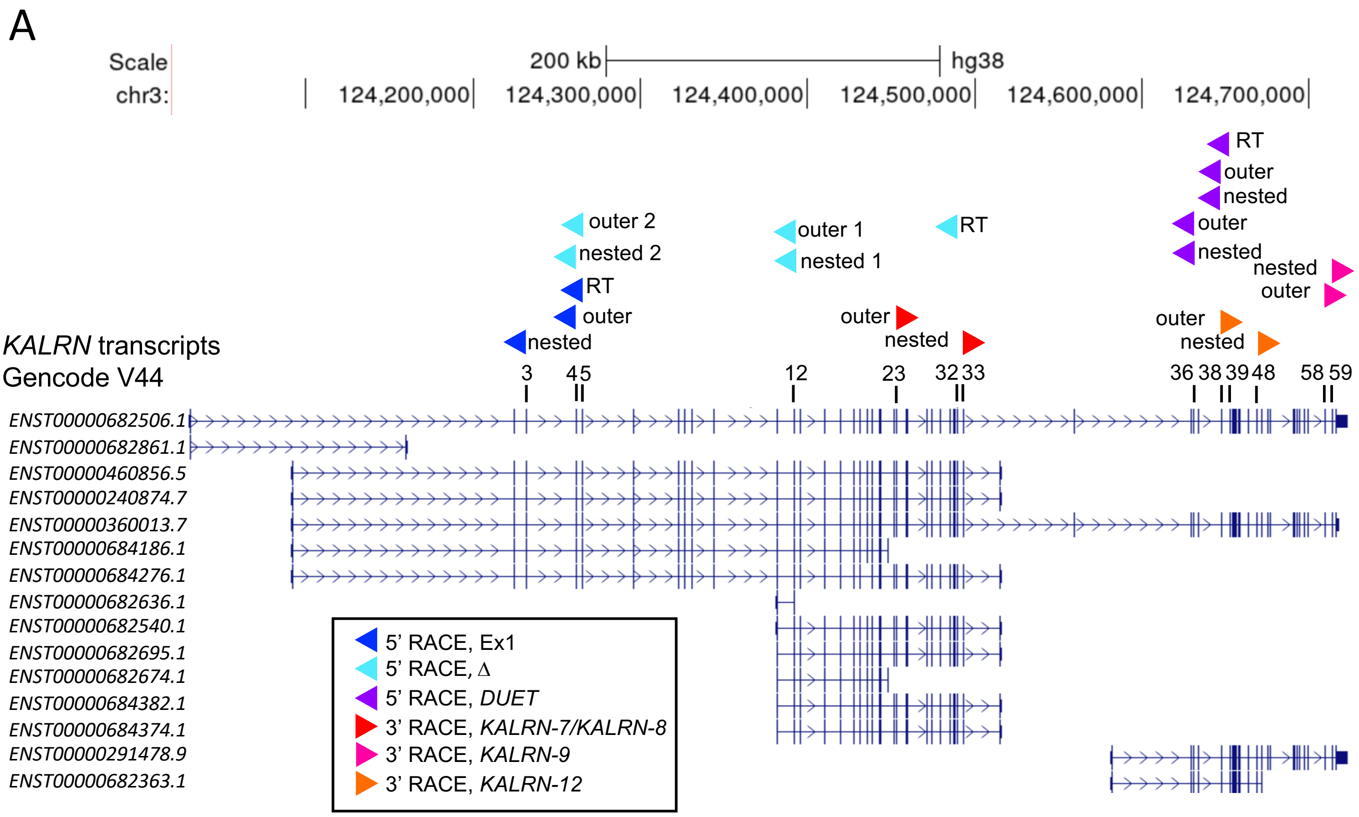

Figure S3. Genomic location of 5' and 3' RACE primers.

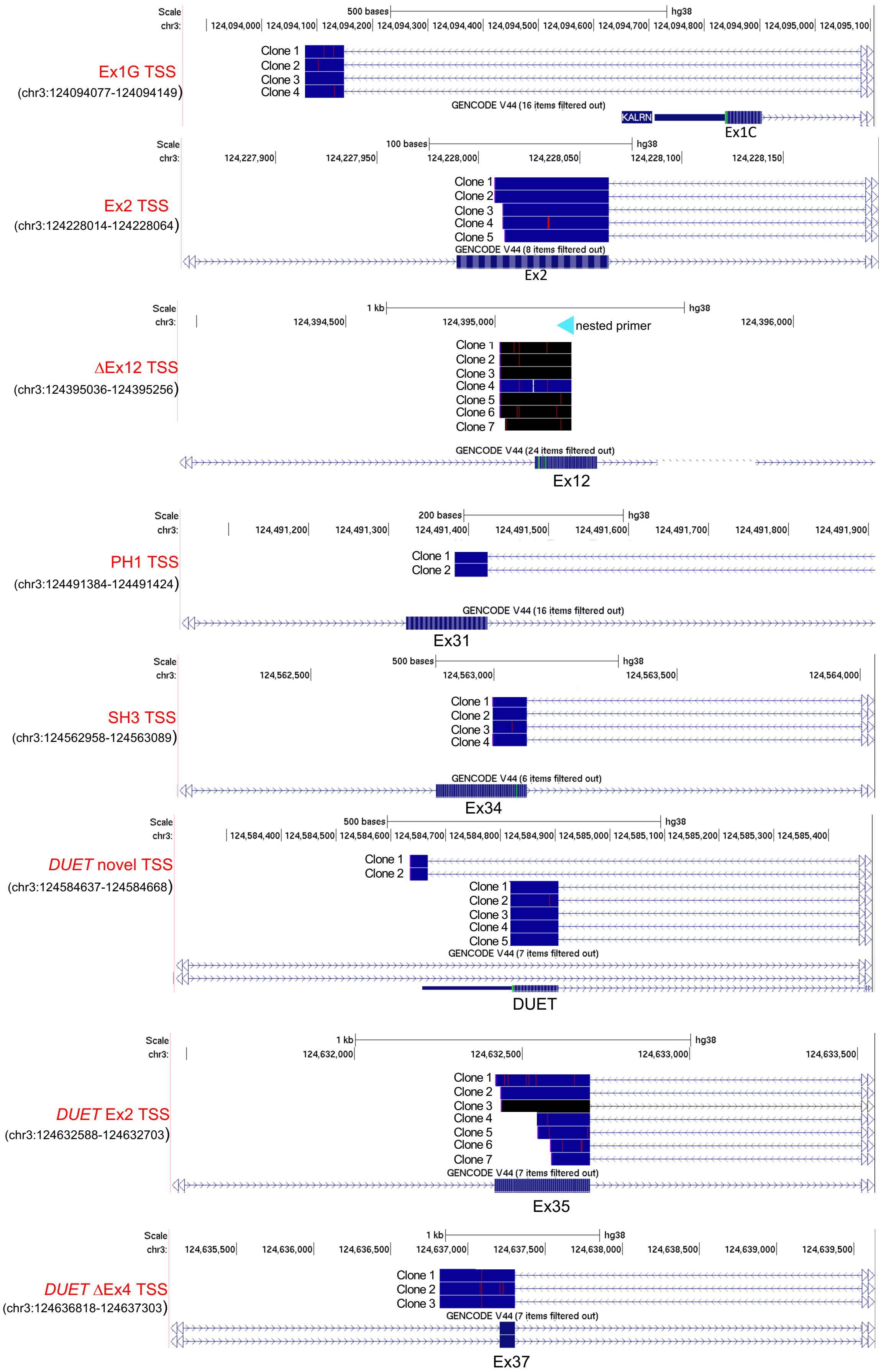

Figure S4. UCSC BLAT of novel alternative *KALRN* TSS 5' RACE clone ends.Overlapping genomic coordinates of clones are indicated in brackets.

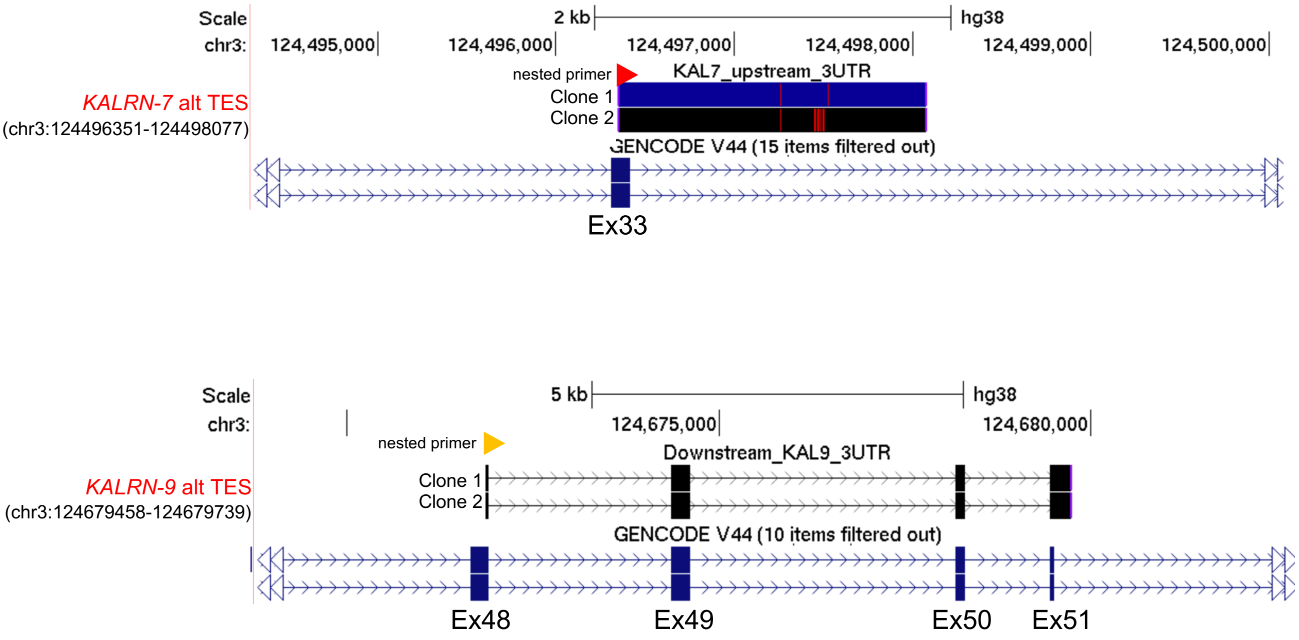

Figure S5. UCSC BLAT of novel alternative *KALRN* TES 3' RACE clone ends. Overlapping genomic coordinates of clones are indicated in brackets.

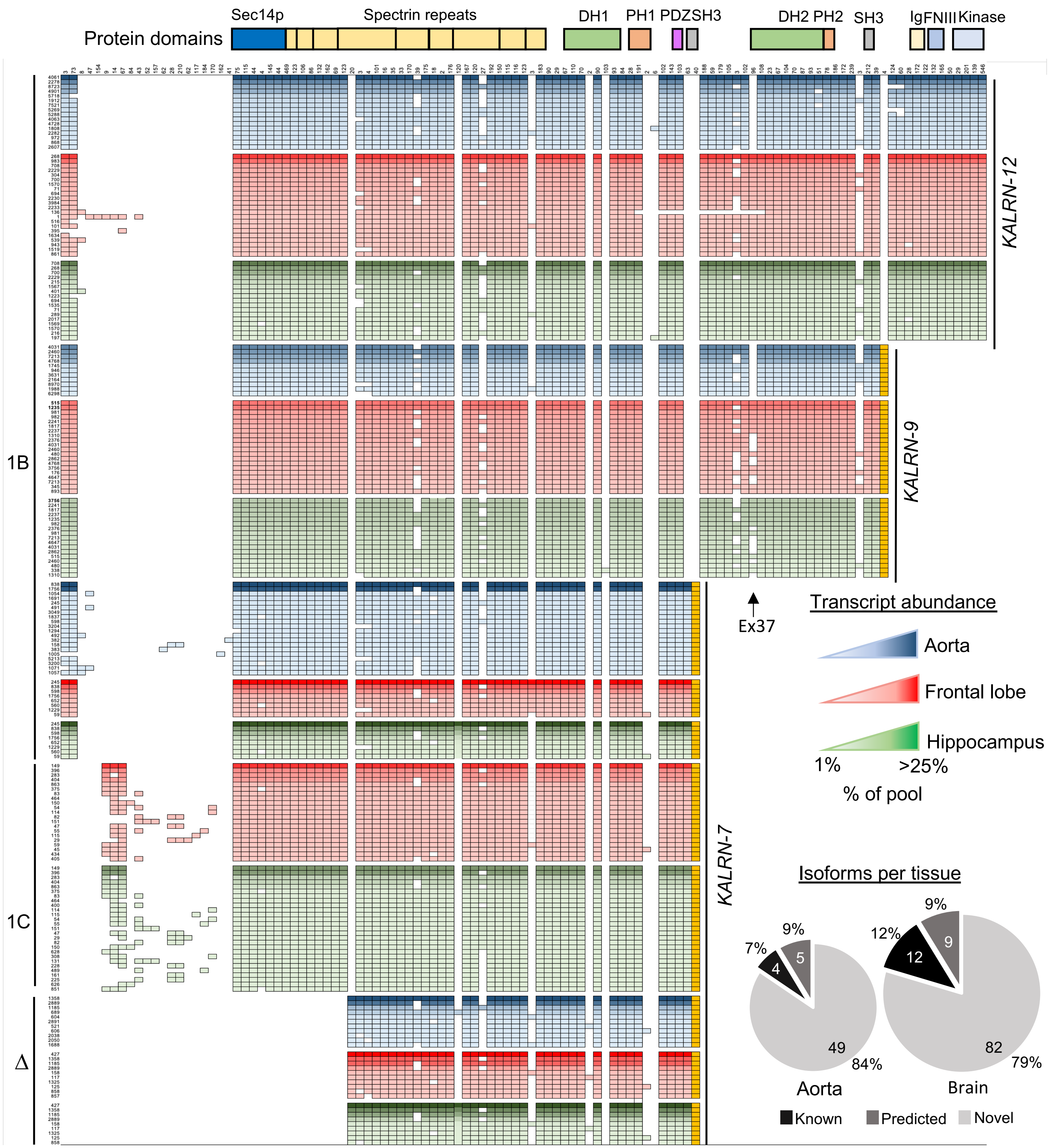

Figure S6. Alignment of nanopore long-read sequences for *KALRN* transcripts contributing to >1% of the transcript pool for abundant TSS/TES combinations for aorta, frontal lobe and hippocampus. Small boxes represent exonic parts (alternative splice donor/acceptor sites, not to scale) identified in analysis of all transcripts. The nucleotide (nt) length of key exonic parts are indicated at the top. Unique I.D.s for each isoform are indicated on the left. The regions coding the different protein domains (individually coloured) are indicated at the top. Transcript isoform percentage contribution to individual amplicon pools are indicated by colour gradient. Blue = aorta, red=frontal lobe and green = hippocampus. Pie charts show the total number and proportion of known (GENCODE and/or NCBI RefSeq), predicted (NCBI RefSeq predicted transcripts) and novel isoforms in brain and aorta.

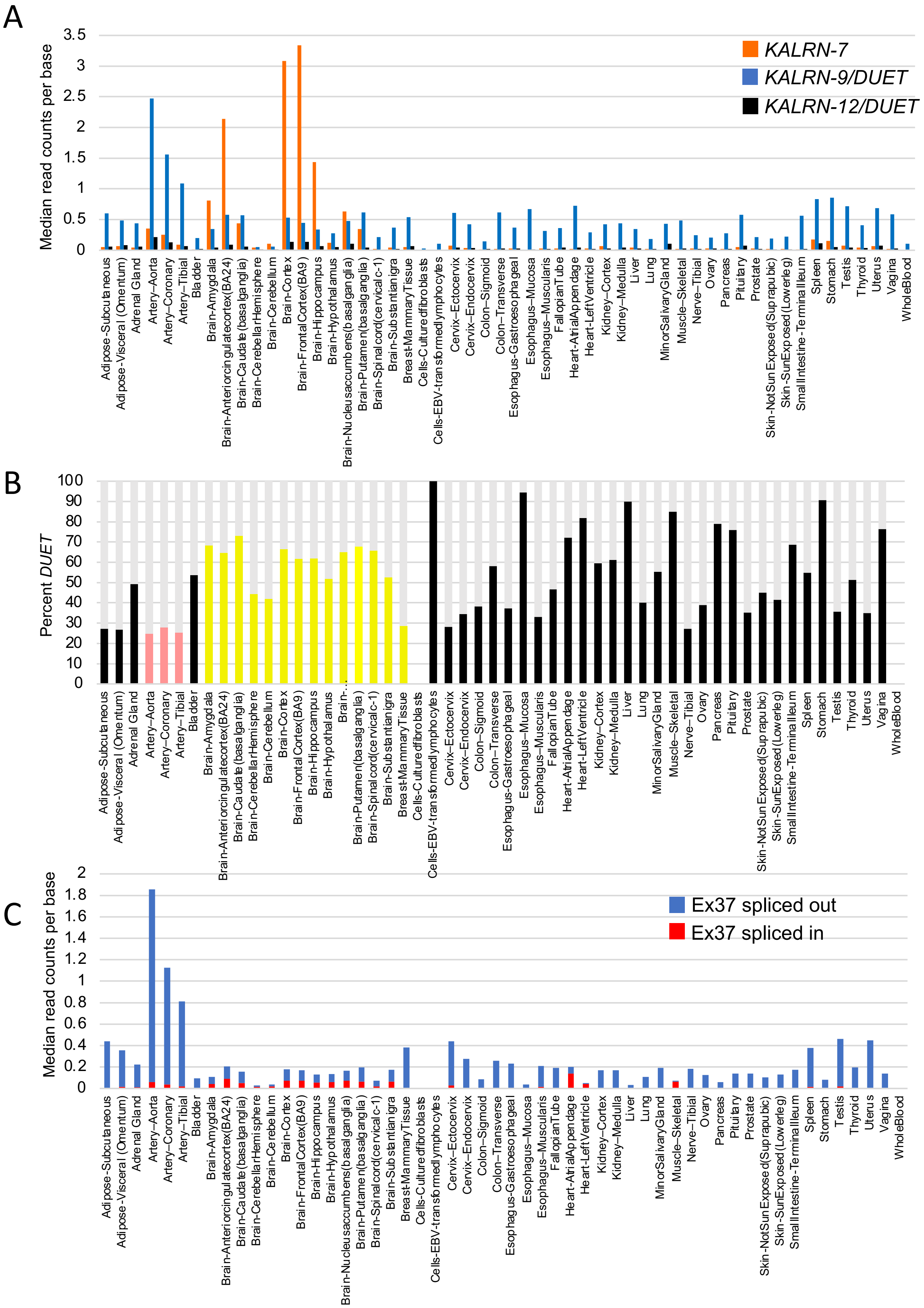

Figure S7. Relative levels of major *KALRN* transcripts in different tissues in GTEx short read RNA-seq data (A) Major *KALRN* transcript terminal exon read counts based on short read RNA-seq data. (B) Estimated percent *DUET* transcript contribution to *KALRN-9 and KALRN-12* terminal exon read counts. (C) Estimated level of *KALRN-9* transcripts with and without exon 37. The data used for the analyses above were obtained from: The GTEx Portal (GTEx Analysis Release V8, dbGaP Accession phs000424.v8.p2) on 21/12/23.

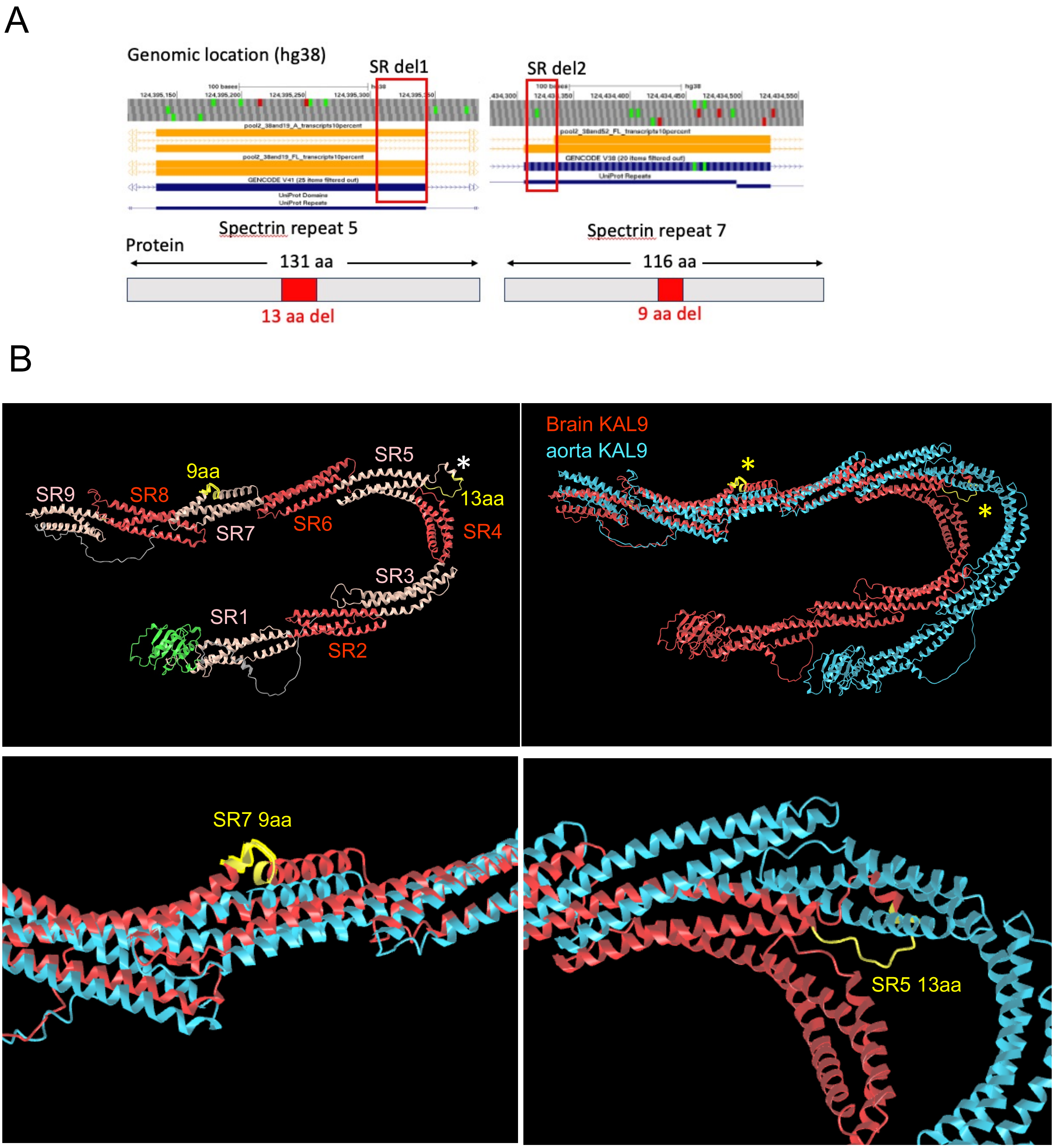

Figure S8. Spectrin repeat deletions due to alternative splicing and their potential effect on 3D protein structure. (A) UCSC browser (hg38) screen shot of mapped long read sequences for top expressed frontal lobe and aorta *KALRN-9* isoforms showing the genomic location of alternative splicing sites leading to 13 and 9 amino acid deletions in the spectrin repeat 5 and 7 protein regions, respectively. (B) AlphaFold predicted 3D protein structures for the N-terminal region encoded by the most abundant frontal cortex and aorta *KALRN-9* transcripts. On the left, the sec14p/CRAL-TRIO domain (green), the individual spectrin repeats (alternating pink and red) and the deletions regions due to alternative splicing (yellow) are indicated. *Loop regions predicted to be involved in protein-protein interactions (Vishwanatha et al., 2012). On the right, overlay of aorta (blue) and brain (red) predicted protein structures, and below, zoomed in views of each deletion region.

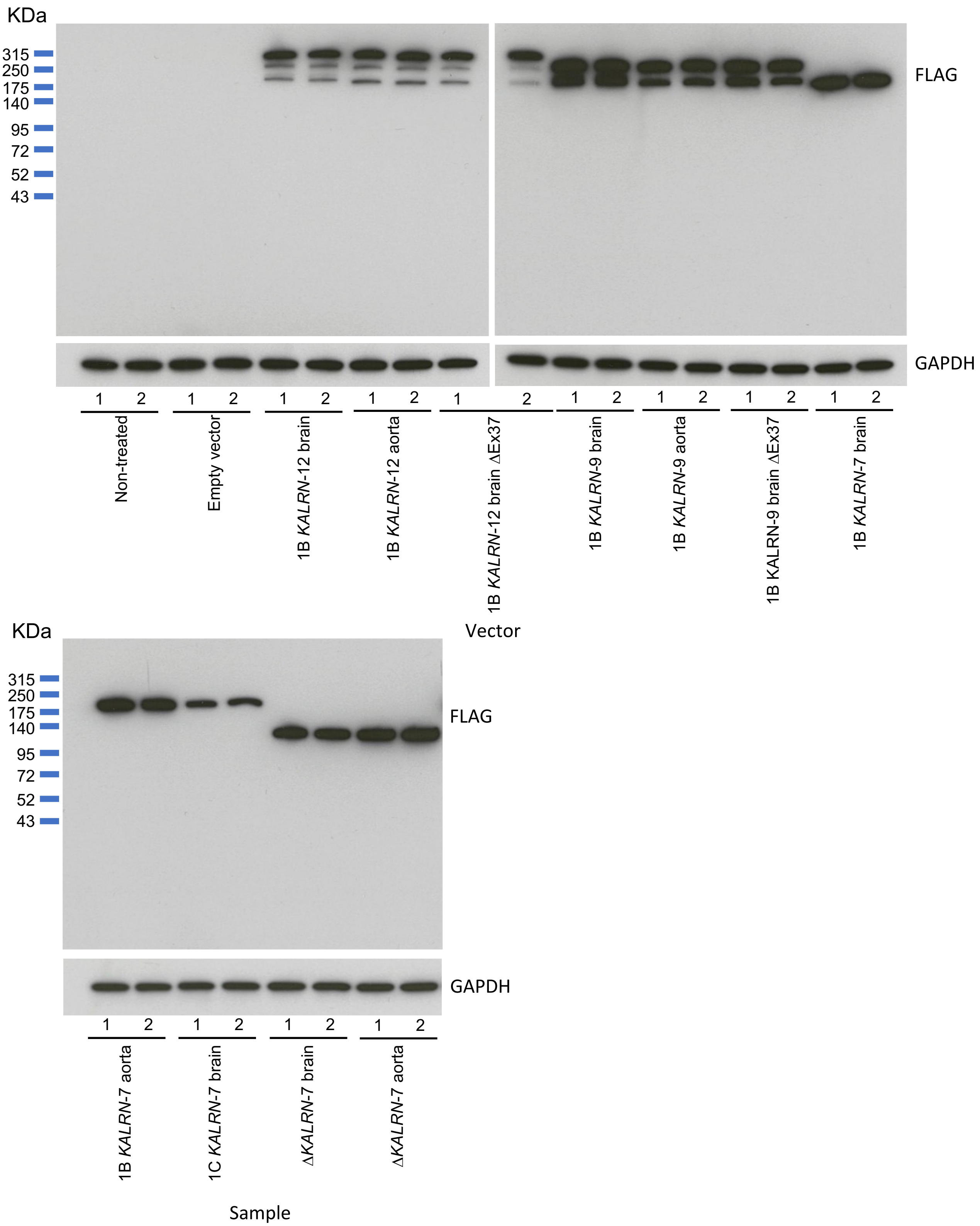

Figure S9 Western blot analysis of FLAG-tagged Kalirin protein expression in cell lysates used for the activated RAC1 and RHOA G-LISA assays presented in figure 6A. Duplicate lysates per vector and non-treated controls are shown. A third replicate per group was similarly analysed and displayed similar levels of protein expression (not shown). Each lane contains 5 μg of total protein. Blots were probed using anti-FLAG antibody then stripped and re-probed with anti-GAPDH antibody to confirm equal protein loading.

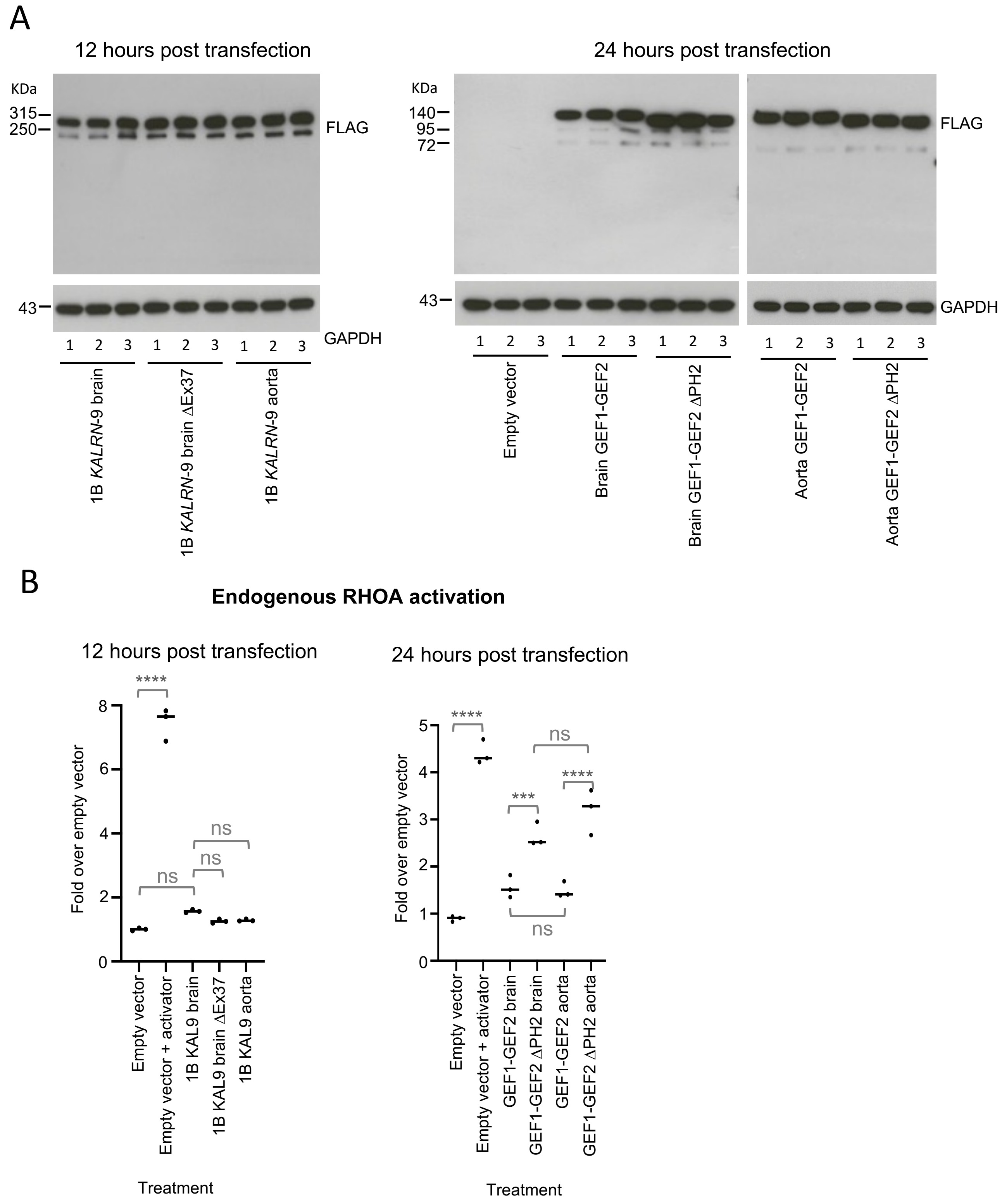

Figure S10. Exogenous Kalirin protein expression and their effects on endogenous RHOA activation in transfected HEK293T cells. (A). Western blot analysis of FLAG-tagged Kalirin proteins in cell lysates (5 μg of total protein per sample) at 12 or 24 hours after triplicate plasmid transfections per group. Cells were serum starved from transfection. Similar levels of FLAG-tagged protein were detected in all KAL vector groups. GAPDH levels were assessed to confirm equal protein loading. (B). Levels of activated RHOA in the same cell lysates as assessed by G-LISA (Cytoskeleton). To confirm that the cells were responsive to RHOA activation, empty vector transfected cells were treated with 1 μg/m Rho Activator II (CN03, Cytoskeleton) 3 hours prior to harvest. One-way ANOVA with Tukey's multiple comparisons test were performed using GraphPad Prism 10 for macOS (V10.3.1). Adjusted *p*-values for pairwise comparisons are shown. ***p=0.0001, ****p<0.0001. ns=non-significant.

Figure S11

A

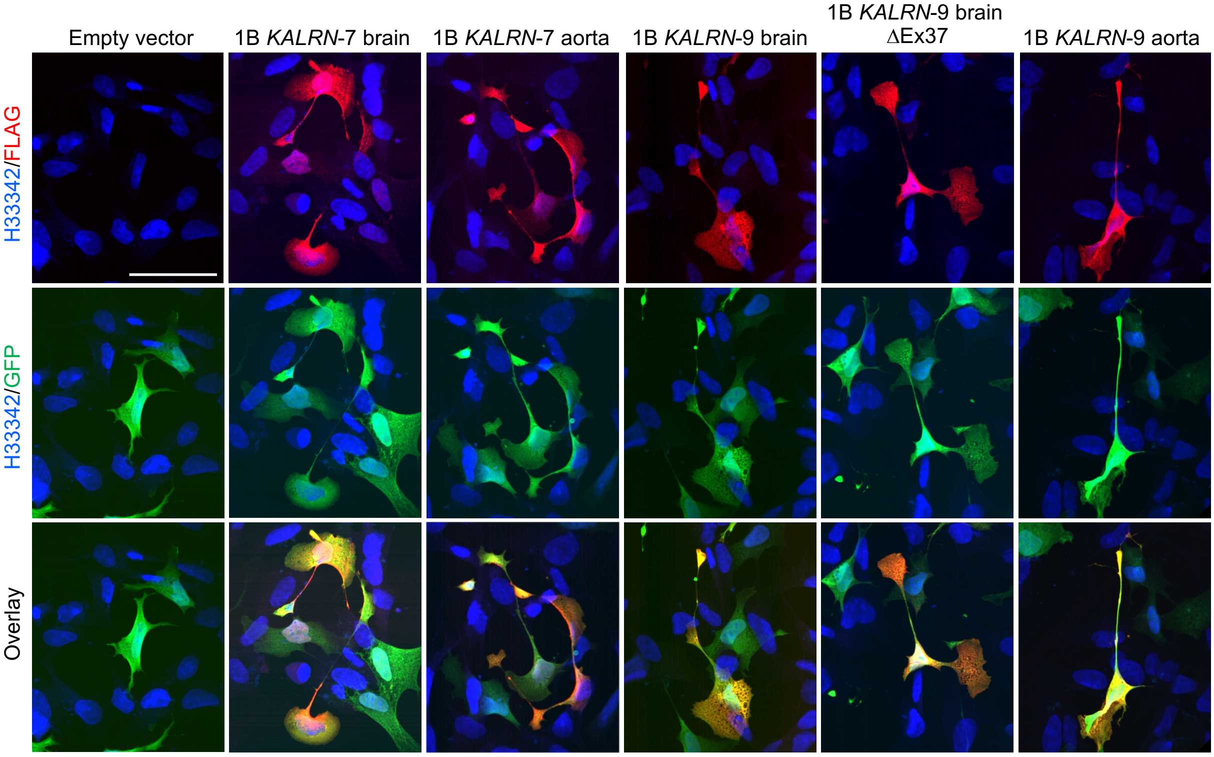

B

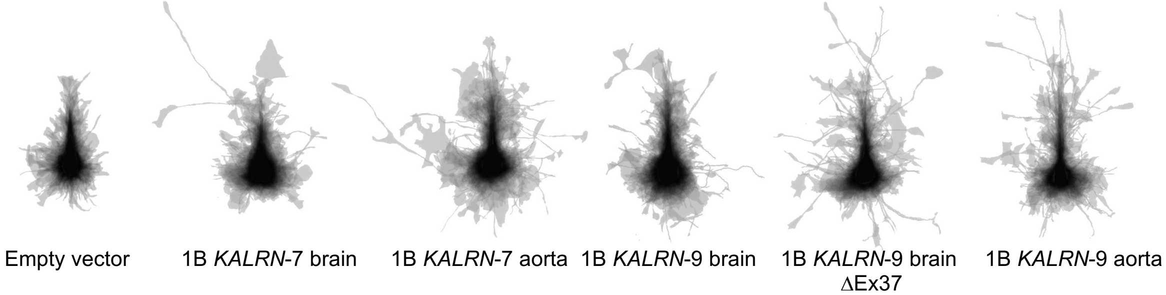

C

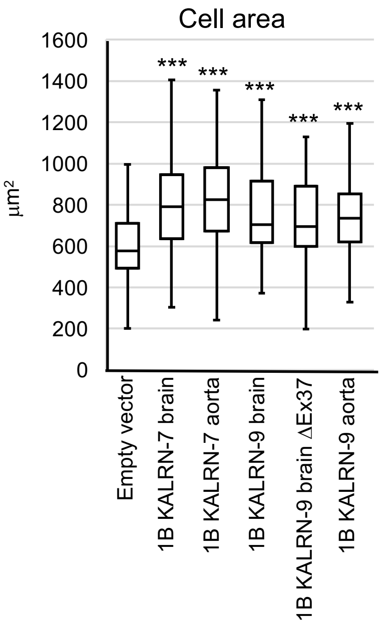

Independent-Samples Kruskal-Wallis Test	
Total N	587
Test Statistic	64.963[a]
Degree Of Freedom	5
Asymptotic Sig.(2-sided test)	1.0E-12

a. The test statistic is adjusted for ties.

Comparison	Test Statistic	Std. Error	Std. Test Statistic	Sig.	Adj. Sig.[a]
EV vs. 1B *KALRN*-7 brain	-150.557	23.753	-6.338	2.3E-010	3.5E-009
EV vs. 1B *KALRN*-7 aorta	-173.372	23.753	-7.299	2.9E-013	4.3E-012
EV vs. *KALRN*-9 brain	-128.013	25.047	-5.111	3.25E-007	4.8E-006
EV vs. *KALRN*-9 brain ΔEx37	-107.673	23.693	-4.545	5.55E-006	8.3E-005
EV vs. *KALRN*-9 aorta	-124.702	23.753	-5.250	1.51E-007	2.3E-006

Figure S11. Effects of brain and aorta Kalirin-7 and Kalirin-9 proteoforms on SH-SY5Y cell morphology 12 hours post transfection (A) Confocal immunofluorescence analysis of FLAG-tagged Kalirin proteoform expression in transfected SH-SY5Y cells. Each of the Kalirin isoforms was detected throughout the cytoplasm and outgrowing neurite tips. All groups were co-transfected with a plasmid that expresses a cytoplasmic EGFP, to assist with cell visualisation and tracing. Scale bar = 50 μm. (B) Overlay of the cell outlines for 82-100 randomly selected cells for each group showing increased cellular area and neurite projections in *KALRN* vector transfected groups compared to empty vector (EV) controls. (C) Morphometric analysis showing significant increases in cell area caused by each of the Kalirin proteoforms. Independent-Samples Kruskal-Wallis Tests were performed using SPSS. Asymptotic *p*-values (2-sided tests) after Bonferroni multiple test correction for pairwise comparisons are shown. ***p<0.001 vs. empty vector.

One-way ANOVA

Table Analyzed	Neurite length per cell	Neurite trunks per cell	Neurite branches
Data sets analyzed	A-F	A-F	A-F
ANOVA summary			
F	59.01471502	6.406	19.21
P value	<0.0001	0.004	<0.0001
P value summary	****	**	****
Significant diff. among means (P < 0.05)?	Yes	Yes	Yes
R squared	0.960921418	0.7275	0.889
Brown-Forsythe test			
F (DFn, DFd)	0.669821498894876 (5, 12)	0.3847 (5, 12)	0.6399 (5, 12)
P value	0.6539	0.8499	0.674
P value summary	ns	ns	ns
Are SDs significantly different (P < 0.05)?	No	No	No

Data set ID	Name
A	Empty vector
B	1B KAL7 brain
C	1B KAL7 aorta
D	1B KAL9 brain
E	1B KAL9 brain DEx37
F	1B KAL9 aorta

Multiple comparisons

Number of families 1
Number of comparisons per family 15
Alpha 0.05

Tukey's multiple comparisons test	Mean Diff.	95.00% CI of diff.	Below threshold?	Summary	Adjusted P Value	Data sets
**Neurite length per cell**						
Empty vector vs. 1B KAL7 brain	-133.6607531	-171.1249832 to -96.19652302	Yes	****	<0.0001	A-B
Empty vector vs. 1B KAL7 aorta	-131.6957671	-169.1599972 to -94.23153702	Yes	****	<0.0001	A-C
Empty vector vs. 1B KAL9 brain	-180.8864571	-218.3506872 to -143.4222270	Yes	****	<0.0001	A-D
Empty vector vs. 1B KAL9 brain ΔEx37	-97.60947843	-135.0737085 to -60.14524835	Yes	****	<0.0001	A-E
Empty vector vs. 1B KAL9 aorta	-100.8135038	-138.2777338 to -63.34927368	Yes	****	<0.0001	A-F
1B KAL7 brain vs. 1B KAL7 aorta	1.964986	-35.49924408 to 39.42921608	No	ns	>0.9999	B-C
1B KAL7 brain vs. 1B KAL9 brain	-47.225704	-84.68993408 to -9.761473917	Yes	*	0.0114	B-D
1B KAL7 brain vs. 1B KAL9 brain ΔEx37	36.05127467	-1.412955417 to 73.51550475	No	ns	0.0618	B-E
1B KAL7 brain vs. 1B KAL9 aorta	32.84724933	-4.616980750 to 70.31147942	No	ns	0.099	B-F
1B KAL7 aorta vs. 1B KAL9 brain	-49.19069	-86.65492008 to -11.72645992	Yes	**	0.0085	C-D
1B KAL7 aorta vs. 1B KAL9 brain ΔEx37	34.08628867	-3.377941417 to 71.55051875	No	ns	0.0826	C-E
1B KAL7 aorta vs. 1B KAL9 aorta	30.88226333	-6.581966750 to 68.34649342	No	ns	0.1312	C-F
1B KAL9 brain vs. 1B KAL9 brain ΔEx37	83.27697867	45.81274858 to 120.7412087	Yes	****	<0.0001	D-E
1B KAL9 brain vs. 1B KAL9 aorta	80.07295333	42.60872325 to 117.5371834	Yes	***	0.0001	D-F
1B KAL9 brain ΔEx37 vs. 1B KAL9 aorta	-3.204025333	-40.66825542 to 34.26020475	No	ns	0.9997	E-F
**Neurite trunks per cell**						
Empty vector vs. 1B KAL7 brain	-2.887	-4.856 to -0.9187	Yes	**	0.0036	A-B
Empty vector vs. 1B KAL7 aorta	-2.826	-4.795 to -0.8580	Yes	**	0.0043	A-C
Empty vector vs. 1B KAL9 brain	-1.733	-3.702 to 0.2351	No	ns	0.097	A-D
Empty vector vs. 1B KAL9 brain ΔEx37	-1.98	-3.949 to -0.01149	Yes	*	0.0484	A-E
Empty vector vs. 1B KAL9 aorta	-1.712	-3.681 to 0.2561	No	ns	0.1028	A-F
1B KAL7 brain vs. 1B KAL7 aorta	0.0607	-1.908 to 2.029	No	ns	>0.9999	B-C
1B KAL7 brain vs. 1B KAL9 brain	1.154	-0.8148 to 3.122	No	ns	0.4111	B-D
1B KAL7 brain vs. 1B KAL9 brain ΔEx37	0.9072	-1.061 to 2.876	No	ns	0.6433	B-E
1B KAL7 brain vs. 1B KAL9 aorta	1.175	-0.7937 to 3.143	No	ns	0.3932	B-F
1B KAL7 aorta vs. 1B KAL9 brain	1.093	-0.8755 to 3.062	No	ns	0.4648	C-D
1B KAL7 aorta vs. 1B KAL9 brain ΔEx37	0.8465	-1.122 to 2.815	No	ns	0.7021	C-E
1B KAL7 aorta vs. 1B KAL9 aorta	1.114	-0.8544 to 3.083	No	ns	0.4458	C-F
1B KAL9 brain vs. 1B KAL9 brain ΔEx37	-0.2465	-2.215 to 1.722	No	ns	0.9979	D-E
1B KAL9 brain vs. 1B KAL9 aorta	0.02106	-1.947 to 1.990	No	ns	>0.9999	D-F
1B KAL9 brain ΔEx37 vs. 1B KAL9 aorta	0.2676	-1.701 to 2.236	No	ns	0.9969	E-F
**Branches per neurite trunk**						
Empty vector vs. 1B KAL7 brain	-11.94	-17.34 to -6.549	Yes	****	<0.0001	A-B
Empty vector vs. 1B KAL7 aorta	-12.01	-17.40 to -6.611	Yes	****	<0.0001	A-C
Empty vector vs. 1B KAL9 brain	-13.18	-18.58 to -7.787	Yes	****	<0.0001	A-D
Empty vector vs. 1B KAL9 brain ΔEx37	-6.543	-11.94 to -1.147	Yes	*	0.015	A-E
Empty vector vs. 1B KAL9 aorta	-7.166	-12.56 to -1.770	Yes	**	0.0078	A-F
1B KAL7 brain vs. 1B KAL7 aorta	-0.06207	-5.458 to 5.334	No	ns	>0.9999	B-C
1B KAL7 brain vs. 1B KAL9 brain	-1.238	-6.634 to 4.158	No	ns	0.9675	B-D
1B KAL7 brain vs. 1B KAL9 brain ΔEx37	5.402	0.005741 to 10.80	Yes	*	0.0497	B-E
1B KAL7 brain vs. 1B KAL9 aorta	4.779	-0.6171 to 10.17	No	ns	0.0943	B-F
1B KAL7 aorta vs. 1B KAL9 brain	-1.176	-6.572 to 4.220	No	ns	0.9738	C-D
1B KAL7 aorta vs. 1B KAL9 brain ΔEx37	5.464	0.06781 to 10.86	Yes	*	0.0466	C-E
1B KAL7 aorta vs. 1B KAL9 aorta	4.841	-0.5550 to 10.24	No	ns	0.0886	C-F
1B KAL9 brain vs. 1B KAL9 brain ΔEx37	6.64	1.244 to 12.04	Yes	*	0.0135	D-E
1B KAL9 brain vs. 1B KAL9 aorta	6.017	0.6212 to 11.41	Yes	*	0.0261	D-F
1B KAL9 brain ΔEx37 vs. 1B KAL9 aorta	-0.6229	-6.019 to 4.773	No	ns	0.9986	E-F

Figure S12. One-way ANOVA with multiple comparisons for figure 7B. Statistical tests were performed using GraphPad Prism 10 for macOS (V10.3.1). KAL = *KALRN.*
